# Isotype-dependent shifts of natural autoantibody profiles in systemic lupus erythematosus-associated serological groups

**DOI:** 10.3389/fimmu.2026.1936421

**Published:** 2026-10-30

**Authors:** Dávid Szinger, Katalin Böröcz, Diána Simon, Alain Le Roux, Szabina Erdő-Bonyár, Nancy Zeineddine, Péter Németh, Timea Berki

**Affiliations:** Department of Immunology and Biotechnology, Clinical Centre, Medical School, University of Pécs, Pécs, Hungary

**Keywords:** anti-dsDNA, autoimmune diagnostics, immune homeostasis, natural autoantibody, seroreactivity, systemic lupus erythematosus (SLE)

## Abstract

**Objectives:**

Homeostatic natural autoantibodies (nAAbs) are proposed regulators of inflammation and autoimmunity, studied mainly as natural IgM. Whether pathological autoantibody (pAAb) expansion in systemic lupus erythematosus (SLE)-associated serology is mirrored by isotype-divergent nAAb changes has rarely been examined.

**Methods:**

pAAb positivity and co-positivity ratios (exact 95% confidence intervals) and intersection (UpSet) analyses were derived from a regional diagnostic cohort (Southern Transdanubia, Hungary, 2014–2024; 128,847 enzyme-linked immunosorbent assay (ELISA) results, 73,747 pseudonymised patients). The dominant patterns defined anti-double-stranded DNA (a-dsDNA)/anti-nucleosome-, anti-SSA/Ro- and anti-cardiolipin/β2-glycoprotein I (a-CL/β2GPI)-dominant and co-positive groups, each verified against serological follow-up. In 110 residual sera, IgG and IgM nAAbs against citrate synthase (CS) and heat shock proteins (HSP) 60 and 70 were measured by ELISA and compared by Kruskal–Wallis and Dunn–Holm tests.

**Results:**

Of a-dsDNA IgG-positive patients, 30.36% carried an additional disease-specific pAAb, along two largely separate axes, antiphospholipid (15.90%) and extractable nuclear antigen-associated (16.51%); anti-neutrophil cytoplasmic antibody co-positivity was rare (3.02%), and 42.01% showed a-nucleosome co-reactivity. a-CS IgM was reduced in every pAAb-positive group (all p_Holm_ < 0.001), whereas increased a-CS, a-HSP60 and a-HSP70 IgG levels were confined to a-dsDNA/a-nucleosome-containing (chromatin-reactive) profiles (a-CS IgG, p_Holm_ ≤ 0.012). These shifts were robust to sex-, age- and measurement-year adjustment.

**Conclusion:**

Isotype-resolved nAAb profiling anchored in diagnostic serology revealed a counter-directional shift (IgM↓ across pAAb-positive groups, IgG↑ in chromatin-reactive profiles). These hypothesis-generating findings are consistent with impaired homeostatic IgM-mediated regulation and with class-switched IgG nAAb reactivity as a marker of B-cell activation, and warrant clinical and functional validation in prospective cohorts.

## Introduction

Serological characterisation of systemic lupus erythematosus (SLE) remains complex due to the progressive breakdown of immune tolerance, reduced thresholds for B-cell activation and differentiation, and the subsequent expansion of pathological autoantibodies (pAAb) (1–5). At the diagnostic level, parallel reactivity for extractable nuclear antigen (ENA) panel components, and antiphospholipid (aPL) antibodies is not uncommon in anti-double-stranded DNA (a-dsDNA)-positive samples (the prefix “a-” denotes “anti-” throughout) and can be explained by progressive diversification through epitope spreading (6–8). Although a-dsDNA IgG antibodies are key disease-specific biomarkers, they do not fully capture the biological heterogeneity of SLE (6–12); this complexity, inherent to the interconnected nature of the immune system, also extends to the natural autoantibody (nAAb) compartment (13–17).

Present also in healthy individuals, homeostatic nAAbs recognise conserved oxidation- and apoptosis-associated self-structures, including apoptotic-cell determinants, heat shock proteins (HSPs), and mitochondrial enzymes such as citrate synthase (CS) (18–23). Therefore, convergent pathomechanisms characteristic of systemic autoimmune rheumatic diseases (SARDs) (2–5, 10, 24, 25), may not only promote pAAb expansion, but also indirectly modulate nAAb production (16, 26, 27). Especially, in SLE, where defective clearance, dysregulated cytokine signalling, and broad B-cell activation converge (2–5, 10, 24, 25), pAAb expansion may be accompanied by measurable remodelling of the nAAb network (2–5, 10, 24, 25). Despite growing clinical evidence (13, 14, 28–30), nAAb alterations remain poorly characterised from a sero-diagnostic perspective, particularly in an isotype-diversified manner and in relation to compound pAAb phenotypes (9–12, 16, 31–33).

Therefore, we addressed two questions in a two-tier design. First, using a decade-long routine diagnostic cohort from Southern Transdanubia, Hungary (2014–2024; enzyme-linked immunosorbent assay [ELISA] results), we asked which pAAb patterns, including chromatin co-reactivity with a-nucleosome antibodies, accompany a-dsDNA positivity and how frequently they occur. Second, we asked whether the dominant serological patterns identified in this way are cross-sectionally associated with coordinated, isotype-divergent nAAb alterations, using paired IgG/IgM nAAb profiling against CS, HSP60 and HSP70 in a targeted, hypothesis-generating sub-study. In contrast to earlier studies, which mostly assessed IgM nAAbs in clinically defined patient groups, IgG and IgM nAAbs were measured in parallel, and the sub-study groups were derived from the observed serological structure of the diagnostic cohort and verified against the serological follow-up of each patient.

## Materials and methods

To investigate associations between pAAb and nAAb networks, we applied a two-tier analytical framework combining population-level retrospective analysis of diagnostically relevant pAAbs with cross-sectional, isotype-diversified nAAb profiling. This approach was used to assess whether increased pAAb burden in polyreactive samples was accompanied by consistent nAAb trends.

### pAAb-based sample clustering

To characterise SLE-associated serological diagnostic patterns in Southern Transdanubia, Hungary, we performed a retrospective analysis of major autoimmune diagnostic panels from 2014–2024, comprising 128,847 ELISA tests from 73,747 de-duplicated, pseudonymised patients aged 18–80 years (65.67% women; median age 53 years, interquartile range 39–67; **Supplementary Table S1**). The analysis focused on a-dsDNA IgG-associated pAAb co-positivity, defined as ≥2 disease-specific pAAbs per sample. Included main diagnostic panels were: nuclear autoimmunity (antinuclear antibody [ANA] screen, a-dsDNA IgG, a-nucleosome IgG), antiphospholipid (aPL) autoantibodies (anti-cardiolipin/anti-β2-glycoprotein I [a-CL/β2GPI] IgG/IgM, anti-prothrombin [a-PT] IgG/IgM), extractable nuclear antigens (ENA IgG screen, a-SSA/Ro, a-SSB/La, a-Sm, a-U1-RNP, a-Scl-70, a-Jo-1), and vasculitis panel (anti-neutrophil cytoplasmic antibodies [ANCAs]: anti-proteinase 3 [a-PR3] IgG, anti-myeloperoxidase [a-MPO] IgG). Positivity was determined using laboratory-validated, assay-specific cut-offs (period-specific for the ANA screening and ENA antigen assays, which changed during the study period), as detailed in **Supplementary Table S1**; ENA screening results were used for testing frequencies only. For the standard-curve-based ELISAs of the routine pAAb panel (predominantly ORGENTEC by Sebia kits), results above the upper limit of quantification are, according to the laboratory standard operating procedure agreed with the requesting clinicians, reported as the nominal value of the highest kit standard rather than extrapolated. As all pAAb analyses were based on cut-off-defined positivity, quantitative values above the measuring range did not influence the results; for display, values were capped at the upper reporting limit (**Supplementary Figure S1**).

For the overall assessment of a-dsDNA IgG–associated serological poly-reactivity, major quantitative ELISA-based autoimmune markers of Sjögren’s disease, vasculitis and aPL routinely used in our laboratory were included. Co-positivity was defined solely by quantitative ELISA results; panels primarily assessed by immunoblot methods were excluded (e.g., extended autoimmune liver disease, myositis-associated, systemic sclerosis-associated, and paraneoplastic neurological antibody panels).

Representativeness of this regional demographic analysis was supported by our accredited Autoimmune Routine Diagnostics Laboratory (MSZ EN ISO 15189:2023), which serves as a regional referral centre for systemic autoimmune rheumatic disease (SARD) diagnostics in Southern Transdanubia. The study was conducted in accordance with the World Medical Association Declaration of Helsinki and was approved by the Regional Research Ethics Committee of the Clinical Center of the University of Pécs (2-PTERKEB2026/1; residual-specimen diagnostic research programme licences 5726-PTE, 2015, and 5726/8216-PTE, 2020) as a retrospective, non-invasive analysis of anonymised routine diagnostic data/residual samples without patient contact.

Study workflow:

- Autoimmune (SARD) laboratory testing frequencies (single analyte/co-testing), as an indicator of laboratory diagnostic burden: test utilisation was quantified using total, non-deduplicated measurement counts per patient, reflecting clinician-driven diagnostic workflow rather than pAAb positivity frequency.
- pAAb positivity and co-positivity ratios (%), used as indicators of regional diagnostic pAAb burden within the tested patient population: de-duplicated patient counts were normalised using two complementary denominator strategies:
  - First, overall positivity rates were calculated relative to the number of patients tested within the corresponding diagnostic panel (screening and/or antigen-specific assay) (**Supplementary Table S1**). Co-positivity ratios of co-tested patients were calculated relative to the number of patients tested for a-dsDNA IgG and the corresponding panel in the same sample. This conservative approach uses larger denominators and therefore yields lower, screening-level positivity estimates. Because the cohort comprises patients referred for autoimmune diagnostic testing rather than a general-population sample, these values represent diagnostic test positivity rates within a referred clinical population and should not be interpreted as general-population or disease prevalence.
  - Second, co-positivity rates were calculated within the a-dsDNA IgG-positive subset, using the number of a-dsDNA IgG-positive patients as denominator. This restricted “within-a-dsDNA-positive” framework uses smaller denominators and therefore yields higher relative co-positivity estimates, reflecting the enrichment of additional autoreactivities amongst a-dsDNA-positive samples. Co-positivity with a-nucleosome IgG, which targets the same antigen (chromatin), is reported separately as chromatin co-reactivity.
- Confidence interval (CI)–based stratification: markers were assigned to high- (I), intermediate- (II) and low-frequency (III) tiers on the basis of their positivity ratios and the overlap of their exact 95% CIs (single-analyte and co-testing ratios: ≥ 5%, 1–4.99% and < 1%; within the a-dsDNA IgG-positive subset: ≥ 10%, 3–9.99% and < 3%). Single-analyte and co-testing datasets were analysed separately due to differing scales and distributions.

The formulas and exact numbers of tested/co-tested and positive/co-positive patients, stratified by autoimmune diagnostic marker–based groups, used for the calculations are provided in **Supplementary Table S1**. For each analyte, positive results (or, for co-positivity, a-dsDNA IgG-positive samples that were also positive for the given marker) were identified first; results were then ordered by patient (health insurance identification number, which was subsequently de-coupled from all patient data during pseudonymisation) and test date, and only the first chronological qualifying result per patient was counted.

Although rheumatoid arthritis represents an undoubtedly major systemic autoimmune disease group with substantial diagnostic demand, rheumatoid arthritis-associated testing was excluded from the present analysis due to the diagnostic complexity of the “rhupus” overlap phenomenon (34, 35).

### pAAb intersection (UpSet) analysis

To visualise the overlap structure of the ELISA-based pAAb specificities simultaneously, exclusive intersections were computed at the de-duplicated patient level for a-dsDNA IgG, a-SSA/Ro, a-SSB/La, a-Sm, a-U1-RNP, a-Scl-70, a-Jo-1, a-PR3 and a-MPO IgG, together with a-CL/β2GPI (IgG and/or IgM) as the dominant co-positivity axis identified above. As ANCAs were assessed by antigen-specific ELISA, cytoplasmic (c-ANCA) and perinuclear (p-ANCA) ANCA reactivities are represented by a-PR3 and a-MPO IgG, respectively. a-nucleosome IgG was included as a separate specificity in the population-level analysis (a) but was not counted as an additional specificity within the a-dsDNA IgG-positive subset (b), as a-nucleosome and a-dsDNA antibodies target the same antigen (chromatin) and their co-occurrence reflects a single chromatin-directed reactivity rather than an additional autoreactivity (36, 37); ANA and ENA screening results were not included, as only antigen-specific reactivities were counted. To keep every combination within a single sample, each patient was represented by one sample, selected in the same order as above (Positive results were flagged first, followed by identification of co-positive samples. Records were then ordered by health insurance identifier and test date. Subsequently, all results were decoupled from the original patient-specific identifiers, and the analyses were performed exclusively on a pseudonymised dataset). Two complementary populations were analysed: (a) all patients positive for at least one of these specificities, represented by their first chronological sample with two or more positive specificities or, if there was none, by their first positive sample; and (b) the a-dsDNA IgG-positive subset (n = 2,447), represented by their first chronological a-dsDNA IgG-positive sample that was also positive for at least one additional specificity, or classified as a-dsDNA IgG-only if no such sample existed. Each patient was thus assigned to exactly one intersection (exclusive combination), and specificities not requested for a given sample were considered negative. Intersections were visualised as UpSet plots; the complete intersection data are provided in **Supplementary Table S2**. To summarise the main serological axes, co-positive a-dsDNA IgG-positive patients were additionally grouped into aPL (a-CL/β2GPI IgG/M), Ro/La (a-SSA/Ro and/or a-SSB/La) and Sm/U1-RNP (a-Sm and/or a-U1-RNP) reactivities, with the remaining specificities (a-PR3, a-MPO, a-Scl-70, a-Jo-1) combined as “others”, and displayed as Venn diagrams separately for double-positive (one additional antibody) and triple- or higher-positive (two or more additional antibodies) patients.

### Sex distribution of pAAb positivity

To assess the contribution of sex to diagnostic pAAb positivity, the proportion of women was determined in the whole cohort, amongst patients tested within each diagnostic panel and amongst positive patients, using the same denominators and first-qualifying-result selection as described above; sex and age were taken from each patient’s first record. Positivity in women versus men was expressed as odds ratios (ORs) adjusted for age by Mantel–Haenszel stratification (18–39, 40–59 and 60–80 years), with Robins–Breslow–Greenland 95% CIs and Cochran–Mantel–Haenszel tests. Amongst a-dsDNA IgG-positive patients, the proportion of women across groups with no, one, or two or more additional autoantibodies (first co-positive sample, as in the intersection analysis) was compared using the Cochran–Armitage test for trend, and age between these groups using the Kruskal–Wallis test.

### nAAb profiling

#### Sample selection and storage

Integrating the three points outlined above (i–iii) with basic diagnostic SARD considerations (38–42), serological sub-clusters were established for downstream nAAb measurements. These subsets of samples in our routine diagnostic biobank (−20 °C; 4–5-month extended storage policy) were processed for serology within the post-analytical storage window.

#### nAAb measurements

nAAb measurements were performed over the study period (2014–2023) using the same in-house protocol and internal standard serum; new standard-pool lots were bridged against the previous lot (≤ 20% difference in back-calculated values). IgG and IgM isotype nAAbs directed against citrate synthase (CS) and HSP60/70, were measured using in-house ELISAs, as described previously (26, 43, 44). Results were expressed in arbitrary units per millilitre (AU/mL) relative to in-house calibrated standards; results above the highest calibrator were right-censored at its nominal value, whereas results below the lowest reliably quantifiable calibrator were retained as measured.

#### Serological group definition and verification

Sample numbers for nAAb measurements: a-CL/β2GPI IgG/IgM (n = 16), a-CL/β2GPI IgG with a-dsDNA/a-nucleosome IgG (n = 12), a-dsDNA/a-nucleosome IgG (n = 14), a-SSA/Ro IgG (n = 15), a-dsDNA/a-nucleosome IgG with a-SSA/Ro IgG (n = 14), and seronegative controls (NC, n = 39). Group assignment was verified against the complete diagnostic record of each patient (all 2014–2024 samples of the same patient, linked through the health insurance identification number before pseudonymisation). A group-defining pAAb was accepted if it was positive in the nAAb sample or in at least two samples of the patient; an additional group-defining pAAb (a-dsDNA, a-SSA/Ro or a-CL/β2GPI IgG/IgM) positive in at least two samples led to re-assignment to the corresponding co-positive group. As a-nucleosome (anti-chromatin) antibodies are an SLE-associated specificity with diagnostic sensitivity comparable to or higher than that of a-dsDNA and may precede or replace a-dsDNA positivity (37, 45–47), the a-dsDNA groups were defined by chromatin reactivity (hereafter, a-dsDNA/a-nucleosome groups): samples were retained if a-dsDNA or a-nucleosome IgG positivity (> 20 U/mL) was documented in the nAAb sample or on at least two dates. Three patients fulfilled this criterion by a-nucleosome positivity alone. a-nucleosome positivity was not used as a re-assignment criterion; seven patients of the a-SSA/Ro- (n = 3) and a-CL/β2GPI-dominant (n = 4) groups with documented a-nucleosome IgG positivity (in the nAAb sample or on at least two dates) but without recurrent a-dsDNA positivity therefore remained in their dominant group. When a patient contributed several samples to the same group, only the earliest was analysed (110 samples in total). The complete longitudinal serological results (2014–2024) underlying this review are shown in **Supplementary Figure S1**. Accordingly, the three single-specificity groups are referred to as a-dsDNA/a-nucleosome-, a-SSA/Ro- and a-CL/β2GPI-dominant (collectively, “dominant” groups): they were positive for one of the three group-defining specificities (a-dsDNA/a-nucleosome, a-SSA/Ro or a-CL/β2GPI IgG/IgM) without recurrent positivity for the other two, whereas other reactivities were not exclusion criteria. The six ENA antigens (a-SSA/Ro, a-SSB/La, a-Sm, a-U1-RNP, a-Scl-70 and a-Jo-1) are measured together in one assay run; this panel was therefore available for all patients of the a-SSA/Ro groups, but had been requested for only a minority of patients in the other groups, as had ANCA and a-PT testing. Seronegative controls (NC; so termed for brevity and not intended to imply a healthy reference population) were diagnostic samples negative for all SARD-associated pAAbs tested in or before the nAAb sample, except for two weakly positive ANA screening results (22.7 and 24.5 U/mL); the NC label therefore reflects autoantibody status only, not confirmed clinical health.

#### Age, sex and missing values

Age and sex of each sample were retrieved from the laboratory information system; as not every analyte could be measured in every sample (technical measurement issues or depletion of the residual serum; missing values were not imputed), the number of samples contributing to each comparison is shown in the corresponding figures.

### Statistical analysis

Data processing and descriptive statistics were performed using Microsoft Excel, OriginPro, and JASP. Group differences in nAAb levels were assessed with Kruskal–Wallis tests followed by Dunn’s *post hoc* test, with Holm’s correction applied to all pairwise comparisons within each nAAb analyte and figure (p_Holm_). All tests were two-sided; statistical significance was set at α < 0.05, with trends reported for p < 0.10, and missing values were not imputed. Positivity estimates with exact 95% Clopper–Pearson confidence intervals were used to define diagnostic pAAb strata for downstream nAAb analysis. Exclusive pAAb intersections and the Venn groupings were computed in Python (version 3.11.15; pandas 3.0.2, NumPy 2.4.4), and the data figures were drawn with matplotlib (version 3.10.9). Sex-related analyses (Mantel–Haenszel ORs, Cochran–Mantel–Haenszel and Cochran–Armitage tests), exact confidence intervals, and the sensitivity, year-adjusted and power analyses of the nAAb sub-study were performed in Python using SciPy (version 1.17.1) and NumPy (version 2.4.4).

In the nAAb sub-study, results are reported as medians with interquartile ranges (IQR). Age and sex of the sub-study groups were compared with the Kruskal–Wallis and Mann–Whitney U tests and with the χ² and Fisher’s exact tests, respectively, and the association of nAAb levels with age amongst controls was assessed with Spearman’s rank correlation. To assess potential confounding by sex and age, three sensitivity analyses were performed for all comparisons with seronegative controls: (i) restriction to women; (ii) inclusion of only one (the earliest) sample per patient, as two patients contributed samples to more than one serological group; and (iii) adjustment for age and sex using a rank-based analysis of covariance (linear model on rank-transformed nAAb values with group, age and sex as covariates; Holm correction over the comparisons with controls). In addition, as the nAAb measurements were performed in different years (2014–2023), the year of measurement was added as a covariate to the rank-based model, alone and together with age and sex. To characterise the statistical power of the sub-study, a *post-hoc* sensitivity power analysis was performed (normal approximation for the Mann–Whitney/Dunn comparison; 80% power; two-sided α = 0.05 and the strictest Holm-adjusted α = 0.0083), expressing the smallest detectable effect as the probability that a value in one group exceeds a value in the other [P(X > Y)], together with the equivalent Cohen’s d; observed effects were expressed in the same way [P(X > Y) = U/(n1·n2), from the Mann–Whitney U statistic]. Within this study, the pAAb positivity and intersection analyses of the diagnostic cohort (**Figures 1**–**3**) constitute the primary, descriptive analysis at the regional diagnostic level, whereas the nAAb comparisons (**Figures 4**–**6**) are exploratory and hypothesis-generating.

**Figure 1:**
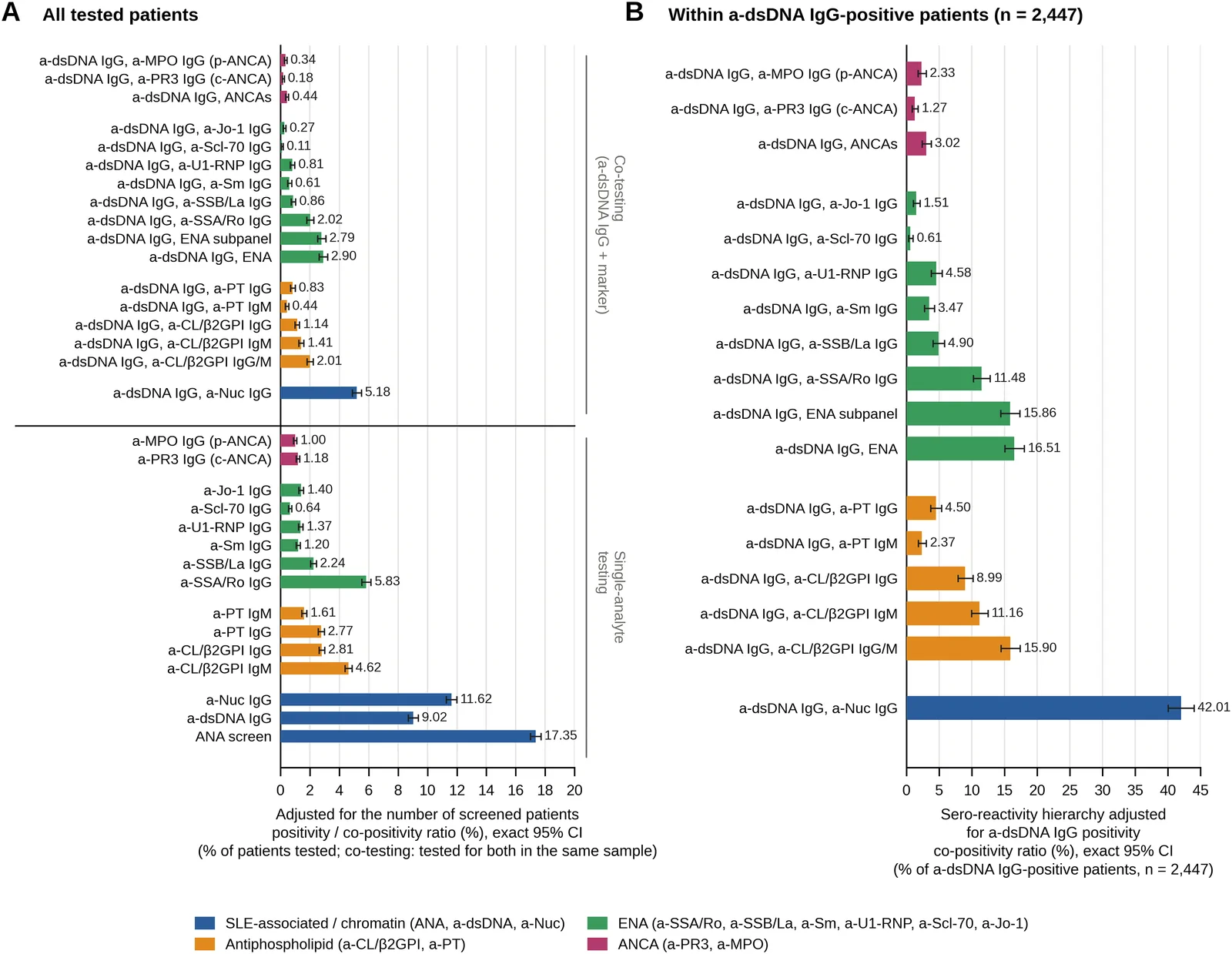
Sero-reactivity hierarchies. **(A)** Adjusted for the number of screened patients: positivity in single-analyte testing (lower part) and co-positivity with a-dsDNA IgG in co-testing (upper part) amongst all tested patients. **(B)** Sero-reactivity hierarchy adjusted for a-dsDNA IgG positivity: co-positivity within the a-dsDNA IgG-positive patients (n = 2,447). **(A)** Bars show positivity ratios calculated relative to the number of patients tested within the corresponding diagnostic panel (single-analyte testing; screening and/or antigen-specific assay), and co-positivity ratios calculated relative to the number of patients co-tested for a-dsDNA IgG and the corresponding panel in the same sample (co-testing); see **Supplementary Table S1**. Bar colours indicate the diagnostic marker group: blue, SLE-associated/chromatin (ANA, a-dsDNA, a-Nuc); orange, antiphospholipid (a-CL/β2GPI, a-PT); green, ENA; magenta, ANCA. ANA screen positivity was defined as > 10 U/mL for 2014–2016 (HYCOR Biomedical) and > 20 U/mL from 2017 (Werfen/Inova); ENA screening is not shown. Co-positivity of a-dsDNA with a-nucleosome IgG represents chromatin co-reactivity, i.e. two antibodies against the same antigen, and is shown separately from co-positivity with additional specificities. **(B)** Bars show co-positivity ratios within the a-dsDNA IgG-positive patients (n = 2,447). The co-testing values in A and B therefore differ: in A, the denominator is the number of patients co-tested for a-dsDNA IgG and the marker in the same sample (e.g., 13,909 for ENA); in B, it is the 2,447 a-dsDNA IgG-positive patients. Error bars in both panels represent exact 95% Clopper–Pearson confidence intervals (CIs). Co-positivity was defined as ≥2 disease-specific pAAbs per sample. Y axis: tested markers or a-dsDNA IgG-centred co-positivity patterns. X axis: positivity or co-positivity ratio (%); the axis titles refer to the corresponding Results subsections (“Adjusted for the number of screened patients” and “Sero-reactivity hierarchy adjusted for a-dsDNA IgG positivity”) and state the denominator. CI supplemented frequencies were stratified into high, intermediate, and low clusters as per **Supplementary Table S1**. a-dsDNA IgG, a-CL/β2GPI IgG/M = co-positivity with a-CL/β2GPI IgG/M, irrespective of isotype (IgG and/or IgM); a-dsDNA IgG with ENA = co-reactivity to any ENA panel antigen; ENA subpanel = co-reactivity to any of the following four antigens: SSA/Ro, SSB/La, Sm, and/or U1-RNP IgG; a-dsDNA IgG with ANCA, co-positivity with PR3/c-ANCA and/or MPO/p-ANCA; a-Nuc, anti-nucleosome. For each analyte, the first chronological qualifying result per patient was counted.

**Figure 2:**
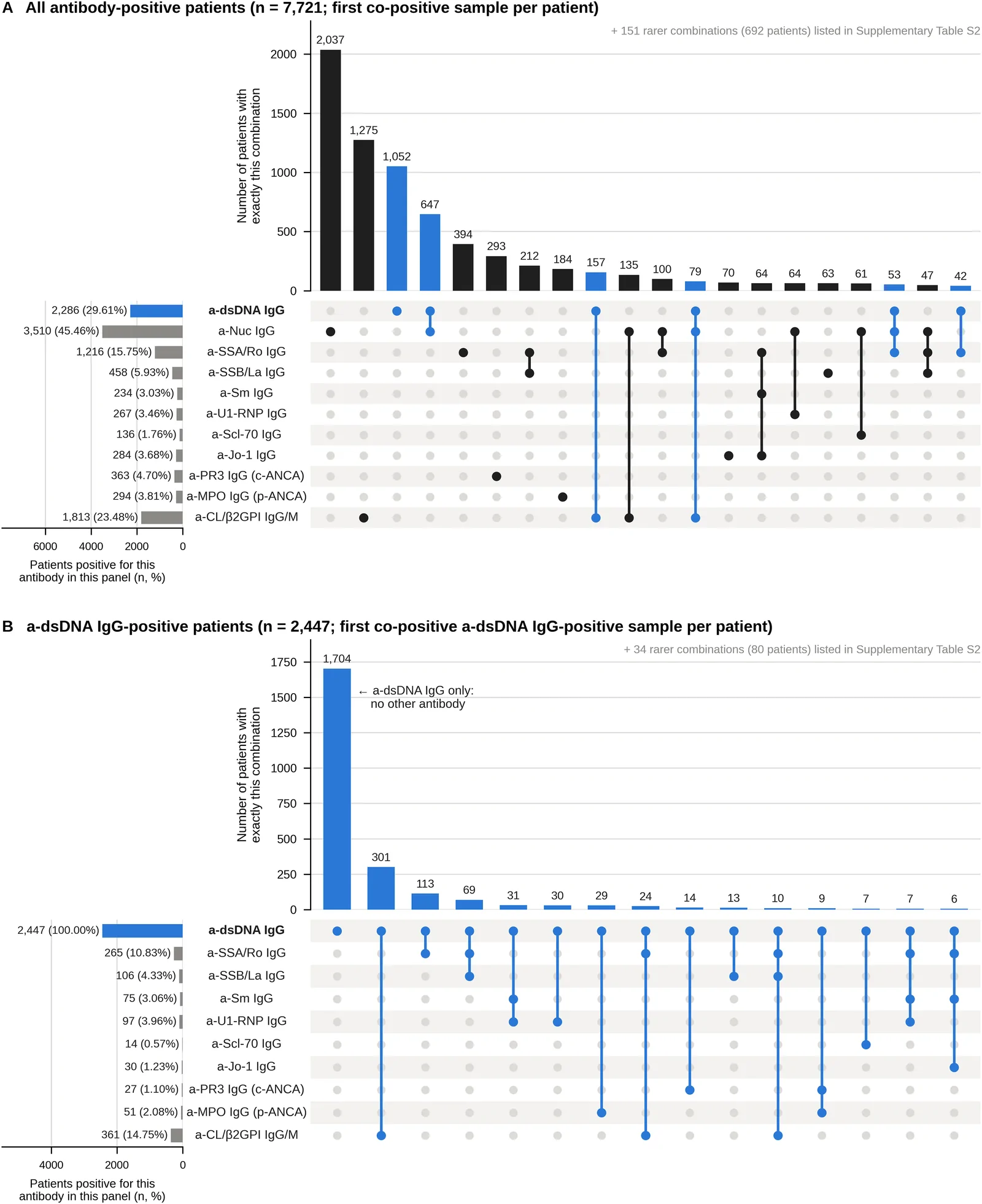
Intersection (UpSet) analysis of pAAb positivity. **(A)** All patients aged 18–80 years positive for at least one of the displayed specificities, including a-nucleosome IgG (n = 7,721), each represented by the first chronological sample with two or more positive specificities or, if none, the first positive sample. **(B)** a-dsDNA IgG-positive patients (n = 2,447), each represented by the first chronological a-dsDNA IgG-positive sample that was also positive for at least one further specificity (otherwise shown as a-dsDNA IgG only). How to read the plot: each column represents one group of patients sharing exactly the same antibody combination. In the dot matrix below the columns, a dark (or blue) dot marks an antibody that was positive in that group and a grey dot an antibody that was negative; the vertical line simply connects the positive antibodies of the same group. The bar above each column gives the number of patients with exactly that combination (each patient is counted only once), and blue indicates combinations that include a-dsDNA IgG. For example, the column in panel A with blue dots for a-dsDNA IgG and a-CL/β2GPI IgG/M (n = 157) comprises patients positive for these two antibodies and for none of the others. The horizontal bars on the left give the number of patients positive for each antibody in the panel (% of the panel population); because one sample per patient is used, these totals are slightly lower than the analyte-by-analyte counts of **Figure 1B**. The 20 **(A)** and 15 **(B)** largest combinations are shown; all remaining combinations are listed in **Supplementary Table S2**. Positivity was defined by laboratory-validated ELISA cut-offs (**Supplementary Table S1**); c-ANCA and p-ANCA correspond to a-PR3 and a-MPO IgG ELISA, respectively; a-CL/β2GPI IgG/M denotes a-CL and/or a-β2GPI IgG and/or IgM. ANA screening was not included; a-nucleosome IgG is shown in panel A only, as in panel B it would represent the same (chromatin) reactivity as a-dsDNA IgG. Specificities not requested for a sample were considered negative.

**Figure 3:**
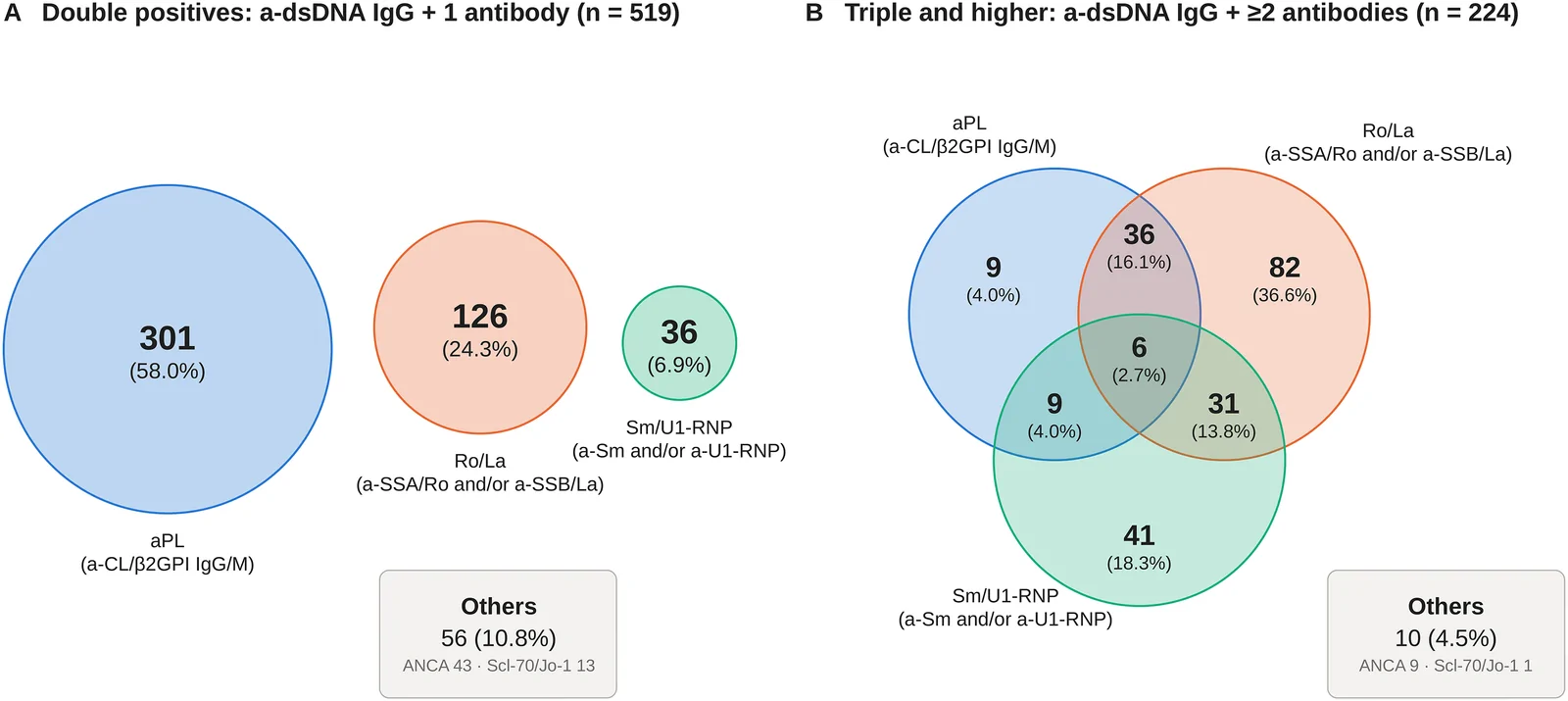
Distribution of the main co-positivity axes amongst a-dsDNA IgG-positive patients with at least one additional antibody (n = 743; first co-positive sample per patient, as in **Figure 2B**). **(A)** Double positives (a-dsDNA IgG plus one additional antibody; n = 519): each patient carries only one additional antibody, so the groups cannot overlap; circle areas are proportional to patient numbers. **(B)** Triple and higher positives (a-dsDNA IgG plus two or more additional antibodies; n = 224): numbers in overlapping areas denote patients positive for antibodies of more than one axis (circles not drawn to scale); a patient positive for two antibodies of the same axis (e.g., a-SSA/Ro and a-SSB/La) is shown within a single circle. aPL, a-CL/β2GPI IgG/M; Ro/La, a-SSA/Ro and/or a-SSB/La; Sm/U1-RNP, a-Sm and/or a-U1-RNP. “Others”: patients whose additional antibodies were exclusively a-PR3 or a-MPO (ANCA), a-Scl-70 or a-Jo-1; additional ANCA, a-Scl-70 or a-Jo-1 reactivity in patients within the circles is not shown separately. Percentages refer to the patients in each panel.

**Figure 4:**
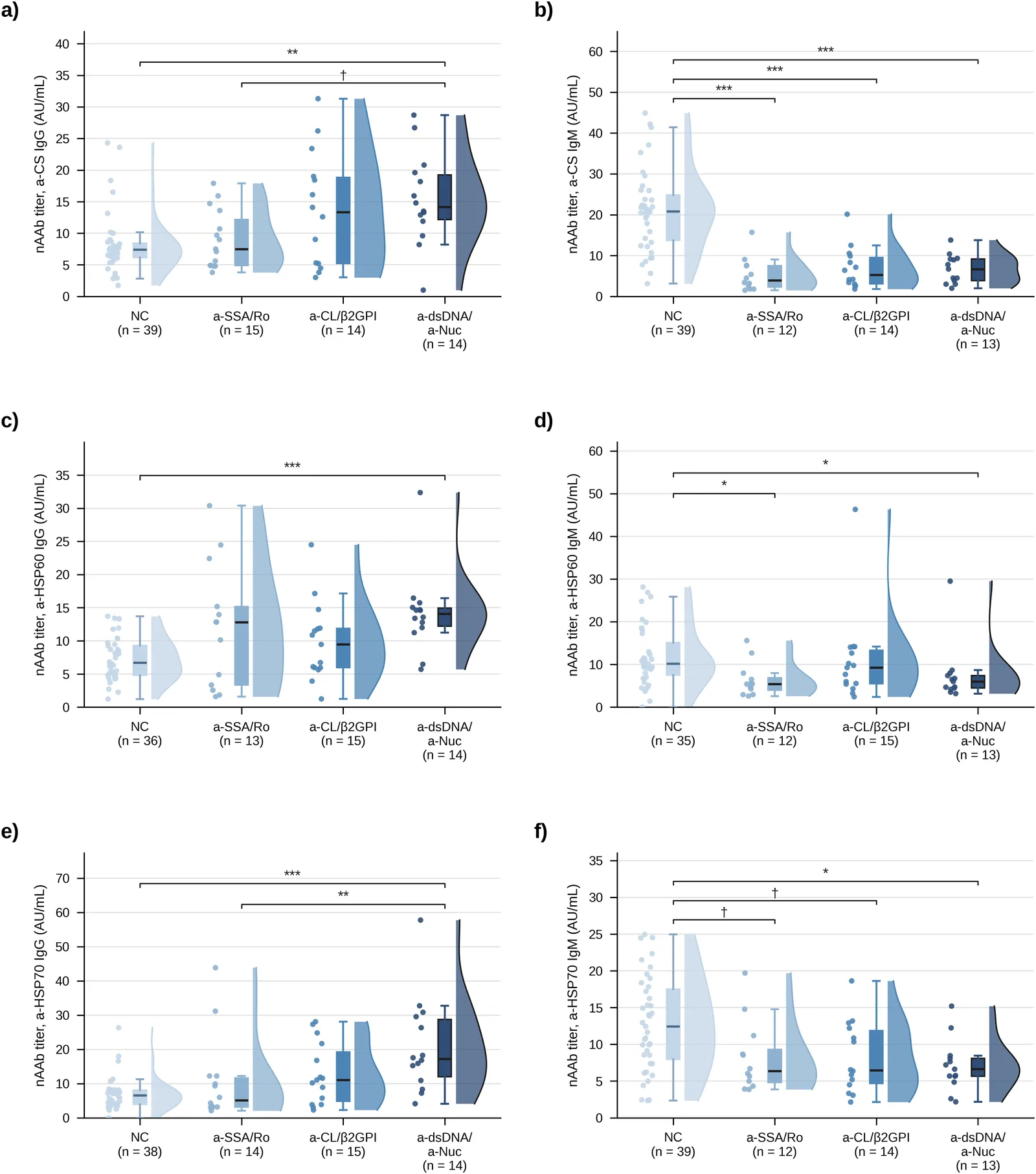
nAAb patterns across single-specificity-dominant serological subgroups. Serum nAAb levels were analysed across pAAb-defined serological groups and seronegative controls. pAAb stratification: a-SSA/Ro-dominant, a-CL/β2GPI-dominant, and a-dsDNA/a-nucleosome-dominant. Panels depict the following analytes: **(A)** a-CS IgG, **(B)** a-CS IgM, **(C)** a-HSP60 IgG, **(D)** a-HSP60 IgM, **(E)** a-HSP70 IgG, and **(F)** a-HSP70 IgM. The group-defining pAAbs (x-axis categories) were a-SSA/Ro IgG, a-CL/β2GPI IgG and/or IgM, and a-dsDNA/a-nucleosome IgG. The y-axis represents serum concentrations (AU/mL) of nAAbs (IgG or IgM, as indicated). Group sizes were as follows: NC (n = 39), a-SSA/Ro (n = 15), a-CL/β2GPI (n = 16), a-dsDNA/a-nucleosome (n = 14); a-dsDNA/a-nucleosome groups (a-dsDNA/a-Nuc in the image) include patients with a-dsDNA and/or a-nucleosome IgG positivity; groups were assigned after review of each patient’s complete diagnostic record (see Methods). The number of samples with a result for each analyte is shown below each group. Data are presented as raincloud plots showing individual data points, box plots (median and interquartile range; whiskers, 1.5 × IQR) and kernel density estimates. Statistical analysis was performed using Kruskal–Wallis tests followed by Dunn’s *post hoc* comparisons with Holm correction. Significant pairwise differences (Holm-adjusted) are indicated as follows: *p_Holm_ ≤ 0.05; **p_Holm_ ≤ 0.01; ***p_Holm_ ≤ 0.001; †p_Holm_ < 0.10.

**Figure 5:**
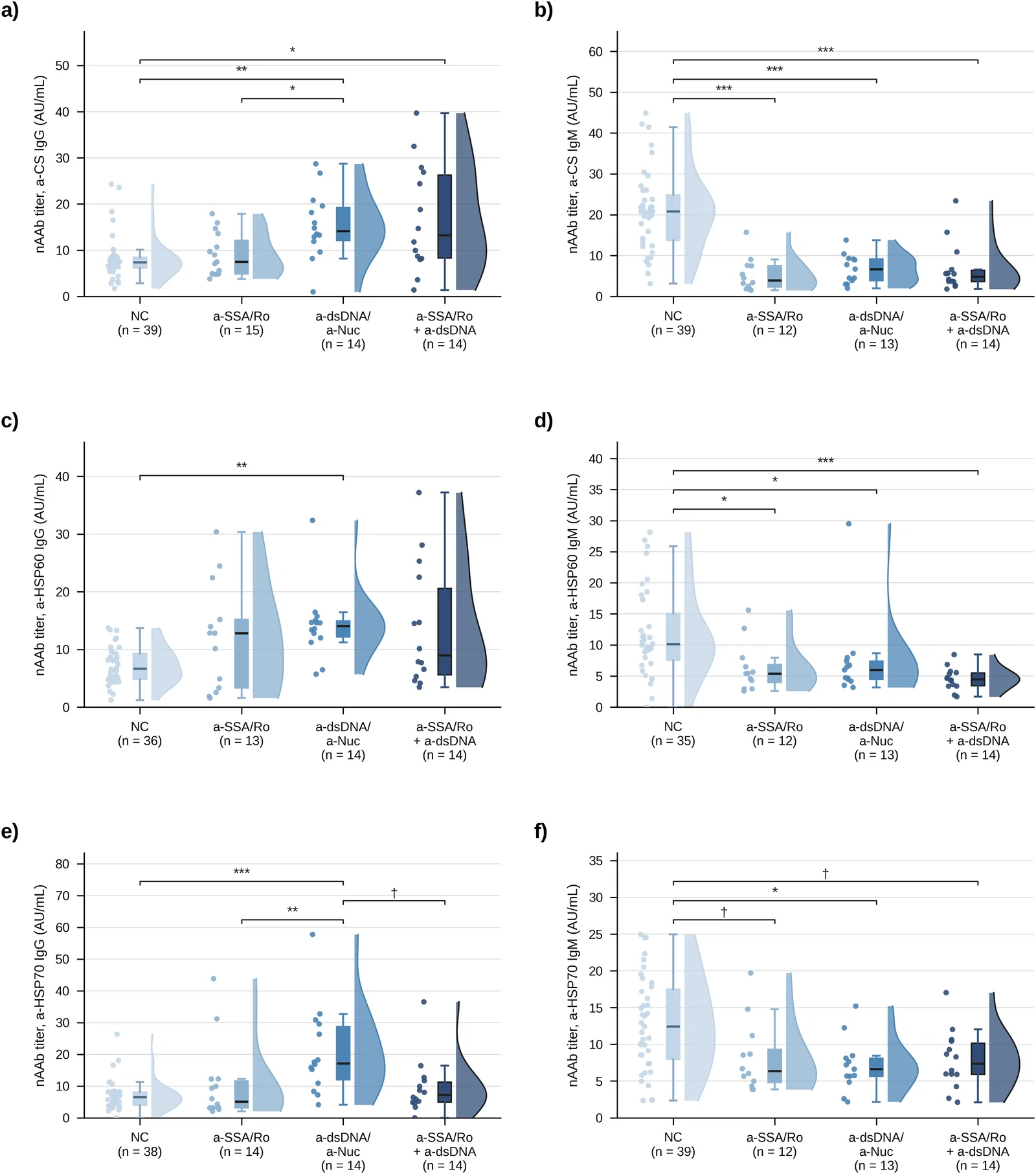
nAAb patterns across dominant and co-positive serological subgroups, with particular focus on the a-SSA/Ro with a-dsDNA co-positive subgroup. Serum nAAb levels were analysed across pAAb-defined serological groups and seronegative controls. pAAb stratification: a-SSA/Ro-dominant, a-dsDNA/a-nucleosome-dominant, and co-positive a-SSA/Ro with a-dsDNA. Panels depict the following analytes: **(A)** a-CS IgG, **(B)** a-CS IgM, **(C)** a-HSP60 IgG, **(D)** a-HSP60 IgM, **(E)** a-HSP70 IgG, and **(F)** a-HSP70 IgM. All diagnostically relevant pAAbs were of the IgG isotype (x-axis categories). The y-axis represents serum concentrations (AU/mL) of nAAbs (IgG or IgM, as indicated). Group sizes were as follows: NC (n = 39), a-SSA/Ro (n = 15), a-dsDNA/a-nucleosome (n = 14), co-positive (n = 14); a-dsDNA/a-nucleosome groups (a-dsDNA/a-Nuc in the image) include patients with a-dsDNA and/or a-nucleosome IgG positivity; groups were assigned after review of each patient’s complete diagnostic record (see Methods). The number of samples with a result for each analyte is shown below each group. Data are presented as raincloud plots showing individual data points, box plots (median and interquartile range; whiskers, 1.5 × IQR) and kernel density estimates. Statistical analysis was performed using Kruskal–Wallis tests followed by Dunn’s *post hoc* comparisons with Holm correction. Significant pairwise differences (Holm-adjusted) are indicated as follows: *p_Holm_ ≤ 0.05; **p_Holm_ ≤ 0.01; ***p_Holm_ ≤ 0.001; †p_Holm_ < 0.10.

**Figure 6:**
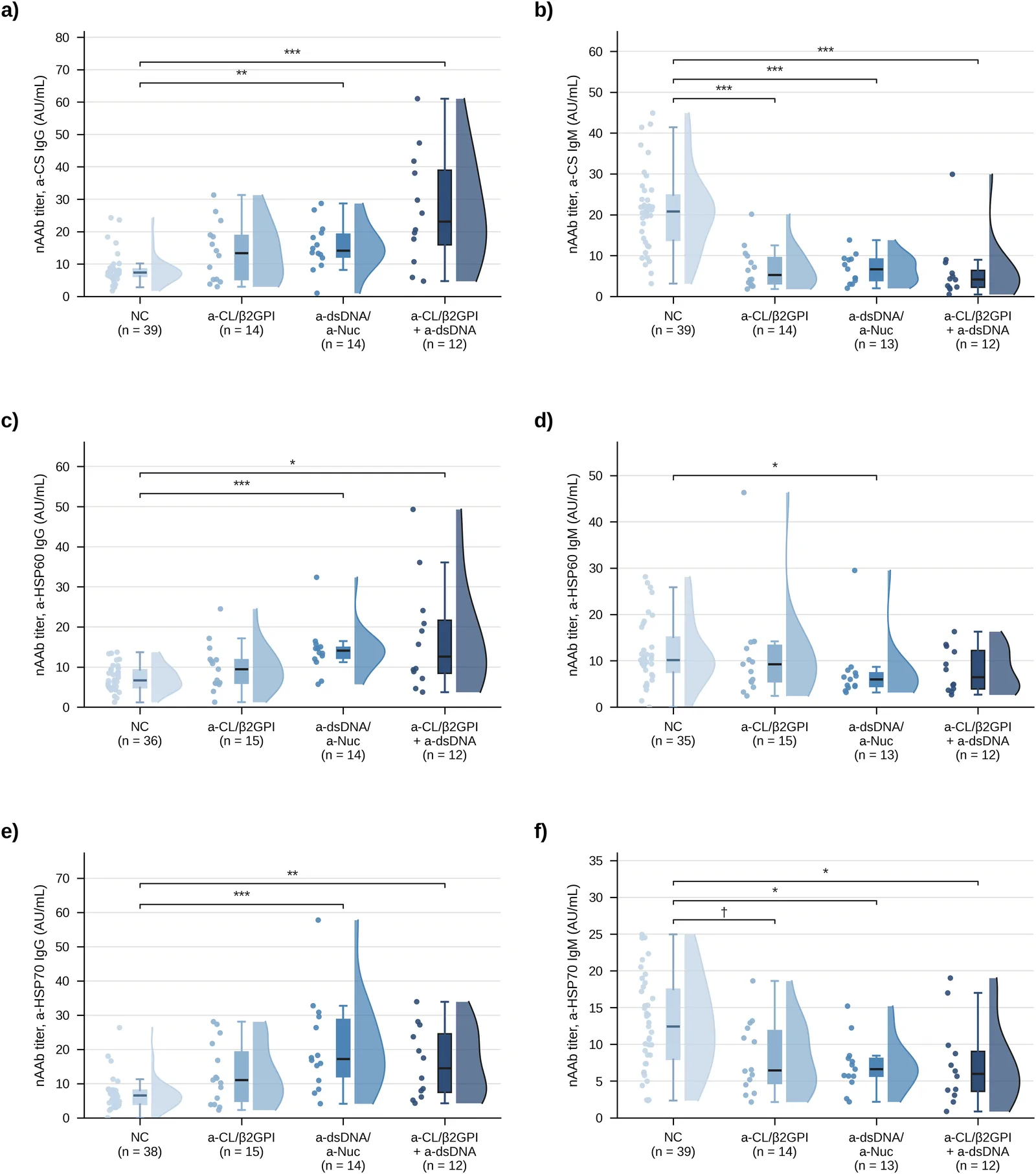
nAAb patterns across dominant and co-positive serological subgroups, with particular focus on the a-CL/β2GPI + a-dsDNA co-positive subgroup. Serum nAAb levels were analysed across pAAb-defined serological groups and seronegative controls. pAAb stratification: a-CL/β2GPI-dominant, a-dsDNA/a-nucleosome-dominant, and co-positive a-CL/β2GPI + a-dsDNA. Panels depict the following analytes: **(A)** a-CS IgG, **(B)** a-CS IgM, **(C)** a-HSP60 IgG, **(D)** a-HSP60 IgM, **(E)** a-HSP70 IgG, and **(F)** a-HSP70 IgM. The group-defining pAAbs (x-axis categories) were a-CL/β2GPI IgG and/or IgM in the a-CL/β2GPI-dominant group and IgG in the other groups. The y-axis represents serum concentrations (AU/mL) of nAAbs (IgG or IgM, as indicated). Group sizes were as follows: NC (n = 39), a-CL/β2GPI (n = 16), a-dsDNA/a-nucleosome (n = 14), co-positive (n = 12); a-dsDNA/a-nucleosome groups (a-dsDNA/a-Nuc in the image) include patients with a-dsDNA and/or a-nucleosome IgG positivity; groups were assigned after review of each patient’s complete diagnostic record (see Methods). The number of samples with a result for each analyte is shown below each group. Data are presented as raincloud plots showing individual data points, box plots (median and interquartile range; whiskers, 1.5 × IQR) and kernel density estimates. Statistical analysis was performed using Kruskal–Wallis tests followed by Dunn’s *post hoc* comparisons with Holm correction. Significant pairwise differences (Holm-adjusted) are indicated as follows: *p_Holm_ ≤ 0.05; **p_Holm_ ≤ 0.01; ***p_Holm_ ≤ 0.001; †p_Holm_ < 0.10.

## Results

### Testing/co-testing frequencies

When testing frequencies were assessed relative to the total number of test requests (n = 128,847), as an indicator of laboratory diagnostic burden, a clear hierarchical structure emerged. High-frequency testing (≥35%) was dominated by screening and core nuclear assays, including ANA screening (49.08%), ENA screening (39.50%), and a-nucleosome IgG (35.70%). An upper-intermediate cluster (30–35%) comprised a-dsDNA IgG (33.34%), aPL panel testing (34.88%), and ANCAs (34.36%). Intermediate-frequency testing (20–29.9%) included a-dsDNA with ENA panel co-testing (25.78%), a-prothrombin antibodies (24.23%), a-dsDNA with a-nucleosome co-testing (23.33%) and a-dsDNA with aPL co-testing (21.92%). Lower-intermediate co-testing frequencies (10–19.9%) were observed for dsDNA with ANCAs (16.81%) and dsDNA with a-prothrombin antibodies (14.23%), in alignment with downstream laboratory workflow. (The complete analysis is presented in **Supplementary Table S1**).

### Sero-reactivity hierarchies

#### Adjusted for the number of screened patients

For single-analyte testing, positivity ratios were calculated using the number of patients tested within the corresponding diagnostic panel as the denominator and were supported by exact 95% confidence intervals (CIs) (**Figure 1A**; **Supplementary Table S1**). Based on this CI-supported stratification, clearly separated high-frequency markers (≥5%) included ANA screening (17.35%, 95% CI: 16.99–17.71), a-nucleosome IgG (11.62%, 11.27–11.98), a-dsDNA IgG (9.02%, 8.69–9.37), and a-SSA/Ro IgG (5.83%, 5.53–6.15). These markers show non-overlapping CIs with all lower-frequency groups, supporting a distinct high-prevalence cluster. A second, intermediate-frequency cluster (1–4.99%) comprised a-CL/β2GPI IgM (4.62%, 4.39–4.86), a-CL/β2GPI IgG (2.81%, 2.63–3.00), a-PT IgG (2.77%, 2.56–2.99), a-SSB/La IgG (2.24%, 2.05–2.45), a-PT IgM (1.61%, 1.45–1.79), a-Jo-1 IgG (1.40%, 1.25–1.57), a-U1-RNP IgG (1.37%, 1.22–1.53), a-Sm IgG (1.20%, 1.06–1.35), a-PR3 IgG (c-ANCA) (1.18%, 1.06–1.30), and a-MPO IgG (p-ANCA) (1.00%, 0.89–1.11). Within this group, partial CI overlap is present, but the cluster remains clearly separated from the high-frequency markers. Only a-Scl-70 IgG fell below 1% (0.64%, 0.54–0.76).

Co-tested pAAb combinations displayed a compressed frequency distribution, requiring independent interpretation due to lower prevalence and stronger CI overlap (**Figure 1A**; **Supplementary Table S1**). The highest co-positivity group (≥1%) included a-dsDNA IgG with total ENA panel positivity (2.90%, 2.63–3.20), ENA subpanel (SLE/Sjögren’s/mixed connective tissue disease-associated) co-positivity (2.79%, 2.52–3.08), a-dsDNA IgG with a-SSA/Ro IgG (2.02%, 1.79–2.27), a-dsDNA IgG with a-CL/β2GPI IgG/M (2.01%, 1.81–2.22), a-dsDNA IgG with a-CL/β2GPI IgM (1.41%, 1.25–1.59), and a-dsDNA IgG with a-CL/β2GPI IgG (1.14%, 0.99–1.29). These combinations showed overlapping but consistently elevated CIs, distinguishing them from lower-frequency co-testing patterns. Co-positivity of a-dsDNA with a-nucleosome IgG, which targets the same antigen (chromatin), is shown separately (5.18%, 4.88–5.50; **Figure 1A**).

#### Sero-reactivity hierarchy adjusted for a-dsDNA IgG positivity

For the overall assessment of a-dsDNA IgG positivity–associated laboratory diagnostic poly-reactivity, our findings should be interpreted in the context of ELISA-based serology, rather than clinically confirmed SLE diagnoses. Nevertheless, the observed co-positivity frequency is broadly consistent with literature reports on clinically defined SLE cohorts, where poly-autoimmunity has been described in approximately 13–38% of cases (48–50). In our dataset, a comparable frequency was observed: 30.36% of a-dsDNA IgG–positive patients (743/2,447) showed co-positivity for at least one additional disease-specific pAAb (a-CL/β2GPI, ENA antigen or ANCA) in the same sample (32.65% when a-PT antibodies were also considered).

When calculating exact 95% CIs using the number of a-dsDNA IgG–positive patients as the denominator, a distinct high-frequency co-positivity cluster (≥10%) emerged (**Figure 1B**; **Supplementary Table S1**). This included ENA reactivity (16.51%, 95% CI: 15.06–18.04), a-CL/β2GPI IgG/M positivity (15.90%, 14.47–17.41), ENA subpanel (a-SSA/Ro, a-SSB/La, a-Sm, and/or a-U1-RNP IgG) reactivity (15.86%, 14.43–17.36), a-CL/β2GPI IgM (11.16%, 9.94–12.47), and a-SSA/Ro IgG (11.48%, 10.25–12.81). Despite overlapping CIs within this cluster, these markers remain clearly separated from lower-frequency groups, supporting a distinct high-frequency stratum. Chromatin co-reactivity with a-nucleosome IgG was present in 42.01% (40.05–44.00) of a-dsDNA IgG-positive patients; as both antibodies target the same antigen, this combination was not regarded as an additional specificity and is shown separately (**Figure 1B**).

A second, intermediate-frequency cluster (3–9.99%) comprised of a-CL/β2GPI IgG (8.99%, 7.89–10.19), a-PT IgG (4.50%, 3.71–5.39), a-U1-RNP IgG (4.58%, 3.78–5.48), a-SSB/La IgG (4.90%, 4.08–5.84), a-Sm IgG (3.47%, 2.78–4.28), and ANCAs (3.02%, 2.38–3.78). Within this range, moderate CI overlap is observed, whilst separation from the high-frequency cluster remains preserved.

A third, low-frequency cluster included groups below <3%, showing substantial CI overlap with the lower boundary of the intermediate group, indicating a gradual transition rather than a sharply separated stratum.

### Intersection patterns across pAAb specificities

To visualise the overlap structure of the ELISA-based pAAb specificities simultaneously, exclusive intersections were analysed using UpSet plots (**Figure 2**; **Supplementary Table S2**). Of the 73,747 patients, 7,721 (10.47%) were positive for at least one of the 11 displayed specificities. Seroreactivity was predominantly restricted to a single specificity (5,428 patients; 70.30%), whereas 2,293 patients (29.70%) showed two or more and 624 (8.08%) three or more specificities within the same sample (**Figure 2A**). The largest single-specificity groups were a-nucleosome IgG (n = 2,037), a-CL/β2GPI (n = 1,275) and a-dsDNA IgG (n = 1,052), followed by a-SSA/Ro (n = 394), a-PR3 (n = 293) and a-MPO (n = 184). The most frequent multi-specificity intersection was the chromatin pair of a-dsDNA and a-nucleosome IgG (n = 647), followed by a-SSA/Ro with a-SSB/La (n = 212), a-dsDNA with a-CL/β2GPI (n = 157) and a-nucleosome IgG with a-CL/β2GPI (n = 135). The tendency to occur in isolation differed markedly between specificities: a-PR3 (80.72%), a-CL/β2GPI (70.33%), a-MPO (62.59%) and a-nucleosome IgG (58.03%) were most often the only positive specificity, whereas a-Sm (4.70%), a-Scl-70 (11.76%), a-U1-RNP (12.36%), a-SSB/La (13.76%) and a-Jo-1 (24.65%) were mostly accompanied by other reactivities; a-dsDNA IgG occurred in isolation in 46.02% of positive patients.

Within the a-dsDNA IgG-positive subset (n = 2,447; **Figure 2B**), 1,704 patients (69.64%) had no a-dsDNA IgG-positive sample with an additional disease-specific reactivity, whereas 743 (30.36%) had at least one (a-nucleosome IgG, targeting the same antigen, was not counted as an additional specificity). In their first co-positive sample, 519 patients (21.21%) were positive for one additional specificity, 175 (7.15%) for two, and 49 (2.00%) for three or more. The most frequent combinations were a-dsDNA with a-CL/β2GPI (n = 301; 12.30%), with a-SSA/Ro (n = 113; 4.62%), with a-SSA/Ro and a-SSB/La (n = 69; 2.82%), and with a-Sm and a-U1-RNP (n = 31; 1.27%). The intersection structure thus reproduced the two axes identified in **Figure 1B**: an aPL axis (a-CL/β2GPI, n = 361; without concomitant ENA antigen or ANCA reactivity in 301 patients) and an ENA axis (any ENA antigen, n = 383; without aPL or ANCA reactivity in 324 patients), the latter dominated by a-SSA/Ro and its pairing with a-SSB/La (n = 90; 33.96% of a-SSA/Ro-positive patients) and by the a-Sm/a-U1-RNP pairing (n = 49). The two axes overlapped in only 53 patients (2.17%), whereas ANCA co-positivity remained rare (n = 66; 2.70%), with 12 patients co-positive for both a-PR3 and a-MPO. Because only one sample per patient enters this analysis, per-specificity totals are slightly lower than the analyte-by-analyte counts of **Figure 1B** (e.g., a-SSA/Ro 265 versus 281 patients).

To summarise these intersections at the level of the main serological axes, co-positive a-dsDNA IgG-positive patients (n = 743) were grouped into aPL (a-CL/β2GPI IgG/M), Ro/La (a-SSA/Ro and/or a-SSB/La) and Sm/U1-RNP (a-Sm and/or a-U1-RNP) reactivities, with the remaining specificities combined as “others” (**Figure 3**). Amongst double-positive patients (a-dsDNA IgG plus one additional antibody; n = 519), aPL reactivity predominated (n = 301; 58.0%), followed by Ro/La (n = 126; 24.3%) and Sm/U1-RNP (n = 36; 6.9%), whereas 56 patients (10.8%) belonged to the “others” group, mostly with ANCA (n = 43). Amongst triple- and higher-positive patients (n = 224), reactivity restricted to the Ro/La axis was the most frequent pattern (n = 82; 36.6%), followed by Sm/U1-RNP alone (n = 41; 18.3%). Overlaps between axes accounted for a further 82 patients (36.6%): aPL with Ro/La (n = 36; 16.1%), Ro/La with Sm/U1-RNP (n = 31; 13.8%), aPL with Sm/U1-RNP (n = 9; 4.0%) and all three axes (n = 6; 2.7%), whereas aPL without another main axis (n = 9; 4.0%) and “others” (n = 10; 4.5%) were rare in this group.

### Sex distribution of pAAb positivity

Women accounted for 48,429 of the 73,747 patients (65.67%; median age 53 years, IQR 39–67) and were further over-represented amongst patients tested within the autoimmune diagnostic panels (66.6–75.0%) and, particularly, amongst pAAb-positive patients (**Supplementary Figure S2A**; **Supplementary Table S3**). Women represented 78.7% of ANA screen-positive, 80.8% of a-dsDNA IgG-positive and 82.1% of a-nucleosome IgG-positive patients, and 81.1–89.3% of patients positive for a-SSA/Ro, a-SSB/La, a-Sm, a-U1-RNP or a-Scl-70 IgG. After adjustment for age, female sex remained associated with positivity for ANA screening (OR 1.75, 95% CI 1.64–1.86), a-dsDNA IgG (1.72, 1.55–1.91), a-nucleosome IgG (1.92, 1.76–2.10), a-SSA/Ro IgG (2.55, 2.16–3.01), a-SSB/La IgG (2.97, 2.23–3.96), a-U1-RNP IgG (1.93, 1.41–2.63), a-Sm IgG (1.53, 1.12–2.09), a-Scl-70 IgG (1.74, 1.12–2.70) and a-MPO IgG (1.81, 1.39–2.37), and, more weakly, with a-CL/β2GPI IgM (1.20, 1.06–1.36) (**Supplementary Figure S2B**). In contrast, a-CL/β2GPI IgG, a-PT IgG/IgM and a-Jo-1 IgG showed no significant sex difference, and a-PR3 IgG was more frequent in men (OR 0.64, 0.52–0.78). Amongst a-dsDNA IgG-positive patients, the proportion of women increased with the number of additional autoantibodies, from 78.5% in patients without additional reactivity to 85.0% in double-positive and 88.8% in triple- or higher-positive patients (p for trend < 0.001), whereas age did not differ significantly between these groups (Kruskal–Wallis p = 0.079; **Supplementary Figure S2C**).

### Selection of pAAb-defined serological clusters for downstream nAAb measurements

ANA positivity was used as a systemic autoreactivity entry criterion, whereas a-dsDNA IgG was selected as the principal SLE-associated stratification marker due to its higher diagnostic specificity (7, 51). Based on CI-supported stratification (frequency tiers I,II of **Supplementary Table S1**) and diagnostic interpretive value (38–42), a-dsDNA IgG, a-CL/β2GPI IgG and/or IgM, and a-SSA/Ro IgG were selected as single-specificity (dominant) groups, whilst a-dsDNA IgG with a-CL/β2GPI IgG and a-dsDNA IgG with a-SSA/Ro IgG were included as the most frequent co-positive combinations for subsequent nAAb measurements.

### Examining nAAb profiles aligned across the pAAb-hallmarked clusters

To characterise nAAb profiles across serological states, nAAb levels were evaluated in pAAb-stratified subgroups as summarised in **Figure 4**.

#### Anti–citrate synthase nAAbs

For a-CS IgG (**Figure 4A**), the a-dsDNA/a-nucleosome-dominant group exhibited significantly elevated levels compared to seronegative controls (NC) (p_Holm_ = 0.004), whereas the increases in the a-CL/β2GPI (p_Holm_ = 0.247) and a-SSA/Ro (p_Holm_ = 0.676) dominant groups were not significant. Median values increased from 7.41 AU/mL (IQR 6.15–8.45) in NC to 7.50 AU/mL (4.90–12.15) in a-SSA/Ro, 13.35 AU/mL (5.22–18.86) in a-CL/β2GPI and 14.15 AU/mL (12.15–19.24) in a-dsDNA/a-nucleosome. Amongst patient groups, a-CS IgG levels tended to be higher in the a-dsDNA/a-nucleosome than in the a-SSA/Ro group (p_Holm_ = 0.057).

In contrast, a-CS IgM (**Figure 4B**) nAAbs displayed a marked and uniform reduction across all dominant groups compared to NC (all p_Holm_ < 0.001). This was also reflected in median values, which decreased from 20.85 AU/mL (IQR 13.73–24.84) in NC to 3.95 AU/mL (2.33–7.53) in a-SSA/Ro, 5.30 AU/mL (3.10–9.50) in a-CL/β2GPI and 6.70 AU/mL (3.90–9.13) in a-dsDNA/a-nucleosome. No differences were observed between pAAb-positive subgroups.

#### Alignment with a-HSP60 and a-HSP70

The pattern observed for a-CS nAAbs was partially recapitulated by nAAbs directed against HSPs, particularly at the level of isotype distribution. For a-HSP60 IgG (**Figure 4C**), significantly elevated levels were observed in the a-dsDNA/a-nucleosome-dominant group compared to NC (p_Holm_ < 0.001), whereas differences in a-SSA/Ro and a-CL/β2GPI were not significant. No differences were observed between patient groups, indicating that IgG nAAb elevation is most pronounced in a-dsDNA–associated autoimmunity, consistent with the a-CS pattern.

At the IgM level, a-HSP60 nAAbs (**Figure 4D**) were significantly reduced in the a-SSA/Ro (p_Holm_ = 0.027) and a-dsDNA/a-nucleosome (p_Holm_ = 0.038) groups compared to NC, with no difference in the a-CL/β2GPI group (p_Holm_ = 0.651).

For a-HSP70 IgG (**Figure 4E**), the a-dsDNA/a-nucleosome-dominant group again exhibited markedly elevated levels compared to NC (p_Holm_ < 0.001), whereas the a-SSA/Ro and a-CL/β2GPI groups did not differ significantly from NC; a-HSP70 IgG levels were also higher in the a-dsDNA than in the a-SSA/Ro group (p_Holm_ = 0.010).

Finally, a-HSP70 IgM nAAbs (**Figure 4F**) were lower in all dominant groups than in NC, reaching significance in the a-dsDNA group (p_Holm_ = 0.024), with a trend in the a-SSA/Ro and a-CL/β2GPI groups (both p_Holm_ = 0.090) and no differences between patient groups.

### nAAb responses in a-dsDNA IgG with a-SSA/Ro IgG co-positivity

To assess nAAb profiles in the context of serological co-positivity and dsDNA-associated autoimmunity, nAAb responses were analysed across dominant and co-positive subgroups as summarised in **Figure 5**.

#### Anti–citrate synthase nAAbs

For a-CS IgG (**Figure 5A**), both the a-dsDNA/a-nucleosome-dominant and the a-SSA/Ro + a-dsDNA co-positive subgroup exhibited significantly elevated levels compared to NCs (p_Holm_ = 0.002 and p_Holm_ = 0.012, respectively), whereas the a-SSA/Ro-dominant group did not differ from NC (p_Holm_ = 1.000). a-CS IgG levels were higher in the a-dsDNA/a-nucleosome-dominant than in the a-SSA/Ro-dominant group (p_Holm_ = 0.042), whereas no difference was observed between the a-dsDNA/a-nucleosome-dominant and the co-positive subgroup (p_Holm_ = 1.000).

Median a-CS IgG levels were similar in the co-positive subgroup (13.25 AU/mL, IQR 8.32–26.28) and the a-dsDNA/a-nucleosome-dominant group (14.15 AU/mL, IQR 12.15–19.24), with the widest spread in the co-positive subgroup.

In contrast, a-CS IgM nAAbs (**Figure 5B**) showed a uniform and marked reduction across all pAAb-positive groups, including the co-positive group, compared to NC (all p_Holm_ < 0.001). No differences were detected between the dominant and co-positive groups.

#### Alignment with a-HSP60 and a-HSP70 nAAbs

The co-positivity-associated pattern observed for a-CS was partially recapitulated by nAAbs directed against a-HSP60 and a-HSP70. For a-HSP60 IgG (**Figure 5C**), the a-dsDNA/a-nucleosome-dominant group showed significantly higher levels compared to NC (p_Holm_ = 0.001), whilst the co-positive subgroup demonstrated only a non-significant increase (p_Holm_ = 0.223). No differences were observed between the a-dsDNA/a-nucleosome-dominant and co-positive groups, paralleling the a-CS IgG pattern.

At the IgM level, a-HSP60 mirrored the a-CS profile (**Figure 5D**), with significantly higher levels in NC than in the a-SSA/Ro-dominant (p_Holm_ = 0.022) and co-positive (p_Holm_ < 0.001) groups as well as in the a-dsDNA/a-nucleosome-dominant group (p_Holm_ = 0.042), and no differences between patient subsets.

For a-HSP70 IgG (**Figure 5E**), a strong increase was again observed in the a-dsDNA/a-nucleosome-dominant group compared to NC (p_Holm_ < 0.001), whereas the co-positive subgroup did not differ from NC (p_Holm_ = 0.878). a-HSP70 IgG levels in the a-dsDNA/a-nucleosome-dominant group were also higher than in the a-SSA/Ro-dominant group (p_Holm_ = 0.007), with a trend towards higher levels than in the co-positive group (p_Holm_ = 0.051).

Finally, a-HSP70 IgM (**Figure 5F**) followed a similar directionality to a-CS IgM, with reduced levels in pAAb-positive groups compared to NC, reaching significance in the a-dsDNA/a-nucleosome-dominant group (p_Holm_ = 0.015), with a trend in the a-SSA/Ro-dominant and co-positive groups (both p_Holm_ = 0.069). As with a-CS IgM and a-HSP60 IgM, no differences were detected between dominant and co-positive groups.

### nAAb responses in a-dsDNA IgG with a-CL/β2GPI IgG co-positivity

To evaluate nAAb profiles in the context of aPL-associated and dsDNA-related co-positivity, nAAb responses were analysed across dominant and co-positive subgroups as summarised in **Figure 6**.

#### Anti–citrate synthase nAAbs

For a-CS IgG (**Figure 6A**), the a-dsDNA/a-nucleosome-dominant (p_Holm_ = 0.009) and, particularly, the co-positive a-CL/β2GPI + a-dsDNA subgroup (p_Holm_ < 0.001) showed significantly elevated levels compared to NC, whereas the increase in the a-CL/β2GPI-dominant group was not significant (p_Holm_ = 0.248). The co-positive subgroup demonstrated the highest median a-CS IgG levels (23.13 AU/mL, IQR 15.95–38.98), exceeding those of both dominant groups (13.35 and 14.15 AU/mL for a-CL/β2GPI and a-dsDNA, respectively).

Despite this marked quantitative increase, differences between patient subgroups were not significant.

In contrast, a-CS IgM (**Figure 6B**) displayed a uniform and profound reduction across all pAAb-positive groups, including the co-positive group, compared to NC (all p_Holm_ < 0.001). No differences were observed between dominant and co-positive groups.

#### Alignment with a-HSP60 and a-HSP70 nAAbs

The pattern observed for a-CS was partially recapitulated by nAAbs directed against a-HSP60 and a-HSP70. For a-HSP60 IgG (**Figure 6C**), significantly elevated levels were observed in both the a-dsDNA/a-nucleosome-dominant and co-positive subgroups compared to NC (p_Holm_ < 0.001 and p_Holm_ = 0.014, respectively), with similar median values in both groups (14.09 and 12.64 AU/mL). Differences between patient groups did not reach statistical significance, paralleling the a-CS IgG pattern.

At the IgM level, a-HSP60 (**Figure 6D**) showed a less uniform pattern than a-CS: with an overall group difference (Kruskal–Wallis p = 0.036), levels were significantly lower only in the a-dsDNA/a-nucleosome-dominant group (p_Holm_ = 0.039), whilst the a-CL/β2GPI-dominant and co-positive subgroups did not differ from NC.

For a-HSP70 IgG (**Figure 6E**), the a-dsDNA/a-nucleosome-dominant and co-positive groups demonstrated significantly higher levels compared to NC (p_Holm_ < 0.001 and p_Holm_ = 0.010, respectively), again with no differences between patient subgroups.

Finally, a-HSP70 IgM (**Figure 6F**) showed a moderate decrease in the a-dsDNA and co-positive groups (both p_Holm_ = 0.028), whilst changes in the a-CL/β2GPI-dominant group did not reach significance. As with a-CS, no differences were observed between patient subgroups.

### Age and sex of the nAAb sub-study groups

All 39 seronegative controls were women (median age 45 years, IQR 36–47), whereas the proportion of women in the pAAb-positive groups ranged from 75.0% (a-CL/β2GPI) to 100% (a-dsDNA), and the a-SSA/Ro- and a-CL/β2GPI-dominant groups were older than the controls (median 57 and 51.5 vs 45 years, p = 0.006 and p = 0.003; **Supplementary Table S4**). Amongst controls, nAAb levels were not associated with age, except for a weak inverse correlation of a-HSP70 IgM (Spearman ρ = −0.34, p = 0.033). The principal findings were robust in all three sensitivity analyses (women only, one sample per patient, and age- and sex-adjusted rank-based analysis of covariance). Across the 54 comparisons with seronegative controls, statistical significance was unchanged in 94–98% of cases, and the direction of every difference significant in both analyses was preserved. In particular, the reduction of a-CS IgM in all pAAb-positive groups, and the increase of all three IgG nAAbs together with the decrease of a-CS and a-HSP70 IgM in the a-dsDNA group, remained significant in every analysis. Changes were confined to borderline comparisons: after adjustment for age and sex, the trends towards lower a-HSP70 IgM in the a-SSA/Ro-dominant and co-positive groups (p_Holm_ = 0.069 vs 0.050) and towards higher a-HSP70 IgG in the a-CL/β2GPI-dominant group of **Figure 6** (p_Holm_ = 0.108 vs 0.023) reached significance, whereas in the a-CL/β2GPI with a-dsDNA group a-HSP60 IgG and a-HSP70 IgM did not remain significant when restricted to women (p_Holm_ = 0.330 and 0.061), and a-HSP70 IgM in this group also not with one sample per patient (p_Holm_ = 0.056) (**Supplementary Table S4**). Adjustment for the year of measurement did not change the findings: no significant difference was lost, the year of measurement was not associated with nAAb levels in any comparison (all p > 0.11), and in the model including year, age and sex, statistical significance agreed with the age- and sex-adjusted model in 96% of comparisons; in particular, the reduction of a-CS IgM in all pAAb-positive groups and the increase of all three IgG nAAbs in the a-dsDNA group remained significant after adjustment for year (**Supplementary Table S4**). The sensitivity power analysis showed that, with 80% power, comparisons with the 39 controls could detect effects of P(X > Y) ≥ 0.74–0.83 (Cohen’s d ≈ 0.9–1.4), whereas comparisons between two pAAb-positive groups of 12–16 samples could detect only effects of P(X > Y) ≥ 0.80–0.90 (d ≈ 1.2–1.8). The principal effects exceeded these thresholds: a-CS IgM was lower than in controls in all pAAb-positive groups [P(NC > group) = 0.90–0.95], and a-CS, a-HSP60 and a-HSP70 IgG were higher in the a-dsDNA group [P(group > NC) = 0.84–0.89]. Amongst the specificities that were not exclusion criteria, a-SSB/La was the most frequent additional positivity (8 of 15 patients in the a-SSA/Ro and 11 of 14 in the a-SSA/Ro with a-dsDNA group, as expected for Ro/La co-reactivity), whereas ENA antigens had been tested in only 2 of 16 patients of the a-CL/β2GPI and 5 of 14 patients of the a-dsDNA group, and recurrent a-Sm and/or a-U1-RNP positivity was present in 2 of 14 a-dsDNA/a-nucleosome-dominant, 2 of 14 a-SSA/Ro with a-dsDNA and 1 of 12 a-CL/β2GPI with a-dsDNA patients (**Supplementary Table S4**).

## Discussion

The study workflow, from the diagnostic cohort to the nAAb sub-study, and the supplementary analyses supporting each step are summarised in **Supplementary Figure S3**.

### Serological landscape of a-dsDNA-positive samples

#### Extent of co-positivity

In line with previous reports (48, 49), approximately 30% of a-dsDNA IgG–positive patients in our cohort showed additional diagnostically relevant pAAb co-positivity. Nevertheless, this frequency may underestimate the true extent of serological polyreactivity, as secondary laboratory testing was guided by clinical suspicion of an additional autoimmune manifestation. This approach may fail to capture clinically asymptomatic autoreactivities. Accordingly, a fully unbiased evaluation would require systematic extended pAAb screening, reflecting the intrinsically multilayered loss of immune tolerance in SLE (2–5, 8–10, 24, 25).

#### Two major serological axes: aPL and SSA/Ro

a-dsDNA-associated expanded autoreactive profiles likely reflect a mature, class-switched humoral response sustained by persistent germinal centre activity, aberrant T-cell help, and impaired B-cell tolerance checkpoints (1–3, 6, 8–10, 13, 24). Consistently, broad autoreactivity has been linked, in clinically characterised cohorts, to more established disease phenotypes and greater systemic involvement (4, 5, 12). In our serologically characterised cohort, a-dsDNA-associated co-positivity followed a structured pattern dominated by two major serological axes. The first was an aPL-associated axis, mainly characterised by co-reactivity with a-CL/β2GPI IgM and IgG, underscoring the diagnostic relevance of antiphospholipid syndrome (APS)-related autoreactivity within the a-dsDNA-positive landscape. This aligns with previous evidence that SLE patients frequently exhibit aPL positivity, whereas patients with primary APS may show ANA and a-dsDNA/chromatin reactivity even without clinical SLE. Importantly, primary APS rarely progresses to full-spectrum SLE, suggesting that such co-positivity reflects immunological proximity rather than true diagnostic overlap (10, 52, 53). The second was an ENA-associated axis, primarily driven by a-SSA/Ro IgG, with lower-frequency contribution from a-SSB/La IgG, indicating an SSA/Ro-centred SLE/Sjögren-like serological pattern. This interpretation is consistent with earlier studies defining aPL-dominant versus a-SSA/Ro- or a-SSB/La-dominant autoantibody subsets, conceptually supporting our serology-based clustering strategy (10, 54). Despite substantial parallel testing, ANCA-associated co-positivity remained rare, consistent with the low frequency but high clinical relevance of lupus vasculitis in SLE (11). Overall, these frequency tiers support a model of multilayered immune dysregulation and broad B-cell activation in a-dsDNA-directed seroreactivity (1–6, 8–10, 13, 24).

#### Combinatorial structure and chromatin co-reactivity

The UpSet and Venn analyses (**Figures 2**, **3**) add a combinatorial dimension to these frequency tiers. At the population level, about seven in ten pAAb-positive patients carried a single specificity; apart from the chromatin pair of a-dsDNA and a-nucleosome IgG, multi-specificity profiles clustered around ENA antigen reactivities, whereas a-PR3, a-MPO and a-CL/β2GPI positivity occurred predominantly in isolation, in line with ANCA-associated vasculitis and APS representing largely distinct serological entities (55–57). Within the a-dsDNA IgG-positive subset, approximately seven in ten patients showed no further disease-specific reactivity. Amongst the remaining patients, the exclusive intersections showed that the aPL- and ENA-associated axes were largely separate, overlapping in only a small minority of patients, and that higher-order combinations of three or more additional specificities were rare. Grouping by serological axis further showed that aPL reactivity was typically the only additional antibody in double-positive patients, whereas Ro/La- and Sm/U1-RNP-centred profiles and their overlaps predominated amongst patients with two or more additional antibodies. These patterns remain subject to test-ordering bias: antigen-specific ENA and ANCA assays were requested in only a subset of patients, typically following a positive screening result or clinical suspicion, so intersections involving these specificities are likely underestimated. Nevertheless, the separation of aPL-dominant and SSA/Ro-dominant a-dsDNA IgG-positive profiles supports the selection of the a-dsDNA with a-CL/β2GPI and a-dsDNA with a-SSA/Ro co-positive clusters for downstream nAAb profiling. Chromatin co-reactivity deserves separate mention: a-nucleosome IgG had the highest positivity ratio after the ANA screen (11.62% of tested patients; **Figure 1A**) and accompanied a-dsDNA IgG in 42.01% of a-dsDNA IgG-positive patients (**Figure 1B**), forming the most frequent combination in the population-level UpSet plot. As a-dsDNA and a-nucleosome antibodies target components of the same antigen complex, and a-nucleosome antibodies may precede or replace a-dsDNA positivity (37, 45–47), this overlap was treated as one chromatin-directed reactivity rather than as an additional specificity, and it formed the basis of the a-dsDNA/a-nucleosome groups of the nAAb sub-study.

#### Sex distribution of pAAb positivity

Consistent with the well-established female predominance of SLE and Sjögren’s disease (58), women were markedly over-represented amongst pAAb-positive patients, and this association was independent of age. The strongest female predominance was observed for a-SSA/Ro and a-SSB/La, whereas a-PR3 positivity was more frequent in men, in line with the epidemiology of PR3-ANCA-associated vasculitis (55), and aPL IgG positivity showed no sex difference, supporting the internal validity of this laboratory-based cohort. Moreover, the increasing proportion of women with the number of co-occurring autoantibodies amongst a-dsDNA IgG-positive patients suggests that female sex is associated not only with the presence but also with the breadth of SLE-associated autoreactivity.

### nAAb profiles across pAAb-defined serological groups

#### Rationale and novelty of the nAAb sub-study

Building on earlier work and literature data (2, 4, 5, 13, 14, 16, 24, 25, 28–30), we hypothesised that in a-dsDNA-dominant samples, where defective clearance, dysregulated cytokine signalling, and broad B-cell activation converge pAAb expansion may be accompanied by measurable nAAb network remodelling. Earlier clinical studies showed subgroup-restricted natural IgM depression in SLE, whilst IgM nAAbs have been implicated in atheroprotection, C1q recruitment, apoptotic-cell phagocytosis, and protection against plaque instability and related vascular risk (13, 14, 28–30). Compared with earlier studies (13, 14, 28–30) our presented sub-study adds a distinct perspective by using a broad laboratory-diagnostic cohort for sample selection, focusing on diagnostically relevant a-dsDNA IgG mono- and polyreactive pAAb patterns, and assessing nAAb alterations in an isotype-resolved framework. A distinctive feature of the study is thus the integration of the pathological and natural autoreactive compartments within a diagnostically anchored, serology-centred framework.

#### IgM nAAb reduction across pAAb-positive groups

Consistent with earlier studies (13, 14), our autoimmune diagnostic-serology-based analysis demonstrated reduced IgM nAAb levels across pAAb-positive serogroups, supporting weakened homeostatic IgM responses (14, 16, 29). Importantly, in contrast to earlier observations (13), this reduction was not restricted to a-Ro/La-like profiles but extended across several pAAb-positive diagnostic serogroups. This broader pattern suggests a more generalised impairment of protective homeostatic autoreactivity in pAAb-positive states, independent of the dominant autoantibody specificity (2, 3, 24).

#### IgG nAAb increase in a-dsDNA/a-nucleosome-containing profiles

In parallel, IgG-type nAAbs were increased in targeted high-autoreactivity profiles; this increase was confined to a-dsDNA/a-nucleosome-containing, i.e. chromatin-reactive, profiles (a-dsDNA/a-nucleosome-dominant and co-positive groups) and was absent in a-SSA/Ro- or a-CL/β2GPI-dominant profiles. This counter-directional IgM↓/IgG↑ pattern was observed across CS, HSP60, and HSP70 targets, most consistently in the a-dsDNA/a-nucleosome-dominant group, consistent with the hypothesis of coordinated remodelling of the nAAb network rather than an isolated antigen-specific phenomenon. A major statistically supported distinction was between seronegative and pAAb-positive serological profiles, whereas the apparent stepwise increase between dominant and co-positive clusters remained a non-significant trend and was not observed in the a-SSA/Ro with a-dsDNA co-positive subgroup, in which a-HSP70 IgG levels tended to be lower than in the a-dsDNA/a-nucleosome-dominant group. The IgG shift is consistent with class-switched B-cell activation but does not distinguish the underlying mechanism. Although IgG nAAbs may exert protective functions (18, 33), their selective expansion could also reflect progressive loss of self-tolerance and transition toward class-switched autoreactivity. Alternatively, these shifts might represent compensatory responses counteracting autoimmune pathology (27, 32, 59, 60). However, without functional assays (e.g., of apoptotic-cell clearance or pro-inflammatory activity), these interpretations remain speculative, and the IgG increase should not be taken as evidence of a protective or compensatory mechanism. Functional studies could address these hypotheses.

#### Strengths and limitations

The main strengths of the study are the size and duration of the diagnostic cohort (73,747 patients, 2014–2024), with uniform processing in an accredited laboratory; the selection of the nAAb sub-study groups from the observed co-positivity hierarchy of this cohort rather than from clinical diagnoses, with verification of every group assignment against up to ten years of serological follow-up of the patient; and the paired, isotype-resolved (IgG and IgM) assessment of three nAAb targets, which revealed counter-directional isotype shifts that IgM-centred studies could not capture. To our knowledge, isotype-divergent nAAb changes have not previously been examined across serologically defined, diagnostically anchored a-dsDNA-, a-SSA/Ro- and aPL-associated groups.

The nAAb sub-study was retrospective and should be regarded as hypothesis-generating. After patient-level verification of the serological group assignment, group sizes were small (12–16 samples per pAAb-positive group), which limits the statistical power for differences between pAAb-positive groups, for which only large effects [P(X > Y) ≥ 0.80–0.90] were detectable; the absence of significant differences between dominant and co-positive groups should therefore not be interpreted as evidence of equivalence. All seronegative controls were women and were not formally age-matched; however, the IgM↓/IgG↑ pattern persisted in women-only, one-sample-per-patient and age- and sex-adjusted analyses (**Supplementary Table S4**), although confirmation in prospectively age- and sex-matched cohorts is warranted. These findings require confirmation in larger, prospective and clinically characterised cohorts. Moreover, under the current ethical approval only retrospective serological data of pseudonymised patients could be analysed; clinical diagnoses and follow-up visits were therefore not available, and repeated diagnostic testing (serological follow-up, 2014–2024) was the only longitudinal information used. In addition, a-dsDNA and other SLE-associated pAAbs are not specific to SLE and also occur in other autoimmune, infectious and drug-induced conditions (61); the serological groups therefore cannot be equated with clinically defined SLE. Likewise, the dominant groups were defined by the three group-defining specificities and were not exclusively single-positive: other reactivities, most often a-SSB/La in the a-SSA/Ro groups, were present in some patients, and ENA antigens had been tested in only a minority of the a-CL/β2GPI- and a-dsDNA/a-nucleosome-dominant patients, so untested reactivities cannot be excluded (**Supplementary Table S4**). Furthermore, differences in IgM degradation due to extended (4–5 months) storage of individual samples cannot be fully ruled out as contributing to the observed reduction in IgM nAAb levels, as IgM stability under these storage conditions was not specifically validated. However, all sera, including those of the controls, were handled identically (storage at −20 °C within the 4–5-month post-analytical window, maximum two freeze–thaw cycles), nAAb levels were not associated with the year of measurement, and the IgM reduction was not uniform across antigens and groups (e.g., a-HSP60 IgM in the a-CL/β2GPI group was comparable to controls), arguing against a global storage-related artefact.

## Conclusions

Our study provides an isotype-resolved sero-diagnostic perspective on nAAb alterations in SLE-associated serological groups selected from a large, decade-long regional SARD diagnostic cohort. The main findings and their interpretation are summarised in **Figure 7**. In the diagnostic cohort, approximately 30% of a-dsDNA IgG-positive patients carried an additional pAAb, organised along two largely separate axes (aPL and a-SSA/Ro), and a-dsDNA overlapped with a-nucleosome reactivity in 42%. In the nAAb sub-study, IgM nAAbs were reduced in every pAAb-positive group irrespective of the dominant specificity, whereas IgG nAAbs were increased only in a-dsDNA/a-nucleosome-containing (chromatin-reactive) profiles. These findings support the hypothesis of immune-homeostatic dysregulation in pAAb-positive serology, with impaired homeostatic IgM-mediated regulation (IgM↓) and an additional class-switched IgG component in chromatin-reactive profiles (IgG↑), and suggest that extended screening strategies integrating disease-specific pAAb profiles with isotype-specific nAAb assessment may be of value. As the nAAb sub-study was hypothesis-generating, future studies would benefit from clinically characterised sample sets with larger numbers and less heterogeneity in potentially confounding factors. Key open questions are the clinical meaning of these shifts (diagnoses, disease activity and organ involvement), the function of the elevated IgG nAAbs, and their time course relative to pAAb expansion.

**Figure 7:**
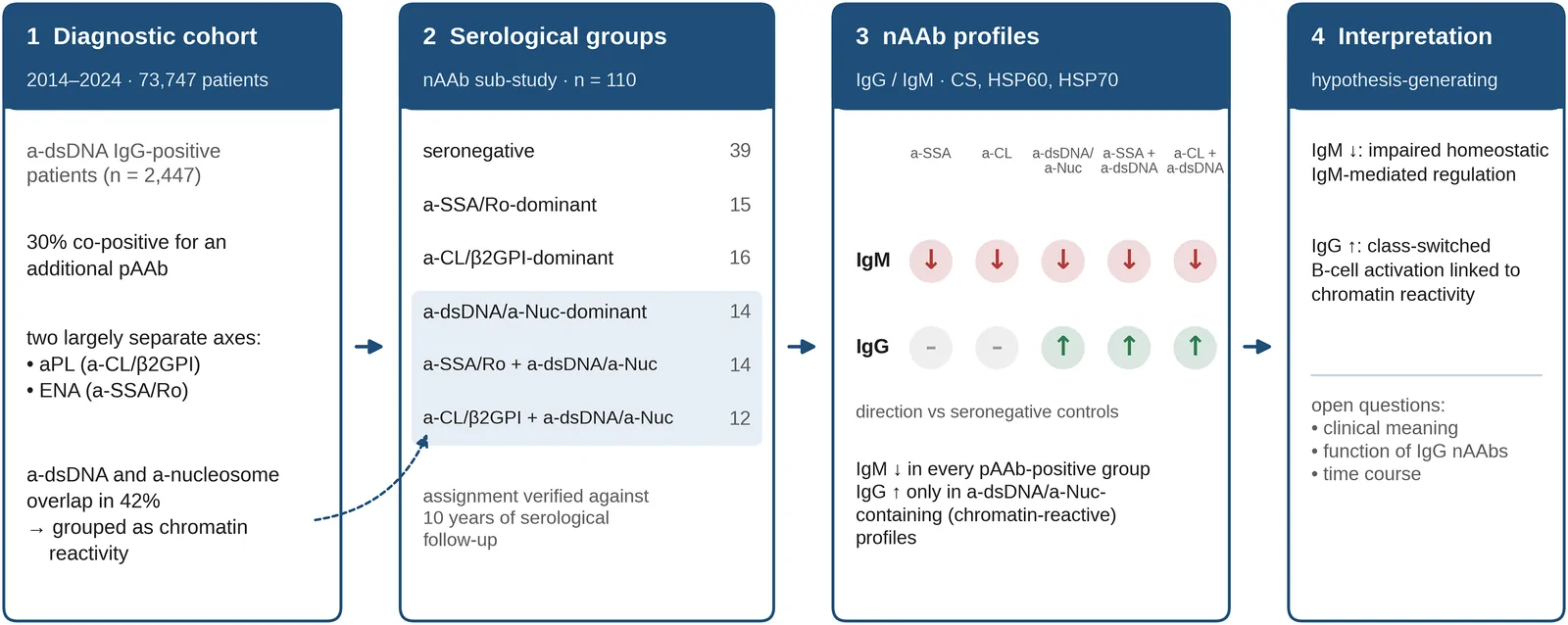
Summary of the study. (1) In the regional diagnostic cohort (2014–2024; 73,747 patients), 30% of a-dsDNA IgG-positive patients were co-positive for an additional pAAb, organised along two largely separate axes (aPL and a-SSA/Ro), and a-dsDNA and a-nucleosome IgG overlapped in 42% of them and were therefore grouped as chromatin reactivity (**Figures 1**–**3**; dashed arrow). (2) These dominant patterns defined the serological groups of the nAAb sub-study (n = 110; numbers of samples shown; shaded, groups containing chromatin reactivity), each assignment verified against the serological follow-up of the patient. (3) Direction of nAAb changes compared with seronegative controls, summarising **Figures 4**–**6** (mainly a-CS): ↓, lower; ↑, higher; –, no consistent difference. IgM nAAbs were reduced in every pAAb-positive group, whereas IgG nAAbs were increased only in a-dsDNA/a-nucleosome-containing profiles. (4) Hypothesis-generating interpretation and open questions. aPL, antiphospholipid antibodies; a-CL/β2GPI, anti-cardiolipin/anti-β2-glycoprotein I; CS, citrate synthase; HSP, heat shock protein; a-Nuc, anti-nucleosome; in panel 3, a-SSA, a-CL and a-dsDNA/a-Nuc denote the dominant groups, and a-SSA + a-dsDNA and a-CL + a-dsDNA the co-positive groups.

## Data Availability

The datasets presented in this article are not readily available because they contain de-identified human routine diagnostic data subject to institutional and ethical restrictions. Requests to access the datasets should be directed to the corresponding author.

## Supplementary material

Supplementary Figure S1 Longitudinal diagnostic pAAb results (2014–2024) of the nAAb sub-study patients, used to verify the serological group assignment. Each column represents one patient (pseudonymised code) with the final nAAb group(s); each dot is one measurement. Only patients positive for the respective marker(s) on at least two different dates are shown. **(A)** a-dsDNA IgG (cut-off 20 U/mL; values > 200 U/mL shown as 200). **(B)** a-CL IgG and a-β2GPI IgG (cut-offs 10 and 8 U/mL). **(C)** a-CL IgM and a-β2GPI IgM (cut-offs 7 and 8 U/mL). **(D)** a-PT IgG and IgM (cut-off 10 U/mL). **(E)** a-SSA/Ro and a-SSB/La (cut-offs 15 U/mL for the 2014–2020 and 25 U/mL for the 2020– assays). In **(B–E)**, values > 100 U/mL are shown as 100; dashed and dotted lines mark the cut-offs. Group codes: SSA, aCL and dsDNA, a-SSA/Ro-, a-CL/β2GPI- and a-dsDNA/a-nucleosome-dominant groups; SSA+dsDNA and aCL+dsDNA, co-positive groups; NC, seronegative controls.

Supplementary Figure S2 Sex distribution of pAAb positivity (patients aged 18–80 years, n = 73,747; women 65.67%). **(A)** Proportion of women amongst patients tested within the corresponding diagnostic panel (grey) and amongst positive patients (blue; exact 95% Clopper–Pearson CI); the dashed line marks the proportion of women in the whole cohort. **(B)** Odds ratios for positivity in women versus men, adjusted for age by Mantel–Haenszel stratification (18–39, 40–59, 60–80 years), with 95% CIs (log scale); blue and orange mark significant associations favouring women and men, respectively (p < 0.05), grey marks non-significant ones. **(C)** Proportion of women amongst a-dsDNA IgG-positive patients by the number of additional autoantibodies in the first co-positive sample (as in **Figure 2B**), with exact 95% CIs; the dashed line marks the cohort proportion; p for trend from the Cochran–Armitage test. Positivity definitions and denominators as in **Figure 1A**; see **Supplementary Table S3**.

Supplementary Figure S3 Study workflow. The main analytical steps (left, numbered) lead from the retrospective diagnostic cohort (tier 1) to the hypothesis-generating nAAb sub-study (tier 2) and its principal findings; the corresponding main figures are indicated in each box, and the supplementary figures and tables supporting each step are shown on the right.

Supplementary Table S1 Laboratory-validated ELISA cut-offs, testing frequencies, numbers of tested and co-tested patients (denominators), and pAAb positivity and co-positivity ratios with exact 95% Clopper–Pearson confidence intervals and frequency tiers underlying **Figure 1**.

Supplementary Table S2 Complete intersection (UpSet) and Venn diagram data underlying **Figures 2** and **3**.

Supplementary Table S3 Sex distribution of the cohort and of pAAb-positive patients, with crude and age-adjusted odds ratios for positivity in women versus men, and sex and age of a-dsDNA IgG-positive patients by the number of additional autoantibodies.

Supplementary Table S4 Age and sex of the nAAb sub-study groups, and sensitivity analyses (women only, one sample per patient, and age- and sex-adjusted rank-based analysis of covariance) of all comparisons with seronegative controls. Additional sheet: adjustment for the year of measurement. Additional sheets: valid and missing sample numbers per nAAb analyte, post-hoc sensitivity power analysis, and other pAAb specificities in each group.

## References

[1] Iwata S Tanaka Y . B-cell subsets, signaling and their roles in secretion of autoantibodies. Lupus. (2016) 25:850–6. doi: 10.1177/0961203316643172 27252261

[2] Bonasia CG Abdulahad WH Rutgers A Heeringa P Bos NA . B cell activation and escape of tolerance checkpoints: Recent insights from studying autoreactive B cells. Cells. (2021) 10:1190. doi: 10.3390/cells10051190 34068035 PMC8152463

[3] Deguine J Xavier RJ . B cell tolerance and autoimmunity: Lessons from repertoires. J Exp Med. (2024) 221:e20231314. doi: 10.1084/jem.20231314 39093312 PMC11296956

[4] Song Y Li J Wu Y . Evolving understanding of autoimmune mechanisms and new therapeutic strategies of autoimmune disorders. Signal Transduct Target Ther. (2024) 9:263. doi: 10.1038/s41392-024-01952-8 39362875 PMC11452214

[5] Parentelli A-S Lopes A-A Rieux-Laucat F Hermine O . Systemic auto-inflammatory diseases, auto-immune diseases and immune deficiencies: From independent to overlapped diseases approach. Autoimmun Rev. (2025) 24:103901. doi: 10.1016/j.autrev.2025.103901 40769404

[6] Salem D Subang R Okazaki Y Laplante P Levine JS Kuwana M . β2-Glycoprotein I-specific T cells are associated with epitope spread to lupus-related autoantibodies. J Biol Chem. (2015) 290:5543–54. doi: 10.1074/jbc.M114.619817 25555913 PMC4342469

[7] Aringer M Costenbader K Daikh D Brinks R Mosca M Ramsey-Goldman R . 2019 European League Against Rheumatism/American College of Rheumatology classification criteria for systemic lupus erythematosus. Arthritis Rheumatol. (2019) 71:1400–12. doi: 10.1002/art.40930 31385462 PMC6827566

[8] Tariq F Charavanmuttu Y Labiba K Wincup C . Aberrant B cell responses as drivers of autoantibody generation and epitope diversification in SLE pathogenesis. Front Immunol. (2026) 16:1731285. doi: 10.3389/fimmu.2025.1731285 41583477 PMC12827690

[9] Fulvio G La Rocca G Tani C Mosca M Baldini C . Sjögren’s disease and systemic lupus erythematosus overlap: Immunological insights and therapeutic implications. Front Lupus. (2025) 3:1600768. doi: 10.3389/flupu.2025.1600768

[10] Richter P Badescu MC Rezus C Ouatu A Dima N Popescu D . Antiphospholipid antibodies as key players in systemic lupus erythematosus: The relationship with cytokines and immune dysregulation. Int J Mol Sci. (2024) 25:11281. doi: 10.3390/ijms252011281 39457063 PMC11509045

[11] Calle-Botero E Abril A . Lupus vasculitis. Curr Rheumatol Rep. (2020) 22:71. doi: 10.1007/s11926-020-00937-0 32845454

[12] Shah S Chengappa KG Negi VS . Systemic lupus erythematosus and overlap: A clinician perspective. Clin Dermatol Rev. (2019) 3:12. doi: 10.4103/CDR.CDR_44_18

[13] Grönwall C Hardt U Gustafsson JT Elvin K Jensen-Urstad K Kvarnström M . Depressed serum IgM levels in SLE are restricted to defined subgroups. Clin Immunol. (2017) 183:304–15. doi: 10.1016/j.clim.2017.09.013 28919518

[14] Grönwall C Clancy RM Getu L Lloyd KA Siegel DL Reed JH . Modulation of natural IgM autoantibodies to oxidative stress-related neo-epitopes on apoptotic cells in newborns of mothers with anti-Ro autoimmunity. J Autoimmun. (2016) 73:30–41. doi: 10.1016/j.jaut.2016.05.014 27289167 PMC5003717

[15] Hurez V Kaveri S-V Kazatchkine MD . Expression and control of the natural autoreactive IgG repertoire in normal human serum. Eur J Immunol. (1993) 23:783–9. doi: 10.1002/eji.1830230402 8458369

[16] Böröcz K Szinger D Simon D Berki T Németh P . Regulators and conductors of immunity: Natural immune system in health and autoimmunity. Int J Mol Sci. (2025) 26:5413. doi: 10.3390/ijms26115413 40508221 PMC12155508

[17] Baumgarth N . B-1 cell heterogeneity and the regulation of natural and antigen-induced IgM production. Front Immunol. (2016) 7:324. doi: 10.3389/fimmu.2016.00324 27667991 PMC5016532

[18] Danieli MG Claudi I Buti E Gammeri L Gangemi S Shoenfeld YJ . Natural autoantibodies and their functional therapeutic roles in intravenous immunoglobulin. Front Aging. (2025) 6:1682457. doi: 10.3389/fragi.2025.1682457 41103507 PMC12522042

[19] Czömpöly T Olasz K Simon D Nyárády Z Pálinkás L Czirják L . A possible new bridge between innate and adaptive immunity: Are the anti-mitochondrial citrate synthase autoantibodies components of the natural antibody network? Mol Immunol. (2006) 43:1761–8. doi: 10.1016/j.molimm.2005.11.004 16368144

[20] Varbiro S Biro A Cervenak J Cervenak L Singh M Banhidy F . Human anti-60 kD heat shock protein autoantibodies are characterized by basic features of natural autoantibodies. Acta Physiol Hung. (2010) 97:1–10. doi: 10.1556/aphysiol.97.2010.1.1 20233685

[21] Ménoret A Chandawarkar RY Srivastava PK . Natural autoantibodies against heat-shock proteins hsp70 and gp96: Implications for immunotherapy using heat-shock proteins. Immunology. (2000) 101:364–70. doi: 10.1046/j.1365-2567.2000.00127.x 11106940 PMC2327096

[22] Tukaj S . Dual role of autoantibodies to heat shock proteins in autoimmune diseases. Front Immunol. (2024) 15. doi: 10.3389/fimmu.2024.1421528 38903496 PMC11187000

[23] Holodick NE Rodríguez-Zhurbenko N Hernández AM . Defining natural antibodies. Front Immunol. (2017) 8:872. doi: 10.3389/fimmu.2017.00872 28798747 PMC5526850

[24] Apostolidis SA Lieberman LA Kis-Toth K Crispín JC Tsokos GC . The dysregulation of cytokine networks in systemic lupus erythematosus. J Interferon Cytokine Res. (2011) 31:769–79. doi: 10.1089/jir.2011.0029 21877904 PMC3189553

[25] Mahajan A Herrmann M Muñoz LE . Clearance deficiency and cell death pathways: A model for the pathogenesis of SLE. Front Immunol. (2016) 7:35. doi: 10.3389/fimmu.2016.00035 26904025 PMC4745266

[26] Szinger D Berki T Németh P Erdő-Bonyár S Simon D Drenjančević I . Following natural autoantibodies: Further immunoserological evidence regarding their silent plasticity and engagement in immune activation. Int J Mol Sci. (2023) 24:14961. doi: 10.3390/ijms241914961 37834409 PMC10573785

[27] Tangye SG Ferguson A Avery DT Ma CS Hodgkin PD . Isotype switching by human B cells is division-associated and regulated by cytokines. J Immunol. (2002) 169:4298–306. doi: 10.4049/jimmunol.169.8.4298 12370361

[28] Su J Hua X Concha H Svenungsson E Cederholm A Frostegård J . Natural antibodies against phosphorylcholine as potential protective factors in SLE. Rheumatology. (2008) 47:1144–50. doi: 10.1093/rheumatology/ken120 18522961

[29] Anania C Gustafsson T Hua X Su J Vikström M de Faire U . Increased prevalence of vulnerable atherosclerotic plaques and low levels of natural IgM antibodies against phosphorylcholine in patients with systemic lupus erythematosus. Arthritis Res Ther. (2010) 12:R214. doi: 10.1186/ar3193 21092251 PMC3046524

[30] Chen Y Park Y-B Patel E Silverman GJ . IgM antibodies to apoptosis-associated determinants recruit C1q and enhance dendritic cell phagocytosis of apoptotic cells. J Immunol. (2009) 182:6031–43. doi: 10.4049/jimmunol.0804191 19414754 PMC4428684

[31] Kohler H Bayry J Nicoletti A Kaveri SV . Natural autoantibodies as tools to predict the outcome of immune response? Scand J Immunol. (2003) 58:285–9. doi: 10.1046/j.1365-3083.2003.01314.x 12950673

[32] Maddur MS Kaveri SV Bayry J . Circulating normal IgG as stimulator of regulatory T cells: Lessons from intravenous immunoglobulin. Trends Immunol. (2017) 38:789–92. doi: 10.1016/j.it.2017.08.008 28916232

[33] Nagele EP Han M Acharya NK DeMarshall C Kosciuk MC Nagele RG . Natural IgG autoantibodies are abundant and ubiquitous in human sera, and their number is influenced by age, gender, and disease. PloS One. (2013) 8:e60726. doi: 10.1371/journal.pone.0060726 23589757 PMC3617628

[34] Antonini L Le Mauff B Marcelli C Aouba A De Boysson H . Rhupus: A systematic literature review. Autoimmun Rev. (2020) 19:102612. doi: 10.1016/j.autrev.2020.102612 32668290

[35] Togashi T Ishihara R Watanabe R Shiomi M Yano Y Fujisawa Y . Rheumatoid factor: Diagnostic and prognostic performance and therapeutic implications in rheumatoid arthritis. J Clin Med. (2025) 14:1529. doi: 10.3390/jcm14051529 40094988 PMC11900400

[36] Gómez-Puerta JA Burlingame RW Cervera R . Anti-chromatin (anti-nucleosome) antibodies: Diagnostic and clinical value. Autoimmun Rev. (2008) 7:606–11. doi: 10.1016/j.autrev.2008.06.005 18606252

[37] Bizzaro N Villalta D Giavarina D Tozzoli R . Are anti-nucleosome antibodies a better diagnostic marker than anti-dsDNA antibodies for systemic lupus erythematosus? A systematic review and a study of metanalysis. Autoimmun Rev. (2012) 12:97–106. doi: 10.1016/j.autrev.2012.07.002 22810055

[38] Bournia VK Vlachoyiannopoulos PG . Subgroups of Sjögren syndrome patients according to serological profiles. J Autoimmun. (2012) 39:15–26. doi: 10.1016/j.jaut.2012.03.001 22575069

[39] Martin-Gutierrez L Peng J Thompson NL Robinson GA Naja M Peckham H . Stratification of patients with Sjögren’s syndrome and patients with systemic lupus erythematosus according to two shared immune cell signatures, with potential therapeutic implications. Arthritis Rheumatol. (2021) 73:1626–37. doi: 10.1002/art.41708 33645922

[40] Patriarcheas V Tsamos G Vasdeki D Kotteas E Kollias A Nikas D . Antiphospholipid syndrome: A comprehensive clinical review. J Clin Med. (2025) 14:733. doi: 10.3390/jcm14030733 39941405 PMC11818257

[41] Barbhaiya M Zuily S Naden R Hendry A Manneville F Amigo M-C . 2023 ACR/EULAR antiphospholipid syndrome classification criteria. Ann Rheum Dis. (2023) 82:1258–70. doi: 10.1136/ard-2023-224609 37640450

[42] Očková M Anunciación-Llunell A Andrada C Esteve-Valverde E Miró-Mur F Alijotas-Reig J . IgM antiphospholipid antibodies in antiphospholipid syndrome: Prevalence, clinical associations, and diagnostic implications—a scoping review. J Clin Med. (2025) 14:7164. doi: 10.3390/jcm14207164 41156037 PMC12565476

[43] Simon D Erdő-Bonyár S Böröcz K Balázs N Badawy A Bajnok A . Altered levels of natural autoantibodies against heat shock proteins in pregnant women with Hashimoto’s thyroiditis. Int J Mol Sci. (2024) 25:1423. doi: 10.3390/ijms25031423 38338701 PMC10855109

[44] Böröcz K Kinyó Á Simon D Erdő-Bonyár S Németh P Berki T . Complexity of the immune response elicited by different COVID-19 vaccines, in the light of natural autoantibodies and immunomodulatory therapies. Int J Mol Sci. (2023) 24:6439. doi: 10.3390/ijms24076439 37047412 PMC10094397

[45] Simón JA Cabiedes J Ortiz E Alcocer-Varela J Sánchez-Guerrero J . Anti-nucleosome antibodies in patients with systemic lupus erythematosus of recent onset. Potential utility as a diagnostic tool and disease activity marker. Rheumatology. (2004) 43:220–4. doi: 10.1093/rheumatology/keh024 13130152

[46] Carlé C Fortenfant F Bost C Belliere J Faguer S Chauveau D . The added value of coupling anti-dsDNA and anti-chromatin antibodies in follow-up monitoring of systemic lupus erythematosus patients. J Transl Autoimmun. (2025) 10:100274. doi: 10.1016/j.jtauto.2025.100274 39917317 PMC11799958

[47] Pagkopoulou E Loutradis C Papaioannou M Daoudaki M Stangou M Dimitroulas T . Autoantibodies in systemic lupus erythematosus: Diagnostic and pathogenic insights. J Clin Med. (2025) 14:5714. doi: 10.3390/jcm14165714 40869539 PMC12386345

[48] Santos-Moreno P Arias-Aponte J Rodríguez-Vargas G-S Nieto-Zambrano PD Villarreal L Ibatá L . Polyautoimmunity in systemic lupus erythematosus patients: New insights from a cross-sectional study. J Transl Autoimmun. (2023) 6:100187. doi: 10.1016/j.jtauto.2022.100187 36654852 PMC9841268

[49] Mena-Vázquez N Nebro AF Rúa-Figueroa I Izquierdo MG Ovalles-Bonilla J Olivé-Marqués A . 85 Polyautoimmunity in systemic lupus erythematosus. Data from a large Spanish cohort: Spanish Society of Rheumatology registry of patients with systemic lupus erythematosus (RELESSER). Lupus Sci Med. (2019) 6:1. doi: 10.1136/lupus-2019-lsm.85

[50] Lockshin MD Levine AB Erkan D . Patients with overlap autoimmune disease differ from those with ‘pure’ disease. Lupus Sci Med. (2015) 2:1–6. doi: 10.1136/lupus-2015-000084 25973214 PMC4422903

[51] Wichainun R Kasitanon N Wangkaew S Hongsongkiat S Sukitawut W Louthrenoo W . Sensitivity and specificity of ANA and anti-dsDNA in the diagnosis of systemic lupus erythematosus: a comparison using control sera obtained from healthy individuals and patients with multiple medical problems. Asian Pac J Allergy Immunol. (2013) 31:292–8. doi: 10.12932/AP0272.31.4.2013 24383972

[52] Andreoli L Pregnolato F Burlingame RW Allegri F Rizzini S Fanelli V . Antinucleosome antibodies in primary antiphospholipid syndrome: a hint at systemic autoimmunity? J Autoimmun. (2008) 30:51–7. doi: 10.1016/j.jaut.2007.11.004 18191541

[53] Pons-Estel GJ Andreoli L Scanzi F Cervera R Tincani A . The antiphospholipid syndrome in patients with systemic lupus erythematosus. J Autoimmun. (2017) 76:10–20. doi: 10.1016/J.JAUT.2016.10.004 27776934

[54] De Marchi G Nano A Fulvio G Manfrè V Navarro Garcia IC Zabotti A . Sjögren’s disease and systemic lupus erythematosus overlap syndrome as distinct entity at crossroads of two autoimmune disorders: clinical characterisation from two Italian reference centres for both the diseases. Clin Exp Rheumatol. (2024) 42:2453–8. doi: 10.55563/clinexprheumatol/7v6qht 39656592

[55] Kitching AR Anders H-J Basu N . ANCA-associated vasculitis. Nat Rev Dis Primer. (2020) 6:71. doi: 10.1038/s41572-020-0204-y 32855422

[56] Devreese KMJ Zuily S Meroni PL . Role of antiphospholipid antibodies in the diagnosis of antiphospholipid syndrome. J Transl Autoimmun. (2021) 4:100134. doi: 10.1016/j.jtauto.2021.100134 34816113 PMC8592860

[57] Garcia D Erkan D . Diagnosis and management of the antiphospholipid syndrome. N Engl J Med. (2018) 378:2010–21. doi: 10.1056/NEJMRA1705454 29791828

[58] Brandt JE Priori R Valesini G Fairweather D . Sex differences in Sjögren’s syndrome: a comprehensive review of immune mechanisms. Biol Sex Differ. (2015) 6:19. doi: 10.1186/s13293-015-0037-7 26535108 PMC4630965

[59] Kasaian MT Ikematsu H Balow JE Casali P . Structure of the VH and VL segments of mono reactive and polyreactive IgA autoantibodies to DNA in patients with systemic lupus erythematosus. J Immunol Baltim Md 1950. (1994) 152:3137–51. doi: 10.4049/jimmunol.152.6.3137 PMC4631053 8144908

[60] Elkon K Casali P . Nature and functions of autoantibodies. Nat Clin Pract Rheumatol. (2008) 4:491–8. doi: 10.1038/ncprheum0895 18756274 PMC2703183

[61] Pisetsky DS . Anti-DNA antibodies--quintessential biomarkers of SLE. Nat Rev Rheumatol. (2016) 12:102–10. doi: 10.1038/nrrheum.2015.151 26581343

