# Supplementary material for: Isotype-dependent shifts of natural autoantibody profiles in systemic lupus erythematosus-associated serological groups
Source: Front Immunol. 2026 Oct 30;17:1936421. doi: 10.3389/fimmu.2026.1936421 (PMC13759731; doi:10.3389/fimmu.2026.1936421)
Supplement: Supplementary file 1 [file Table1.docx]

**Supplementary Table S1. Laboratory-validated ELISA cut-offs, testing frequencies, numbers of tested and co-tested patients (denominators), and pAAb positivity and co-positivity ratios with exact 95% Clopper–Pearson confidence intervals and frequency tiers underlying Figure 1.**

| **Tested analytes** | **Laboratory-validated cut-off values (positive if above)** | **Single/co-testing** | **Test count per analyte(s) (n = 128,847 total)** | **Overall testing frequency  (% of total test requests; n=128,847)** | **Number of patients tested per analyte(s)** | **Testing frequency relative to de-duplicated patients (% of total number of unique patients n=73,747)** | **Number of positive/co‑positive patients** | **Positivity ratio  (% of number of tested patients)** | **CI low (%)** | **CI high (%)** | **Error - (%)** | **Error + (%)** | **Frequency cluster** | **Positivity ratio within the a-dsDNA IgG+ subgroup (% of a-dsDNA IgG+ patients)** | **CI low (%)** | **CI high (%)** | **Error - (%)** | **Error + (%)** | **Frequency cluster** |
| --- | --- | --- | --- | --- | --- | --- | --- | --- | --- | --- | --- | --- | --- | --- | --- | --- | --- | --- | --- |
| **ANA screen** | **> 10 / > 20 U/mL†** | **single analyte** | **63244** | **49.08** | **41764** | **56.63** | **7245** | **17.35** | **16.99** | **17.71** | **0.36** | **0.36** | **High (I)** | **−** | **−** | **−** | **−** | **−** | **−** |
| **a-dsDNA IgG** | **> 20 IU/mL** | **single analyte** | **42952** | **33.34** | **27118** | **36.77** | **2447** | **9.02** | **8.69** | **9.37** | **0.33** | **0.35** | **High (I)** | **−** | **−** | **−** | **−** | **−** | **−** |
| **a-Nuc IgG** | **> 20 U/mL** | **single analyte** | **45998** | **35.70** | **31590** | **42.84** | **3672** | **11.62** | **11.27** | **11.98** | **0.35** | **0.36** | **High (I)** | **−** | **−** | **−** | **−** | **−** | **−** |
| **a-CL/β2GPI IgM** | **a-CL IgM > 7 U/mL; a-β2GPI IgM > 8 U/mL** | **single analyte** | **44943** | **34.88** | **30561** | **41.44** | **1413** | **4.62** | **4.39** | **4.86** | **0.23** | **0.24** | **Intermediate (II)** | **−** | **−** | **−** | **−** | **−** | **−** |
| **a-CL/β2GPI IgG** | **a-CL IgG > 10 U/mL; a-β2GPI IgG > 8 U/mL** | **single analyte** | **44943** | **34.88** | **30561** | **41.44** | **859** | **2.81** | **2.63** | **3.00** | **0.18** | **0.19** | **Intermediate (II)** | **−** | **−** | **−** | **−** | **−** | **−** |
| **a-PT IgG** | **> 10 U/mL** | **single analyte** | **31224** | **24.23** | **22727** | **30.82** | **630** | **2.77** | **2.56** | **2.99** | **0.21** | **0.22** | **Intermediate (II)** | **−** | **−** | **−** | **−** | **−** | **−** |
| **a-PT IgM** | **> 10 U/mL** | **single analyte** | **31224** | **24.23** | **22727** | **30.82** | **367** | **1.61** | **1.45** | **1.79** | **0.16** | **0.18** | **Intermediate (II)** | **−** | **−** | **−** | **−** | **−** | **−** |
| **ENA screen** | **≥ 1.0 RU/mL‡** | **single analyte** | **50894** | **39.50** | **–** | **–** | **–** | **–** | **–** | **–** | **–** | **–** | **–** | **−** | **−** | **−** | **−** | **−** | **−** |
| **a-SSA/Ro IgG** | **> 15 / > 25 U/mL*** | **single analyte** | **50894** | **39.50** | **22076** | **29.93** | **1288** | **5.83** | **5.53** | **6.15** | **0.30** | **0.32** | **High (I)** | **−** | **−** | **−** | **−** | **−** | **−** |
| **a-SSB/La IgG** | **> 15 / > 25 U/mL*** | **single analyte** | **50894** | **39.50** | **22076** | **29.93** | **495** | **2.24** | **2.05** | **2.45** | **0.19** | **0.21** | **Intermediate (II)** | **−** | **−** | **−** | **−** | **−** | **−** |
| **a-Sm IgG** | **> 15 / > 25 U/mL*** | **single analyte** | **50894** | **39.50** | **22076** | **29.93** | **265** | **1.20** | **1.06** | **1.35** | **0.14** | **0.15** | **Intermediate (II)** | **−** | **−** | **−** | **−** | **−** | **−** |
| **a-U1-RNP IgG** | **> 15 / > 25 U/mL*** | **single analyte** | **50894** | **39.50** | **22076** | **29.93** | **303** | **1.37** | **1.22** | **1.53** | **0.15** | **0.16** | **Intermediate (II)** | **−** | **−** | **−** | **−** | **−** | **−** |
| **a-Scl-70 IgG** | **> 15 / > 25 U/mL*** | **single analyte** | **50894** | **39.50** | **22076** | **29.93** | **142** | **0.64** | **0.54** | **0.76** | **0.10** | **0.12** | **Low (III)** | **−** | **−** | **−** | **−** | **−** | **−** |
| **a-Jo-1 IgG** | **> 15 / > 25 U/mL*** | **single analyte** | **50894** | **39.50** | **22076** | **29.93** | **310** | **1.40** | **1.25** | **1.57** | **0.15** | **0.17** | **Intermediate (II)** | **−** | **−** | **−** | **−** | **−** | **−** |
| **a-PR3 IgG (c-ANCA)** | **> 5 RU/mL (hsPR3 > 10 RU/mL)** | **single analyte** | **44272** | **34.36** | **32299** | **43.80** | **380** | **1.18** | **1.06** | **1.30** | **0.12** | **0.12** | **Intermediate (II)** | **−** | **−** | **−** | **−** | **−** | **−** |
| **a-MPO IgG (p-ANCA)** | **> 5 RU/mL** | **single analyte** | **44272** | **34.36** | **32299** | **43.80** | **322** | **1.00** | **0.89** | **1.11** | **0.11** | **0.11** | **Intermediate (II)** | **−** | **−** | **−** | **−** | **−** | **−** |
| **a-dsDNA IgG, a-Nuc IgG (chromatin co-reactivity)** | **For co-positivity analyses, the relevant combinations of the above-listed markers were evaluated using the corresponding marker-specific cut-off values.** | **co-testing** | **30061** | **23.33** | **19833** | **26.89** | **1028** | **5.18** | **4.88** | **5.50** | **0.30** | **0.32** | **High (I)** | **42.01** | **40.05** | **44.00** | **1.96** | **1.99** | **High (I)** |
| **a-dsDNA IgG–a-CL/β2GPI IgG/M  (irrespective of isotype)** | **For co-positivity analyses, the relevant combinations of the above-listed markers were evaluated using the corresponding marker-specific cut-off values.** | **co-testing** | **28244** | **21.92** | **19375** | **26.27** | **389** | **2.01** | **1.81** | **2.22** | **0.20** | **0.21** | **Intermediate (II)** | **15.90** | **14.47** | **17.41** | **1.43** | **1.51** | **High (I)** |
| **a-dsDNA IgG, a-CL/β2GPI IgM** |  | **co-testing** | **28244** | **21.92** | **19375** | **26.27** | **273** | **1.41** | **1.25** | **1.59** | **0.16** | **0.18** | **Intermediate (II)** | **11.16** | **9.94** | **12.47** | **1.22** | **1.31** | **High (I)** |
| **a-dsDNA IgG, a-CL/β2GPI IgG** |  | **co-testing** | **28244** | **21.92** | **19375** | **26.27** | **220** | **1.14** | **0.99** | **1.29** | **0.15** | **0.15** | **Intermediate (II)** | **8.99** | **7.89** | **10.19** | **1.10** | **1.20** | **Intermediate (II)** |
| **a-dsDNA IgG, a-PT IgM** |  | **co-testing** | **18329** | **14.23** | **13284** | **18.01** | **58** | **0.44** | **0.33** | **0.56** | **0.11** | **0.12** | **Low (III)** | **2.37** | **1.80** | **3.05** | **0.57** | **0.68** | **Low (III)** |
| **a-dsDNA IgG, a-PT IgG** |  | **co-testing** | **18329** | **14.23** | **13284** | **18.01** | **110** | **0.83** | **0.68** | **1.00** | **0.15** | **0.17** | **Low (III)** | **4.50** | **3.71** | **5.39** | **0.79** | **0.89** | **Intermediate (II)** |
| **a-dsDNA IgG with  any ENA antigen-specific positivity** |  | **co-testing** | **33220** | **25.78** | **13909** | **18.86** | **404** | **2.90** | **2.63** | **3.20** | **0.27** | **0.30** | **Intermediate (II)** | **16.51** | **15.06** | **18.04** | **1.45** | **1.53** | **High (I)** |
| **a-dsDNA IgG, ENA subpanel  (SSA/Ro, SSB/La, Sm, U1-RNP)** |  | **co-testing** | **33220** | **25.78** | **13909** | **18.86** | **388** | **2.79** | **2.52** | **3.08** | **0.27** | **0.29** | **Intermediate (II)** | **15.86** | **14.43** | **17.36** | **1.43** | **1.50** | **High (I)** |
| **a-dsDNA IgG, a-SSA/Ro IgG** |  | **co-testing** | **33220** | **25.78** | **13909** | **18.86** | **281** | **2.02** | **1.79** | **2.27** | **0.23** | **0.25** | **Intermediate (II)** | **11.48** | **10.25** | **12.81** | **1.23** | **1.33** | **High (I)** |
| **a-dsDNA IgG, a-SSB/La IgG** |  | **co-testing** | **33220** | **25.78** | **13909** | **18.86** | **120** | **0.86** | **0.72** | **1.03** | **0.14** | **0.17** | **Low (III)** | **4.90** | **4.08** | **5.84** | **0.82** | **0.94** | **Intermediate (II)** |
| **a-dsDNA IgG, a-Sm IgG** |  | **co-testing** | **33220** | **25.78** | **13909** | **18.86** | **85** | **0.61** | **0.49** | **0.76** | **0.12** | **0.15** | **Low (III)** | **3.47** | **2.78** | **4.28** | **0.69** | **0.81** | **Intermediate (II)** |
| **a-dsDNA IgG, a-U1-RNP IgG** |  | **co-testing** | **33220** | **25.78** | **13909** | **18.86** | **112** | **0.81** | **0.66** | **0.97** | **0.15** | **0.16** | **Low (III)** | **4.58** | **3.78** | **5.48** | **0.80** | **0.90** | **Intermediate (II)** |
| **a-dsDNA IgG, a-Scl-70 IgG** |  | **co-testing** | **33220** | **25.78** | **13909** | **18.86** | **15** | **0.11** | **0.06** | **0.18** | **0.05** | **0.07** | **Low (III)** | **0.61** | **0.34** | **1.01** | **0.27** | **0.40** | **Low (III)** |
| **a-dsDNA IgG, a-Jo-1 IgG** |  | **co-testing** | **33220** | **25.78** | **13909** | **18.86** | **37** | **0.27** | **0.19** | **0.37** | **0.08** | **0.10** | **Low (III)** | **1.51** | **1.07** | **2.08** | **0.44** | **0.57** | **Low (III)** |
| **a-dsDNA IgG, ANCAs** |  | **co-testing** | **21653** | **16.81** | **16860** | **22.86** | **74** | **0.44** | **0.34** | **0.55** | **0.10** | **0.11** | **Low (III)** | **3.02** | **2.38** | **3.78** | **0.64** | **0.76** | **Intermediate (II)** |
| **a-dsDNA IgG, a-PR3 IgG (c-ANCA)** |  | **co-testing** | **21653** | **16.81** | **16860** | **22.86** | **31** | **0.18** | **0.12** | **0.26** | **0.06** | **0.08** | **Low (III)** | **1.27** | **0.86** | **1.79** | **0.41** | **0.52** | **Low (III)** |
| **a-dsDNA IgG, a-MPO IgG (p-ANCA)** |  | **co-testing** | **21653** | **16.81** | **16860** | **22.86** | **57** | **0.34** | **0.26** | **0.44** | **0.08** | **0.10** | **Low (III)** | **2.33** | **1.77** | **3.01** | **0.56** | **0.68** | **Low (III)** |

The table summarises the tested analytes, laboratory-validated ELISA cut-off values, single- or co-testing status, total test counts, testing frequencies, de-duplicated patient numbers, and positivity/co-positivity ratios for the autoimmune diagnostic markers assessed in the regional laboratory cohort (73,747 patients aged 18–80 years, 2014–2024; 65.67% women, median age 53 years, IQR 39–67; sex distribution in Supplementary Table S3). Testing frequency was calculated relative to the total number of test requests (n = 128,847) and to the total number of unique patients (n = 73,747). Single-analyte positivity ratios were calculated using the number of patients tested within the corresponding diagnostic panel (screening and/or antigen-specific assay) as the denominator; co-positivity ratios used the number of patients co-tested for a-dsDNA IgG and the corresponding panel in the same sample, and a-dsDNA IgG-associated combinations were additionally evaluated within the a-dsDNA IgG-positive subgroup (n = 2,447). For each analyte, positive results (for co-positivity, a-dsDNA IgG-positive samples that were also positive for the given marker) were identified first, ordered by patient and test date, and only the first chronological qualifying result per patient was counted. Ratios are presented with exact (Clopper–Pearson) 95% confidence intervals. ENA screening results were not used for positivity (antigen-specific results only); ENA = any of a-SSA/Ro, a-SSB/La, a-Sm, a-U1-RNP, a-Scl-70 and a-Jo-1; ENA subpanel = any of a-SSA/Ro, a-SSB/La, a-Sm and a-U1-RNP; ANCAs = a-PR3 and/or a-MPO IgG. Frequency clusters (tiers): single-analyte and co-testing ratios, high (I) ≥ 5%, intermediate (II) 1–4.99%, low (III) < 1%; within the a-dsDNA IgG-positive subgroup, high (I) ≥ 10%, intermediate (II) 3–9.99%, low (III) < 3%. *ENA antigen-specific assays: > 15 U/mL for the 2014–2020 assay and > 25 U/mL for the assay used from 2020. †ANA screen: > 10 U/mL for 2014–2016 (HYCOR Biomedical) and > 20 U/mL from 1 January 2017 (Werfen/Inova). ‡ENA screen: testing burden only; screening results were not used for positivity. hsPR3, high-sensitivity a-PR3 assay. For the a-dsDNA IgG with a-Nuc IgG combination (same antigenic target, chromatin), the test count is the number of samples tested for both analytes. “–” indicates not applicable.

Abbreviations: ANA, antinuclear antibody; a-dsDNA, anti-double-stranded DNA; a-Nuc, anti-nucleosome; a-CL/β2GPI, anti-cardiolipin/anti-β2-glycoprotein I; a-PT, anti-prothrombin; ENA, extractable nuclear antigen; a-SSA/Ro, anti-SSA/Ro; a-SSB/La, anti-SSB/La; a-Sm, anti-Smith; a-U1-RNP, anti-U1-ribonucleoprotein; a-Scl-70, anti-topoisomerase I; a-Jo-1, anti-histidyl-tRNA synthetase; ANCA, anti-neutrophil cytoplasmic antibody; a-PR3, anti-proteinase 3; a-MPO, anti-myeloperoxidase; CI, confidence interval.
