# Supplementary material for: Isotype-dependent shifts of natural autoantibody profiles in systemic lupus erythematosus-associated serological groups
Source: Front Immunol. 2026 Oct 30;17:1936421. doi: 10.3389/fimmu.2026.1936421 (PMC13759731; doi:10.3389/fimmu.2026.1936421)
Supplement: Supplementary file 2 [file Table2.docx]

**Supplementary Table S2.** Complete intersection (UpSet) and Venn diagram data underlying Figures 2 and 3.

**(A) Set sizes.** Number and percentage of patients positive for each specificity in Panel A (all pAAb-positive patients, n = 7,721) and Panel B (a-dsDNA IgG-positive patients, n = 2,447) of Figure 2.

| **Specificity** | **Panel A positive (n)** | **% of Panel A (n = 7,721)** | **Panel B positive (n)** | **% of Panel B (n = 2,447)** |
| --- | --- | --- | --- | --- |
| a-dsDNA IgG | 2286 | 29.61 | 2447 | 100 |
| a-Nuc IgG | 3510 | 45.46 | – | – |
| a-SSA/Ro IgG | 1216 | 15.75 | 265 | 10.83 |
| a-SSB/La IgG | 458 | 5.93 | 106 | 4.33 |
| a-Sm IgG | 234 | 3.03 | 75 | 3.06 |
| a-U1-RNP IgG | 267 | 3.46 | 97 | 3.96 |
| a-Scl-70 IgG | 136 | 1.76 | 14 | 0.57 |
| a-Jo-1 IgG | 284 | 3.68 | 30 | 1.23 |
| a-PR3 IgG (c-ANCA) | 363 | 4.70 | 27 | 1.10 |
| a-MPO IgG (p-ANCA) | 294 | 3.81 | 51 | 2.08 |
| a-CL/β2GPI IgG/M | 1813 | 23.48 | 361 | 14.75 |

**(B) Panel A intersections.** Figure 2A: all exclusive antibody combinations among the 7,721 patients positive for ≥ 1 of the 11 specificities, ranked by frequency. In the specificity columns, 1 = positive, 0 = negative or not tested.

| **Rank** | **Combination** | **Specificities (n)** | **Patients (n)** | **% of panel** | **a-dsDNA** | **a-Nuc** | **a-SSA/Ro** | **a-SSB/La** | **a-Sm** | **a-U1-RNP** | **a-Scl-70** | **a-Jo-1** | **a-PR3** | **a-MPO** | **a-CL/β2GPI** |
| --- | --- | --- | --- | --- | --- | --- | --- | --- | --- | --- | --- | --- | --- | --- | --- |
| 1 | a-Nuc IgG | 1 | 2037 | 26.38 | 0 | 1 | 0 | 0 | 0 | 0 | 0 | 0 | 0 | 0 | 0 |
| 2 | a-CL/β2GPI IgG/M | 1 | 1275 | 16.51 | 0 | 0 | 0 | 0 | 0 | 0 | 0 | 0 | 0 | 0 | 1 |
| 3 | a-dsDNA IgG only | 1 | 1052 | 13.63 | 1 | 0 | 0 | 0 | 0 | 0 | 0 | 0 | 0 | 0 | 0 |
| 4 | a-dsDNA IgG + a-Nuc IgG | 2 | 647 | 8.38 | 1 | 1 | 0 | 0 | 0 | 0 | 0 | 0 | 0 | 0 | 0 |
| 5 | a-SSA/Ro IgG | 1 | 394 | 5.10 | 0 | 0 | 1 | 0 | 0 | 0 | 0 | 0 | 0 | 0 | 0 |
| 6 | a-PR3 IgG (c-ANCA) | 1 | 293 | 3.79 | 0 | 0 | 0 | 0 | 0 | 0 | 0 | 0 | 1 | 0 | 0 |
| 7 | a-SSA/Ro IgG + a-SSB/La IgG | 2 | 212 | 2.75 | 0 | 0 | 1 | 1 | 0 | 0 | 0 | 0 | 0 | 0 | 0 |
| 8 | a-MPO IgG (p-ANCA) | 1 | 184 | 2.38 | 0 | 0 | 0 | 0 | 0 | 0 | 0 | 0 | 0 | 1 | 0 |
| 9 | a-dsDNA IgG + a-CL/β2GPI IgG/M | 2 | 157 | 2.03 | 1 | 0 | 0 | 0 | 0 | 0 | 0 | 0 | 0 | 0 | 1 |
| 10 | a-Nuc IgG + a-CL/β2GPI IgG/M | 2 | 135 | 1.75 | 0 | 1 | 0 | 0 | 0 | 0 | 0 | 0 | 0 | 0 | 1 |
| 11 | a-Nuc IgG + a-SSA/Ro IgG | 2 | 100 | 1.30 | 0 | 1 | 1 | 0 | 0 | 0 | 0 | 0 | 0 | 0 | 0 |
| 12 | a-dsDNA IgG + a-Nuc IgG + a-CL/β2GPI IgG/M | 3 | 79 | 1.02 | 1 | 1 | 0 | 0 | 0 | 0 | 0 | 0 | 0 | 0 | 1 |
| 13 | a-Jo-1 IgG | 1 | 70 | 0.91 | 0 | 0 | 0 | 0 | 0 | 0 | 0 | 1 | 0 | 0 | 0 |
| 14 | a-SSA/Ro IgG + a-Sm IgG + a-Jo-1 IgG | 3 | 64 | 0.83 | 0 | 0 | 1 | 0 | 1 | 0 | 0 | 1 | 0 | 0 | 0 |
| 15 | a-Nuc IgG + a-U1-RNP IgG | 2 | 64 | 0.83 | 0 | 1 | 0 | 0 | 0 | 1 | 0 | 0 | 0 | 0 | 0 |
| 16 | a-SSB/La IgG | 1 | 63 | 0.82 | 0 | 0 | 0 | 1 | 0 | 0 | 0 | 0 | 0 | 0 | 0 |
| 17 | a-Nuc IgG + a-Scl-70 IgG | 2 | 61 | 0.79 | 0 | 1 | 0 | 0 | 0 | 0 | 1 | 0 | 0 | 0 | 0 |
| 18 | a-dsDNA IgG + a-Nuc IgG + a-SSA/Ro IgG | 3 | 53 | 0.69 | 1 | 1 | 1 | 0 | 0 | 0 | 0 | 0 | 0 | 0 | 0 |
| 19 | a-Nuc IgG + a-SSA/Ro IgG + a-SSB/La IgG | 3 | 47 | 0.61 | 0 | 1 | 1 | 1 | 0 | 0 | 0 | 0 | 0 | 0 | 0 |
| 20 | a-dsDNA IgG + a-SSA/Ro IgG | 2 | 42 | 0.54 | 1 | 0 | 1 | 0 | 0 | 0 | 0 | 0 | 0 | 0 | 0 |
| 21 | a-SSA/Ro IgG + a-CL/β2GPI IgG/M | 2 | 34 | 0.44 | 0 | 0 | 1 | 0 | 0 | 0 | 0 | 0 | 0 | 0 | 1 |
| 22 | a-U1-RNP IgG | 1 | 33 | 0.43 | 0 | 0 | 0 | 0 | 0 | 1 | 0 | 0 | 0 | 0 | 0 |
| 23 | a-Sm IgG + a-Jo-1 IgG | 2 | 27 | 0.35 | 0 | 0 | 0 | 0 | 1 | 0 | 0 | 1 | 0 | 0 | 0 |
| 24 | a-dsDNA IgG + a-Nuc IgG + a-SSA/Ro IgG + a-SSB/La IgG | 4 | 26 | 0.34 | 1 | 1 | 1 | 1 | 0 | 0 | 0 | 0 | 0 | 0 | 0 |
| 25 | a-dsDNA IgG + a-SSA/Ro IgG + a-SSB/La IgG | 3 | 24 | 0.31 | 1 | 0 | 1 | 1 | 0 | 0 | 0 | 0 | 0 | 0 | 0 |
| 26 | a-SSA/Ro IgG + a-Jo-1 IgG | 2 | 23 | 0.30 | 0 | 0 | 1 | 0 | 0 | 0 | 0 | 1 | 0 | 0 | 0 |
| 27 | a-Nuc IgG + a-MPO IgG (p-ANCA) | 2 | 21 | 0.27 | 0 | 1 | 0 | 0 | 0 | 0 | 0 | 0 | 0 | 1 | 0 |
| 28 | a-dsDNA IgG + a-MPO IgG (p-ANCA) | 2 | 21 | 0.27 | 1 | 0 | 0 | 0 | 0 | 0 | 0 | 0 | 0 | 1 | 0 |
| 29 | a-dsDNA IgG + a-Nuc IgG + a-U1-RNP IgG | 3 | 18 | 0.23 | 1 | 1 | 0 | 0 | 0 | 1 | 0 | 0 | 0 | 0 | 0 |
| 30 | a-Scl-70 IgG | 1 | 16 | 0.21 | 0 | 0 | 0 | 0 | 0 | 0 | 1 | 0 | 0 | 0 | 0 |
| 31 | a-PR3 IgG (c-ANCA) + a-MPO IgG (p-ANCA) | 2 | 15 | 0.19 | 0 | 0 | 0 | 0 | 0 | 0 | 0 | 0 | 1 | 1 | 0 |
| 32 | a-dsDNA IgG + a-Nuc IgG + a-Sm IgG + a-U1-RNP IgG | 4 | 15 | 0.19 | 1 | 1 | 0 | 0 | 1 | 1 | 0 | 0 | 0 | 0 | 0 |
| 33 | a-Nuc IgG + a-Sm IgG + a-U1-RNP IgG | 3 | 13 | 0.17 | 0 | 1 | 0 | 0 | 1 | 1 | 0 | 0 | 0 | 0 | 0 |
| 34 | a-dsDNA IgG + a-Nuc IgG + a-SSA/Ro IgG + a-CL/β2GPI IgG/M | 4 | 13 | 0.17 | 1 | 1 | 1 | 0 | 0 | 0 | 0 | 0 | 0 | 0 | 1 |
| 35 | a-MPO IgG (p-ANCA) + a-CL/β2GPI IgG/M | 2 | 12 | 0.16 | 0 | 0 | 0 | 0 | 0 | 0 | 0 | 0 | 0 | 1 | 1 |
| 36 | a-Nuc IgG + a-SSA/Ro IgG + a-U1-RNP IgG | 3 | 12 | 0.16 | 0 | 1 | 1 | 0 | 0 | 1 | 0 | 0 | 0 | 0 | 0 |
| 37 | a-PR3 IgG (c-ANCA) + a-CL/β2GPI IgG/M | 2 | 11 | 0.14 | 0 | 0 | 0 | 0 | 0 | 0 | 0 | 0 | 1 | 0 | 1 |
| 38 | a-Nuc IgG + a-SSA/Ro IgG + a-CL/β2GPI IgG/M | 3 | 11 | 0.14 | 0 | 1 | 1 | 0 | 0 | 0 | 0 | 0 | 0 | 0 | 1 |
| 39 | a-Sm IgG | 1 | 11 | 0.14 | 0 | 0 | 0 | 0 | 1 | 0 | 0 | 0 | 0 | 0 | 0 |
| 40 | a-Nuc IgG + a-PR3 IgG (c-ANCA) | 2 | 9 | 0.12 | 0 | 1 | 0 | 0 | 0 | 0 | 0 | 0 | 1 | 0 | 0 |
| 41 | a-SSA/Ro IgG + a-Scl-70 IgG + a-Jo-1 IgG | 3 | 8 | 0.10 | 0 | 0 | 1 | 0 | 0 | 0 | 1 | 1 | 0 | 0 | 0 |
| 42 | a-dsDNA IgG + a-SSA/Ro IgG + a-CL/β2GPI IgG/M | 3 | 8 | 0.10 | 1 | 0 | 1 | 0 | 0 | 0 | 0 | 0 | 0 | 0 | 1 |
| 43 | a-dsDNA IgG + a-PR3 IgG (c-ANCA) + a-MPO IgG (p-ANCA) | 3 | 7 | 0.09 | 1 | 0 | 0 | 0 | 0 | 0 | 0 | 0 | 1 | 1 | 0 |
| 44 | a-Nuc IgG + a-SSA/Ro IgG + a-Jo-1 IgG | 3 | 7 | 0.09 | 0 | 1 | 1 | 0 | 0 | 0 | 0 | 1 | 0 | 0 | 0 |
| 45 | a-Nuc IgG + a-SSB/La IgG | 2 | 7 | 0.09 | 0 | 1 | 0 | 1 | 0 | 0 | 0 | 0 | 0 | 0 | 0 |
| 46 | a-Nuc IgG + a-SSA/Ro IgG + a-SSB/La IgG + a-U1-RNP IgG | 4 | 7 | 0.09 | 0 | 1 | 1 | 1 | 0 | 1 | 0 | 0 | 0 | 0 | 0 |
| 47 | a-dsDNA IgG + a-PR3 IgG (c-ANCA) | 2 | 7 | 0.09 | 1 | 0 | 0 | 0 | 0 | 0 | 0 | 0 | 1 | 0 | 0 |
| 48 | a-SSA/Ro IgG + a-SSB/La IgG + a-Jo-1 IgG | 3 | 7 | 0.09 | 0 | 0 | 1 | 1 | 0 | 0 | 0 | 1 | 0 | 0 | 0 |
| 49 | a-dsDNA IgG + a-SSB/La IgG | 2 | 6 | 0.08 | 1 | 0 | 0 | 1 | 0 | 0 | 0 | 0 | 0 | 0 | 0 |
| 50 | a-Nuc IgG + a-MPO IgG (p-ANCA) + a-CL/β2GPI IgG/M | 3 | 6 | 0.08 | 0 | 1 | 0 | 0 | 0 | 0 | 0 | 0 | 0 | 1 | 1 |
| 51 | a-dsDNA IgG + a-Nuc IgG + a-MPO IgG (p-ANCA) | 3 | 6 | 0.08 | 1 | 1 | 0 | 0 | 0 | 0 | 0 | 0 | 0 | 1 | 0 |
| 52 | a-Nuc IgG + a-SSA/Ro IgG + a-Sm IgG + a-U1-RNP IgG | 4 | 6 | 0.08 | 0 | 1 | 1 | 0 | 1 | 1 | 0 | 0 | 0 | 0 | 0 |
| 53 | a-dsDNA IgG + a-Sm IgG + a-U1-RNP IgG | 3 | 6 | 0.08 | 1 | 0 | 0 | 0 | 1 | 1 | 0 | 0 | 0 | 0 | 0 |
| 54 | a-dsDNA IgG + a-U1-RNP IgG | 2 | 5 | 0.06 | 1 | 0 | 0 | 0 | 0 | 1 | 0 | 0 | 0 | 0 | 0 |
| 55 | a-SSA/Ro IgG + a-U1-RNP IgG | 2 | 5 | 0.06 | 0 | 0 | 1 | 0 | 0 | 1 | 0 | 0 | 0 | 0 | 0 |
| 56 | a-dsDNA IgG + a-Nuc IgG + a-U1-RNP IgG + a-CL/β2GPI IgG/M | 4 | 5 | 0.06 | 1 | 1 | 0 | 0 | 0 | 1 | 0 | 0 | 0 | 0 | 1 |
| 57 | a-SSA/Ro IgG + a-SSB/La IgG + a-CL/β2GPI IgG/M | 3 | 5 | 0.06 | 0 | 0 | 1 | 1 | 0 | 0 | 0 | 0 | 0 | 0 | 1 |
| 58 | a-dsDNA IgG + a-Nuc IgG + a-PR3 IgG (c-ANCA) | 3 | 5 | 0.06 | 1 | 1 | 0 | 0 | 0 | 0 | 0 | 0 | 1 | 0 | 0 |
| 59 | a-dsDNA IgG + a-Jo-1 IgG | 2 | 5 | 0.06 | 1 | 0 | 0 | 0 | 0 | 0 | 0 | 1 | 0 | 0 | 0 |
| 60 | a-SSA/Ro IgG + a-U1-RNP IgG + a-Scl-70 IgG + a-Jo-1 IgG | 4 | 5 | 0.06 | 0 | 0 | 1 | 0 | 0 | 1 | 1 | 1 | 0 | 0 | 0 |
| 61 | a-dsDNA IgG + a-Nuc IgG + a-SSB/La IgG | 3 | 5 | 0.06 | 1 | 1 | 0 | 1 | 0 | 0 | 0 | 0 | 0 | 0 | 0 |
| 62 | a-Scl-70 IgG + a-CL/β2GPI IgG/M | 2 | 5 | 0.06 | 0 | 0 | 0 | 0 | 0 | 0 | 1 | 0 | 0 | 0 | 1 |
| 63 | a-dsDNA IgG + a-SSA/Ro IgG + a-Sm IgG + a-Jo-1 IgG | 4 | 4 | 0.05 | 1 | 0 | 1 | 0 | 1 | 0 | 0 | 1 | 0 | 0 | 0 |
| 64 | a-SSA/Ro IgG + a-SSB/La IgG + a-U1-RNP IgG | 3 | 4 | 0.05 | 0 | 0 | 1 | 1 | 0 | 1 | 0 | 0 | 0 | 0 | 0 |
| 65 | a-Nuc IgG + a-Sm IgG + a-U1-RNP IgG + a-Jo-1 IgG | 4 | 4 | 0.05 | 0 | 1 | 0 | 0 | 1 | 1 | 0 | 1 | 0 | 0 | 0 |
| 66 | a-Sm IgG + a-U1-RNP IgG + a-Jo-1 IgG | 3 | 4 | 0.05 | 0 | 0 | 0 | 0 | 1 | 1 | 0 | 1 | 0 | 0 | 0 |
| 67 | a-Nuc IgG + a-Scl-70 IgG + a-CL/β2GPI IgG/M | 3 | 4 | 0.05 | 0 | 1 | 0 | 0 | 0 | 0 | 1 | 0 | 0 | 0 | 1 |
| 68 | a-Jo-1 IgG + a-CL/β2GPI IgG/M | 2 | 4 | 0.05 | 0 | 0 | 0 | 0 | 0 | 0 | 0 | 1 | 0 | 0 | 1 |
| 69 | a-SSA/Ro IgG + a-Sm IgG | 2 | 4 | 0.05 | 0 | 0 | 1 | 0 | 1 | 0 | 0 | 0 | 0 | 0 | 0 |
| 70 | a-dsDNA IgG + a-Nuc IgG + a-SSA/Ro IgG + a-U1-RNP IgG | 4 | 4 | 0.05 | 1 | 1 | 1 | 0 | 0 | 1 | 0 | 0 | 0 | 0 | 0 |
| 71 | a-SSB/La IgG + a-CL/β2GPI IgG/M | 2 | 4 | 0.05 | 0 | 0 | 0 | 1 | 0 | 0 | 0 | 0 | 0 | 0 | 1 |
| 72 | a-Nuc IgG + a-SSA/Ro IgG + a-SSB/La IgG + a-CL/β2GPI IgG/M | 4 | 4 | 0.05 | 0 | 1 | 1 | 1 | 0 | 0 | 0 | 0 | 0 | 0 | 1 |
| 73 | a-dsDNA IgG + a-Nuc IgG + a-SSA/Ro IgG + a-SSB/La IgG + a-CL/β2GPI IgG/M | 5 | 4 | 0.05 | 1 | 1 | 1 | 1 | 0 | 0 | 0 | 0 | 0 | 0 | 1 |
| 74 | a-dsDNA IgG + a-Nuc IgG + a-SSA/Ro IgG + a-Sm IgG + a-U1-RNP IgG | 5 | 4 | 0.05 | 1 | 1 | 1 | 0 | 1 | 1 | 0 | 0 | 0 | 0 | 0 |
| 75 | a-SSA/Ro IgG + a-Sm IgG + a-U1-RNP IgG + a-Scl-70 IgG + a-Jo-1 IgG | 5 | 4 | 0.05 | 0 | 0 | 1 | 0 | 1 | 1 | 1 | 1 | 0 | 0 | 0 |
| 76 | a-SSA/Ro IgG + a-Sm IgG + a-U1-RNP IgG | 3 | 3 | 0.04 | 0 | 0 | 1 | 0 | 1 | 1 | 0 | 0 | 0 | 0 | 0 |
| 77 | a-Nuc IgG + a-Jo-1 IgG | 2 | 3 | 0.04 | 0 | 1 | 0 | 0 | 0 | 0 | 0 | 1 | 0 | 0 | 0 |
| 78 | a-dsDNA IgG + a-Sm IgG + a-Jo-1 IgG | 3 | 3 | 0.04 | 1 | 0 | 0 | 0 | 1 | 0 | 0 | 1 | 0 | 0 | 0 |
| 79 | a-SSA/Ro IgG + a-SSB/La IgG + a-Sm IgG + a-Jo-1 IgG | 4 | 3 | 0.04 | 0 | 0 | 1 | 1 | 1 | 0 | 0 | 1 | 0 | 0 | 0 |
| 80 | a-SSA/Ro IgG + a-Sm IgG + a-U1-RNP IgG + a-Jo-1 IgG | 4 | 3 | 0.04 | 0 | 0 | 1 | 0 | 1 | 1 | 0 | 1 | 0 | 0 | 0 |
| 81 | a-Nuc IgG + a-PR3 IgG (c-ANCA) + a-MPO IgG (p-ANCA) | 3 | 3 | 0.04 | 0 | 1 | 0 | 0 | 0 | 0 | 0 | 0 | 1 | 1 | 0 |
| 82 | a-Sm IgG + a-U1-RNP IgG | 2 | 3 | 0.04 | 0 | 0 | 0 | 0 | 1 | 1 | 0 | 0 | 0 | 0 | 0 |
| 83 | a-Nuc IgG + a-Sm IgG + a-U1-RNP IgG + a-CL/β2GPI IgG/M | 4 | 3 | 0.04 | 0 | 1 | 0 | 0 | 1 | 1 | 0 | 0 | 0 | 0 | 1 |
| 84 | a-Nuc IgG + a-U1-RNP IgG + a-CL/β2GPI IgG/M | 3 | 3 | 0.04 | 0 | 1 | 0 | 0 | 0 | 1 | 0 | 0 | 0 | 0 | 1 |
| 85 | a-dsDNA IgG + a-Sm IgG | 2 | 3 | 0.04 | 1 | 0 | 0 | 0 | 1 | 0 | 0 | 0 | 0 | 0 | 0 |
| 86 | a-Nuc IgG + a-Sm IgG | 2 | 3 | 0.04 | 0 | 1 | 0 | 0 | 1 | 0 | 0 | 0 | 0 | 0 | 0 |
| 87 | a-Nuc IgG + a-SSA/Ro IgG + a-Sm IgG + a-Jo-1 IgG | 4 | 2 | 0.03 | 0 | 1 | 1 | 0 | 1 | 0 | 0 | 1 | 0 | 0 | 0 |
| 88 | a-SSA/Ro IgG + a-PR3 IgG (c-ANCA) | 2 | 2 | 0.03 | 0 | 0 | 1 | 0 | 0 | 0 | 0 | 0 | 1 | 0 | 0 |
| 89 | a-dsDNA IgG + a-Nuc IgG + a-Sm IgG + a-U1-RNP IgG + a-CL/β2GPI IgG/M | 5 | 2 | 0.03 | 1 | 1 | 0 | 0 | 1 | 1 | 0 | 0 | 0 | 0 | 1 |
| 90 | a-dsDNA IgG + a-Jo-1 IgG + a-CL/β2GPI IgG/M | 3 | 2 | 0.03 | 1 | 0 | 0 | 0 | 0 | 0 | 0 | 1 | 0 | 0 | 1 |
| 91 | a-SSA/Ro IgG + a-Sm IgG + a-Scl-70 IgG + a-Jo-1 IgG | 4 | 2 | 0.03 | 0 | 0 | 1 | 0 | 1 | 0 | 1 | 1 | 0 | 0 | 0 |
| 92 | a-dsDNA IgG + a-Nuc IgG + a-SSA/Ro IgG + a-Scl-70 IgG | 4 | 2 | 0.03 | 1 | 1 | 1 | 0 | 0 | 0 | 1 | 0 | 0 | 0 | 0 |
| 93 | a-SSA/Ro IgG + a-SSB/La IgG + a-MPO IgG (p-ANCA) | 3 | 2 | 0.03 | 0 | 0 | 1 | 1 | 0 | 0 | 0 | 0 | 0 | 1 | 0 |
| 94 | a-Nuc IgG + a-SSA/Ro IgG + a-SSB/La IgG + a-Scl-70 IgG | 4 | 2 | 0.03 | 0 | 1 | 1 | 1 | 0 | 0 | 1 | 0 | 0 | 0 | 0 |
| 95 | a-Nuc IgG + a-SSA/Ro IgG + a-Scl-70 IgG | 3 | 2 | 0.03 | 0 | 1 | 1 | 0 | 0 | 0 | 1 | 0 | 0 | 0 | 0 |
| 96 | a-dsDNA IgG + a-Scl-70 IgG | 2 | 2 | 0.03 | 1 | 0 | 0 | 0 | 0 | 0 | 1 | 0 | 0 | 0 | 0 |
| 97 | a-dsDNA IgG + a-Nuc IgG + a-Scl-70 IgG | 3 | 2 | 0.03 | 1 | 1 | 0 | 0 | 0 | 0 | 1 | 0 | 0 | 0 | 0 |
| 98 | a-Nuc IgG + a-Scl-70 IgG + a-Jo-1 IgG | 3 | 2 | 0.03 | 0 | 1 | 0 | 0 | 0 | 0 | 1 | 1 | 0 | 0 | 0 |
| 99 | a-Nuc IgG + a-Sm IgG + a-Jo-1 IgG | 3 | 2 | 0.03 | 0 | 1 | 0 | 0 | 1 | 0 | 0 | 1 | 0 | 0 | 0 |
| 100 | a-dsDNA IgG + a-Nuc IgG + a-Sm IgG + a-Jo-1 IgG | 4 | 2 | 0.03 | 1 | 1 | 0 | 0 | 1 | 0 | 0 | 1 | 0 | 0 | 0 |
| 101 | a-SSA/Ro IgG + a-Scl-70 IgG | 2 | 2 | 0.03 | 0 | 0 | 1 | 0 | 0 | 0 | 1 | 0 | 0 | 0 | 0 |
| 102 | a-dsDNA IgG + a-SSA/Ro IgG + a-Sm IgG + a-U1-RNP IgG | 4 | 2 | 0.03 | 1 | 0 | 1 | 0 | 1 | 1 | 0 | 0 | 0 | 0 | 0 |
| 103 | a-dsDNA IgG + a-Nuc IgG + a-SSA/Ro IgG + a-SSB/La IgG + a-Sm IgG + a-U1-RNP IgG | 6 | 2 | 0.03 | 1 | 1 | 1 | 1 | 1 | 1 | 0 | 0 | 0 | 0 | 0 |
| 104 | a-dsDNA IgG + a-Nuc IgG + a-Sm IgG | 3 | 2 | 0.03 | 1 | 1 | 0 | 0 | 1 | 0 | 0 | 0 | 0 | 0 | 0 |
| 105 | a-dsDNA IgG + a-Nuc IgG + a-SSA/Ro IgG + a-Sm IgG + a-U1-RNP IgG + a-CL/β2GPI IgG/M | 6 | 2 | 0.03 | 1 | 1 | 1 | 0 | 1 | 1 | 0 | 0 | 0 | 0 | 1 |
| 106 | a-Scl-70 IgG + a-Jo-1 IgG | 2 | 2 | 0.03 | 0 | 0 | 0 | 0 | 0 | 0 | 1 | 1 | 0 | 0 | 0 |
| 107 | a-dsDNA IgG + a-Nuc IgG + a-SSA/Ro IgG + a-SSB/La IgG + a-U1-RNP IgG | 5 | 2 | 0.03 | 1 | 1 | 1 | 1 | 0 | 1 | 0 | 0 | 0 | 0 | 0 |
| 108 | a-Nuc IgG + a-SSA/Ro IgG + a-Sm IgG + a-U1-RNP IgG + a-Scl-70 IgG + a-Jo-1 IgG | 6 | 2 | 0.03 | 0 | 1 | 1 | 0 | 1 | 1 | 1 | 1 | 0 | 0 | 0 |
| 109 | a-dsDNA IgG + a-MPO IgG (p-ANCA) + a-CL/β2GPI IgG/M | 3 | 2 | 0.03 | 1 | 0 | 0 | 0 | 0 | 0 | 0 | 0 | 0 | 1 | 1 |
| 110 | a-SSA/Ro IgG + a-SSB/La IgG + a-Scl-70 IgG + a-Jo-1 IgG | 4 | 2 | 0.03 | 0 | 0 | 1 | 1 | 0 | 0 | 1 | 1 | 0 | 0 | 0 |
| 111 | a-dsDNA IgG + a-SSB/La IgG + a-Sm IgG | 3 | 1 | 0.01 | 1 | 0 | 0 | 1 | 1 | 0 | 0 | 0 | 0 | 0 | 0 |
| 112 | a-dsDNA IgG + a-Nuc IgG + a-PR3 IgG (c-ANCA) + a-MPO IgG (p-ANCA) + a-CL/β2GPI IgG/M | 5 | 1 | 0.01 | 1 | 1 | 0 | 0 | 0 | 0 | 0 | 0 | 1 | 1 | 1 |
| 113 | a-Nuc IgG + a-SSB/La IgG + a-U1-RNP IgG | 3 | 1 | 0.01 | 0 | 1 | 0 | 1 | 0 | 1 | 0 | 0 | 0 | 0 | 0 |
| 114 | a-Nuc IgG + a-PR3 IgG (c-ANCA) + a-CL/β2GPI IgG/M | 3 | 1 | 0.01 | 0 | 1 | 0 | 0 | 0 | 0 | 0 | 0 | 1 | 0 | 1 |
| 115 | a-dsDNA IgG + a-SSA/Ro IgG + a-Sm IgG + a-Jo-1 IgG + a-CL/β2GPI IgG/M | 5 | 1 | 0.01 | 1 | 0 | 1 | 0 | 1 | 0 | 0 | 1 | 0 | 0 | 1 |
| 116 | a-Sm IgG + a-CL/β2GPI IgG/M | 2 | 1 | 0.01 | 0 | 0 | 0 | 0 | 1 | 0 | 0 | 0 | 0 | 0 | 1 |
| 117 | a-Nuc IgG + a-SSA/Ro IgG + a-SSB/La IgG + a-Jo-1 IgG | 4 | 1 | 0.01 | 0 | 1 | 1 | 1 | 0 | 0 | 0 | 1 | 0 | 0 | 0 |
| 118 | a-dsDNA IgG + a-U1-RNP IgG + a-CL/β2GPI IgG/M | 3 | 1 | 0.01 | 1 | 0 | 0 | 0 | 0 | 1 | 0 | 0 | 0 | 0 | 1 |
| 119 | a-SSB/La IgG + a-Jo-1 IgG + a-CL/β2GPI IgG/M | 3 | 1 | 0.01 | 0 | 0 | 0 | 1 | 0 | 0 | 0 | 1 | 0 | 0 | 1 |
| 120 | a-dsDNA IgG + a-Nuc IgG + a-SSA/Ro IgG + a-Jo-1 IgG | 4 | 1 | 0.01 | 1 | 1 | 1 | 0 | 0 | 0 | 0 | 1 | 0 | 0 | 0 |
| 121 | a-SSA/Ro IgG + a-U1-RNP IgG + a-CL/β2GPI IgG/M | 3 | 1 | 0.01 | 0 | 0 | 1 | 0 | 0 | 1 | 0 | 0 | 0 | 0 | 1 |
| 122 | a-dsDNA IgG + a-Sm IgG + a-U1-RNP IgG + a-Jo-1 IgG | 4 | 1 | 0.01 | 1 | 0 | 0 | 0 | 1 | 1 | 0 | 1 | 0 | 0 | 0 |
| 123 | a-SSA/Ro IgG + a-SSB/La IgG + a-PR3 IgG (c-ANCA) | 3 | 1 | 0.01 | 0 | 0 | 1 | 1 | 0 | 0 | 0 | 0 | 1 | 0 | 0 |
| 124 | a-dsDNA IgG + a-Nuc IgG + a-U1-RNP IgG + a-Scl-70 IgG | 4 | 1 | 0.01 | 1 | 1 | 0 | 0 | 0 | 1 | 1 | 0 | 0 | 0 | 0 |
| 125 | a-dsDNA IgG + a-SSA/Ro IgG + a-Sm IgG | 3 | 1 | 0.01 | 1 | 0 | 1 | 0 | 1 | 0 | 0 | 0 | 0 | 0 | 0 |
| 126 | a-Nuc IgG + a-SSA/Ro IgG + a-Sm IgG + a-U1-RNP IgG + a-CL/β2GPI IgG/M | 5 | 1 | 0.01 | 0 | 1 | 1 | 0 | 1 | 1 | 0 | 0 | 0 | 0 | 1 |
| 127 | a-U1-RNP IgG + a-CL/β2GPI IgG/M | 2 | 1 | 0.01 | 0 | 0 | 0 | 0 | 0 | 1 | 0 | 0 | 0 | 0 | 1 |
| 128 | a-dsDNA IgG + a-Nuc IgG + a-MPO IgG (p-ANCA) + a-CL/β2GPI IgG/M | 4 | 1 | 0.01 | 1 | 1 | 0 | 0 | 0 | 0 | 0 | 0 | 0 | 1 | 1 |
| 129 | a-SSA/Ro IgG + a-Sm IgG + a-CL/β2GPI IgG/M | 3 | 1 | 0.01 | 0 | 0 | 1 | 0 | 1 | 0 | 0 | 0 | 0 | 0 | 1 |
| 130 | a-dsDNA IgG + a-SSA/Ro IgG + a-MPO IgG (p-ANCA) + a-CL/β2GPI IgG/M | 4 | 1 | 0.01 | 1 | 0 | 1 | 0 | 0 | 0 | 0 | 0 | 0 | 1 | 1 |
| 131 | a-Scl-70 IgG + a-PR3 IgG (c-ANCA) | 2 | 1 | 0.01 | 0 | 0 | 0 | 0 | 0 | 0 | 1 | 0 | 1 | 0 | 0 |
| 132 | a-dsDNA IgG + a-SSA/Ro IgG + a-MPO IgG (p-ANCA) | 3 | 1 | 0.01 | 1 | 0 | 1 | 0 | 0 | 0 | 0 | 0 | 0 | 1 | 0 |
| 133 | a-dsDNA IgG + a-Nuc IgG + a-SSA/Ro IgG + a-Sm IgG + a-PR3 IgG (c-ANCA) | 5 | 1 | 0.01 | 1 | 1 | 1 | 0 | 1 | 0 | 0 | 0 | 1 | 0 | 0 |
| 134 | a-U1-RNP IgG + a-Scl-70 IgG + a-Jo-1 IgG | 3 | 1 | 0.01 | 0 | 0 | 0 | 0 | 0 | 1 | 1 | 1 | 0 | 0 | 0 |
| 135 | a-dsDNA IgG + a-Nuc IgG + a-SSA/Ro IgG + a-MPO IgG (p-ANCA) | 4 | 1 | 0.01 | 1 | 1 | 1 | 0 | 0 | 0 | 0 | 0 | 0 | 1 | 0 |
| 136 | a-Jo-1 IgG + a-MPO IgG (p-ANCA) | 2 | 1 | 0.01 | 0 | 0 | 0 | 0 | 0 | 0 | 0 | 1 | 0 | 1 | 0 |
| 137 | a-Nuc IgG + a-U1-RNP IgG + a-MPO IgG (p-ANCA) | 3 | 1 | 0.01 | 0 | 1 | 0 | 0 | 0 | 1 | 0 | 0 | 0 | 1 | 0 |
| 138 | a-SSA/Ro IgG + a-SSB/La IgG + a-MPO IgG (p-ANCA) + a-CL/β2GPI IgG/M | 4 | 1 | 0.01 | 0 | 0 | 1 | 1 | 0 | 0 | 0 | 0 | 0 | 1 | 1 |
| 139 | a-dsDNA IgG + a-Nuc IgG + a-SSA/Ro IgG + a-SSB/La IgG + a-MPO IgG (p-ANCA) | 5 | 1 | 0.01 | 1 | 1 | 1 | 1 | 0 | 0 | 0 | 0 | 0 | 1 | 0 |
| 140 | a-Nuc IgG + a-SSA/Ro IgG + a-PR3 IgG (c-ANCA) | 3 | 1 | 0.01 | 0 | 1 | 1 | 0 | 0 | 0 | 0 | 0 | 1 | 0 | 0 |
| 141 | a-Nuc IgG + a-Scl-70 IgG + a-PR3 IgG (c-ANCA) | 3 | 1 | 0.01 | 0 | 1 | 0 | 0 | 0 | 0 | 1 | 0 | 1 | 0 | 0 |
| 142 | a-dsDNA IgG + a-Nuc IgG + a-PR3 IgG (c-ANCA) + a-MPO IgG (p-ANCA) | 4 | 1 | 0.01 | 1 | 1 | 0 | 0 | 0 | 0 | 0 | 0 | 1 | 1 | 0 |
| 143 | a-dsDNA IgG + a-Nuc IgG + a-Sm IgG + a-U1-RNP IgG + a-Jo-1 IgG | 5 | 1 | 0.01 | 1 | 1 | 0 | 0 | 1 | 1 | 0 | 1 | 0 | 0 | 0 |
| 144 | a-dsDNA IgG + a-Nuc IgG + a-Sm IgG + a-U1-RNP IgG + a-PR3 IgG (c-ANCA) + a-MPO IgG (p-ANCA) | 6 | 1 | 0.01 | 1 | 1 | 0 | 0 | 1 | 1 | 0 | 0 | 1 | 1 | 0 |
| 145 | a-dsDNA IgG + a-SSA/Ro IgG + a-Scl-70 IgG | 3 | 1 | 0.01 | 1 | 0 | 1 | 0 | 0 | 0 | 1 | 0 | 0 | 0 | 0 |
| 146 | a-Sm IgG + a-Jo-1 IgG + a-CL/β2GPI IgG/M | 3 | 1 | 0.01 | 0 | 0 | 0 | 0 | 1 | 0 | 0 | 1 | 0 | 0 | 1 |
| 147 | a-dsDNA IgG + a-SSA/Ro IgG + a-SSB/La IgG + a-CL/β2GPI IgG/M | 4 | 1 | 0.01 | 1 | 0 | 1 | 1 | 0 | 0 | 0 | 0 | 0 | 0 | 1 |
| 148 | a-SSA/Ro IgG + a-SSB/La IgG + a-Sm IgG + a-U1-RNP IgG + a-Scl-70 IgG + a-Jo-1 IgG | 6 | 1 | 0.01 | 0 | 0 | 1 | 1 | 1 | 1 | 1 | 1 | 0 | 0 | 0 |
| 149 | a-SSA/Ro IgG + a-MPO IgG (p-ANCA) | 2 | 1 | 0.01 | 0 | 0 | 1 | 0 | 0 | 0 | 0 | 0 | 0 | 1 | 0 |
| 150 | a-SSA/Ro IgG + a-SSB/La IgG + a-U1-RNP IgG + a-Scl-70 IgG + a-Jo-1 IgG | 5 | 1 | 0.01 | 0 | 0 | 1 | 1 | 0 | 1 | 1 | 1 | 0 | 0 | 0 |
| 151 | a-Nuc IgG + a-SSA/Ro IgG + a-Sm IgG + a-Scl-70 IgG + a-Jo-1 IgG | 5 | 1 | 0.01 | 0 | 1 | 1 | 0 | 1 | 0 | 1 | 1 | 0 | 0 | 0 |
| 152 | a-dsDNA IgG + a-Scl-70 IgG + a-Jo-1 IgG | 3 | 1 | 0.01 | 1 | 0 | 0 | 0 | 0 | 0 | 1 | 1 | 0 | 0 | 0 |
| 153 | a-Nuc IgG + a-SSA/Ro IgG + a-Sm IgG | 3 | 1 | 0.01 | 0 | 1 | 1 | 0 | 1 | 0 | 0 | 0 | 0 | 0 | 0 |
| 154 | a-dsDNA IgG + a-Nuc IgG + a-SSA/Ro IgG + a-SSB/La IgG + a-Scl-70 IgG | 5 | 1 | 0.01 | 1 | 1 | 1 | 1 | 0 | 0 | 1 | 0 | 0 | 0 | 0 |
| 155 | a-dsDNA IgG + a-Nuc IgG + a-SSA/Ro IgG + a-SSB/La IgG + a-Sm IgG + a-U1-RNP IgG + a-CL/β2GPI IgG/M | 7 | 1 | 0.01 | 1 | 1 | 1 | 1 | 1 | 1 | 0 | 0 | 0 | 0 | 1 |
| 156 | a-Nuc IgG + a-SSA/Ro IgG + a-SSB/La IgG + a-Sm IgG + a-U1-RNP IgG + a-CL/β2GPI IgG/M | 6 | 1 | 0.01 | 0 | 1 | 1 | 1 | 1 | 1 | 0 | 0 | 0 | 0 | 1 |
| 157 | a-Nuc IgG + a-Sm IgG + a-Scl-70 IgG + a-PR3 IgG (c-ANCA) + a-MPO IgG (p-ANCA) | 5 | 1 | 0.01 | 0 | 1 | 0 | 0 | 1 | 0 | 1 | 0 | 1 | 1 | 0 |
| 158 | a-SSA/Ro IgG + a-Sm IgG + a-Jo-1 IgG + a-CL/β2GPI IgG/M | 4 | 1 | 0.01 | 0 | 0 | 1 | 0 | 1 | 0 | 0 | 1 | 0 | 0 | 1 |
| 159 | a-Nuc IgG + a-Sm IgG + a-U1-RNP IgG + a-Jo-1 IgG + a-MPO IgG (p-ANCA) | 5 | 1 | 0.01 | 0 | 1 | 0 | 0 | 1 | 1 | 0 | 1 | 0 | 1 | 0 |
| 160 | a-Nuc IgG + a-SSA/Ro IgG + a-SSB/La IgG + a-U1-RNP IgG + a-CL/β2GPI IgG/M | 5 | 1 | 0.01 | 0 | 1 | 1 | 1 | 0 | 1 | 0 | 0 | 0 | 0 | 1 |
| 161 | a-SSB/La IgG + a-U1-RNP IgG | 2 | 1 | 0.01 | 0 | 0 | 0 | 1 | 0 | 1 | 0 | 0 | 0 | 0 | 0 |
| 162 | a-Nuc IgG + a-SSA/Ro IgG + a-SSB/La IgG + a-Sm IgG + a-U1-RNP IgG | 5 | 1 | 0.01 | 0 | 1 | 1 | 1 | 1 | 1 | 0 | 0 | 0 | 0 | 0 |
| 163 | a-dsDNA IgG + a-Nuc IgG + a-SSA/Ro IgG + a-SSB/La IgG + a-Sm IgG | 5 | 1 | 0.01 | 1 | 1 | 1 | 1 | 1 | 0 | 0 | 0 | 0 | 0 | 0 |
| 164 | a-Nuc IgG + a-SSA/Ro IgG + a-SSB/La IgG + a-Sm IgG | 4 | 1 | 0.01 | 0 | 1 | 1 | 1 | 1 | 0 | 0 | 0 | 0 | 0 | 0 |
| 165 | a-dsDNA IgG + a-PR3 IgG (c-ANCA) + a-MPO IgG (p-ANCA) + a-CL/β2GPI IgG/M | 4 | 1 | 0.01 | 1 | 0 | 0 | 0 | 0 | 0 | 0 | 0 | 1 | 1 | 1 |
| 166 | a-dsDNA IgG + a-SSA/Ro IgG + a-SSB/La IgG + a-Sm IgG + a-U1-RNP IgG | 5 | 1 | 0.01 | 1 | 0 | 1 | 1 | 1 | 1 | 0 | 0 | 0 | 0 | 0 |
| 167 | a-dsDNA IgG + a-SSA/Ro IgG + a-U1-RNP IgG + a-Jo-1 IgG | 4 | 1 | 0.01 | 1 | 0 | 1 | 0 | 0 | 1 | 0 | 1 | 0 | 0 | 0 |
| 168 | a-Sm IgG + a-U1-RNP IgG + a-CL/β2GPI IgG/M | 3 | 1 | 0.01 | 0 | 0 | 0 | 0 | 1 | 1 | 0 | 0 | 0 | 0 | 1 |
| 169 | a-dsDNA IgG + a-SSA/Ro IgG + a-Jo-1 IgG | 3 | 1 | 0.01 | 1 | 0 | 1 | 0 | 0 | 0 | 0 | 1 | 0 | 0 | 0 |
| 170 | a-dsDNA IgG + a-SSB/La IgG + a-CL/β2GPI IgG/M | 3 | 1 | 0.01 | 1 | 0 | 0 | 1 | 0 | 0 | 0 | 0 | 0 | 0 | 1 |
| 171 | a-SSA/Ro IgG + a-Jo-1 IgG + a-CL/β2GPI IgG/M | 3 | 1 | 0.01 | 0 | 0 | 1 | 0 | 0 | 0 | 0 | 1 | 0 | 0 | 1 |

**(C) Panel B intersections.** Figure 2B: all exclusive antibody combinations among the 2,447 a-dsDNA IgG-positive patients (a-nucleosome IgG not counted as an additional specificity), ranked by frequency. In the specificity columns, 1 = positive, 0 = negative or not tested.

| **Rank** | **Combination** | **Specificities (n)** | **Patients (n)** | **% of panel** | **a-dsDNA** | **a-SSA/Ro** | **a-SSB/La** | **a-Sm** | **a-U1-RNP** | **a-Scl-70** | **a-Jo-1** | **a-PR3** | **a-MPO** | **a-CL/β2GPI** |
| --- | --- | --- | --- | --- | --- | --- | --- | --- | --- | --- | --- | --- | --- | --- |
| 1 | a-dsDNA IgG only | 1 | 1704 | 69.64 | 1 | 0 | 0 | 0 | 0 | 0 | 0 | 0 | 0 | 0 |
| 2 | a-dsDNA IgG + a-CL/β2GPI IgG/M | 2 | 301 | 12.30 | 1 | 0 | 0 | 0 | 0 | 0 | 0 | 0 | 0 | 1 |
| 3 | a-dsDNA IgG + a-SSA/Ro IgG | 2 | 113 | 4.62 | 1 | 1 | 0 | 0 | 0 | 0 | 0 | 0 | 0 | 0 |
| 4 | a-dsDNA IgG + a-SSA/Ro IgG + a-SSB/La IgG | 3 | 69 | 2.82 | 1 | 1 | 1 | 0 | 0 | 0 | 0 | 0 | 0 | 0 |
| 5 | a-dsDNA IgG + a-Sm IgG + a-U1-RNP IgG | 3 | 31 | 1.27 | 1 | 0 | 0 | 1 | 1 | 0 | 0 | 0 | 0 | 0 |
| 6 | a-dsDNA IgG + a-U1-RNP IgG | 2 | 30 | 1.23 | 1 | 0 | 0 | 0 | 1 | 0 | 0 | 0 | 0 | 0 |
| 7 | a-dsDNA IgG + a-MPO IgG (p-ANCA) | 2 | 29 | 1.19 | 1 | 0 | 0 | 0 | 0 | 0 | 0 | 0 | 1 | 0 |
| 8 | a-dsDNA IgG + a-SSA/Ro IgG + a-CL/β2GPI IgG/M | 3 | 24 | 0.98 | 1 | 1 | 0 | 0 | 0 | 0 | 0 | 0 | 0 | 1 |
| 9 | a-dsDNA IgG + a-PR3 IgG (c-ANCA) | 2 | 14 | 0.57 | 1 | 0 | 0 | 0 | 0 | 0 | 0 | 1 | 0 | 0 |
| 10 | a-dsDNA IgG + a-SSB/La IgG | 2 | 13 | 0.53 | 1 | 0 | 1 | 0 | 0 | 0 | 0 | 0 | 0 | 0 |
| 11 | a-dsDNA IgG + a-SSA/Ro IgG + a-SSB/La IgG + a-CL/β2GPI IgG/M | 4 | 10 | 0.41 | 1 | 1 | 1 | 0 | 0 | 0 | 0 | 0 | 0 | 1 |
| 12 | a-dsDNA IgG + a-PR3 IgG (c-ANCA) + a-MPO IgG (p-ANCA) | 3 | 9 | 0.37 | 1 | 0 | 0 | 0 | 0 | 0 | 0 | 1 | 1 | 0 |
| 13 | a-dsDNA IgG + a-Scl-70 IgG | 2 | 7 | 0.29 | 1 | 0 | 0 | 0 | 0 | 1 | 0 | 0 | 0 | 0 |
| 14 | a-dsDNA IgG + a-SSA/Ro IgG + a-Sm IgG + a-U1-RNP IgG | 4 | 7 | 0.29 | 1 | 1 | 0 | 1 | 1 | 0 | 0 | 0 | 0 | 0 |
| 15 | a-dsDNA IgG + a-SSA/Ro IgG + a-Sm IgG + a-Jo-1 IgG | 4 | 6 | 0.25 | 1 | 1 | 0 | 1 | 0 | 0 | 1 | 0 | 0 | 0 |
| 16 | a-dsDNA IgG + a-Sm IgG + a-Jo-1 IgG | 3 | 6 | 0.25 | 1 | 0 | 0 | 1 | 0 | 0 | 1 | 0 | 0 | 0 |
| 17 | a-dsDNA IgG + a-U1-RNP IgG + a-CL/β2GPI IgG/M | 3 | 6 | 0.25 | 1 | 0 | 0 | 0 | 1 | 0 | 0 | 0 | 0 | 1 |
| 18 | a-dsDNA IgG + a-Jo-1 IgG | 2 | 6 | 0.25 | 1 | 0 | 0 | 0 | 0 | 0 | 1 | 0 | 0 | 0 |
| 19 | a-dsDNA IgG + a-Sm IgG | 2 | 6 | 0.25 | 1 | 0 | 0 | 1 | 0 | 0 | 0 | 0 | 0 | 0 |
| 20 | a-dsDNA IgG + a-SSA/Ro IgG + a-U1-RNP IgG | 3 | 5 | 0.20 | 1 | 1 | 0 | 0 | 1 | 0 | 0 | 0 | 0 | 0 |
| 21 | a-dsDNA IgG + a-MPO IgG (p-ANCA) + a-CL/β2GPI IgG/M | 3 | 5 | 0.20 | 1 | 0 | 0 | 0 | 0 | 0 | 0 | 0 | 1 | 1 |
| 22 | a-dsDNA IgG + a-SSA/Ro IgG + a-Scl-70 IgG | 3 | 4 | 0.16 | 1 | 1 | 0 | 0 | 0 | 1 | 0 | 0 | 0 | 0 |
| 23 | a-dsDNA IgG + a-SSA/Ro IgG + a-Jo-1 IgG | 3 | 3 | 0.12 | 1 | 1 | 0 | 0 | 0 | 0 | 1 | 0 | 0 | 0 |
| 24 | a-dsDNA IgG + a-SSA/Ro IgG + a-MPO IgG (p-ANCA) | 3 | 3 | 0.12 | 1 | 1 | 0 | 0 | 0 | 0 | 0 | 0 | 1 | 0 |
| 25 | a-dsDNA IgG + a-SSA/Ro IgG + a-SSB/La IgG + a-Sm IgG + a-U1-RNP IgG | 5 | 3 | 0.12 | 1 | 1 | 1 | 1 | 1 | 0 | 0 | 0 | 0 | 0 |
| 26 | a-dsDNA IgG + a-SSA/Ro IgG + a-SSB/La IgG + a-U1-RNP IgG | 4 | 3 | 0.12 | 1 | 1 | 1 | 0 | 1 | 0 | 0 | 0 | 0 | 0 |
| 27 | a-dsDNA IgG + a-PR3 IgG (c-ANCA) + a-MPO IgG (p-ANCA) + a-CL/β2GPI IgG/M | 4 | 2 | 0.08 | 1 | 0 | 0 | 0 | 0 | 0 | 0 | 1 | 1 | 1 |
| 28 | a-dsDNA IgG + a-Sm IgG + a-U1-RNP IgG + a-CL/β2GPI IgG/M | 4 | 2 | 0.08 | 1 | 0 | 0 | 1 | 1 | 0 | 0 | 0 | 0 | 1 |
| 29 | a-dsDNA IgG + a-Jo-1 IgG + a-CL/β2GPI IgG/M | 3 | 2 | 0.08 | 1 | 0 | 0 | 0 | 0 | 0 | 1 | 0 | 0 | 1 |
| 30 | a-dsDNA IgG + a-Sm IgG + a-U1-RNP IgG + a-Jo-1 IgG | 4 | 2 | 0.08 | 1 | 0 | 0 | 1 | 1 | 0 | 1 | 0 | 0 | 0 |
| 31 | a-dsDNA IgG + a-SSA/Ro IgG + a-Sm IgG | 3 | 2 | 0.08 | 1 | 1 | 0 | 1 | 0 | 0 | 0 | 0 | 0 | 0 |
| 32 | a-dsDNA IgG + a-SSA/Ro IgG + a-U1-RNP IgG + a-CL/β2GPI IgG/M | 4 | 2 | 0.08 | 1 | 1 | 0 | 0 | 1 | 0 | 0 | 0 | 0 | 1 |
| 33 | a-dsDNA IgG + a-SSA/Ro IgG + a-Sm IgG + a-U1-RNP IgG + a-CL/β2GPI IgG/M | 5 | 2 | 0.08 | 1 | 1 | 0 | 1 | 1 | 0 | 0 | 0 | 0 | 1 |
| 34 | a-dsDNA IgG + a-SSB/La IgG + a-Sm IgG | 3 | 1 | 0.04 | 1 | 0 | 1 | 1 | 0 | 0 | 0 | 0 | 0 | 0 |
| 35 | a-dsDNA IgG + a-SSA/Ro IgG + a-Sm IgG + a-Jo-1 IgG + a-CL/β2GPI IgG/M | 5 | 1 | 0.04 | 1 | 1 | 0 | 1 | 0 | 0 | 1 | 0 | 0 | 1 |
| 36 | a-dsDNA IgG + a-U1-RNP IgG + a-Scl-70 IgG | 3 | 1 | 0.04 | 1 | 0 | 0 | 0 | 1 | 1 | 0 | 0 | 0 | 0 |
| 37 | a-dsDNA IgG + a-SSA/Ro IgG + a-MPO IgG (p-ANCA) + a-CL/β2GPI IgG/M | 4 | 1 | 0.04 | 1 | 1 | 0 | 0 | 0 | 0 | 0 | 0 | 1 | 1 |
| 38 | a-dsDNA IgG + a-SSA/Ro IgG + a-Sm IgG + a-PR3 IgG (c-ANCA) | 4 | 1 | 0.04 | 1 | 1 | 0 | 1 | 0 | 0 | 0 | 1 | 0 | 0 |
| 39 | a-dsDNA IgG + a-SSA/Ro IgG + a-SSB/La IgG + a-MPO IgG (p-ANCA) | 4 | 1 | 0.04 | 1 | 1 | 1 | 0 | 0 | 0 | 0 | 0 | 1 | 0 |
| 40 | a-dsDNA IgG + a-SSA/Ro IgG + a-SSB/La IgG + a-Sm IgG + a-Jo-1 IgG | 5 | 1 | 0.04 | 1 | 1 | 1 | 1 | 0 | 0 | 1 | 0 | 0 | 0 |
| 41 | a-dsDNA IgG + a-Sm IgG + a-U1-RNP IgG + a-PR3 IgG (c-ANCA) + a-MPO IgG (p-ANCA) | 5 | 1 | 0.04 | 1 | 0 | 0 | 1 | 1 | 0 | 0 | 1 | 1 | 0 |
| 42 | a-dsDNA IgG + a-Scl-70 IgG + a-Jo-1 IgG | 3 | 1 | 0.04 | 1 | 0 | 0 | 0 | 0 | 1 | 1 | 0 | 0 | 0 |
| 43 | a-dsDNA IgG + a-SSA/Ro IgG + a-SSB/La IgG + a-Scl-70 IgG | 4 | 1 | 0.04 | 1 | 1 | 1 | 0 | 0 | 1 | 0 | 0 | 0 | 0 |
| 44 | a-dsDNA IgG + a-SSA/Ro IgG + a-SSB/La IgG + a-Sm IgG + a-U1-RNP IgG + a-CL/β2GPI IgG/M | 6 | 1 | 0.04 | 1 | 1 | 1 | 1 | 1 | 0 | 0 | 0 | 0 | 1 |
| 45 | a-dsDNA IgG + a-Sm IgG + a-CL/β2GPI IgG/M | 3 | 1 | 0.04 | 1 | 0 | 0 | 1 | 0 | 0 | 0 | 0 | 0 | 1 |
| 46 | a-dsDNA IgG + a-SSA/Ro IgG + a-SSB/La IgG + a-Sm IgG | 4 | 1 | 0.04 | 1 | 1 | 1 | 1 | 0 | 0 | 0 | 0 | 0 | 0 |
| 47 | a-dsDNA IgG + a-SSA/Ro IgG + a-U1-RNP IgG + a-Jo-1 IgG | 4 | 1 | 0.04 | 1 | 1 | 0 | 0 | 1 | 0 | 1 | 0 | 0 | 0 |
| 48 | a-dsDNA IgG + a-SSB/La IgG + a-Jo-1 IgG | 3 | 1 | 0.04 | 1 | 0 | 1 | 0 | 0 | 0 | 1 | 0 | 0 | 0 |
| 49 | a-dsDNA IgG + a-SSB/La IgG + a-CL/β2GPI IgG/M | 3 | 1 | 0.04 | 1 | 0 | 1 | 0 | 0 | 0 | 0 | 0 | 0 | 1 |

**(D) Venn diagram regions.** Figure 3: patients per region among a-dsDNA IgG-positive patients with exactly one (double positives, Figure 3A) or at least two (triple and higher, Figure 3B) additional antibodies.

| **Panel** | **Venn region** | **Patients (n)** | **% of panel** |
| --- | --- | --- | --- |
| A: double positives (n = 519) | aPL only | 301 | 58 |
| A: double positives (n = 519) | Ro/La only | 126 | 24.3 |
| A: double positives (n = 519) | Sm/U1-RNP only | 36 | 6.9 |
| A: double positives (n = 519) | Others (ANCA 43; Scl-70/Jo-1 13) | 56 | 10.8 |
| B: triple and higher (n = 224) | aPL only | 9 | 4 |
| B: triple and higher (n = 224) | Ro/La only | 82 | 36.6 |
| B: triple and higher (n = 224) | Sm/U1-RNP only | 41 | 18.3 |
| B: triple and higher (n = 224) | aPL + Ro/La | 36 | 16.1 |
| B: triple and higher (n = 224) | aPL + Sm/U1-RNP | 9 | 4 |
| B: triple and higher (n = 224) | Ro/La + Sm/U1-RNP | 31 | 13.8 |
| B: triple and higher (n = 224) | aPL + Ro/La + Sm/U1-RNP | 6 | 2.7 |
| B: triple and higher (n = 224) | Others (ANCA 9; Scl-70/Jo-1 1) | 10 | 4.5 |

**Notes**

Population: de-duplicated patients aged 18–80 years, regional diagnostic cohort 2014–2024 (n = 73,747).

Panel A (n = 7,721): patients positive for ≥1 of the 11 specificities (including a-nucleosome IgG). Each patient is represented by one sample: the first chronological sample with ≥2 positive specificities or, if there was none, the first positive sample.

Panel B (n = 2,447): a-dsDNA IgG-positive patients. Each patient is represented by the first chronological a-dsDNA IgG-positive sample that was also positive for ≥1 additional specificity; patients without such a sample are shown as "a-dsDNA IgG only". a-nucleosome IgG is not counted as an additional specificity in Panel B, as it targets the same antigen (chromatin) as a-dsDNA.

Order of selection: positive results flagged first → co-positive samples selected → sorted by patient and date → first chronological qualifying sample kept per patient.

Because only one sample per patient is used, per-specificity totals are lower than the analyte-by-analyte counts in Figure 1B (which count a co-positivity in any a-dsDNA IgG-positive sample).

Each patient is counted in exactly one combination. In the specificity columns, 1 = positive, 0 = negative or not tested.

Positivity: result above the laboratory-validated cut-off (Supplementary Table S1). c-ANCA and p-ANCA = a-PR3 and a-MPO IgG ELISA. a-CL/β2GPI IgG/M = a-CL and/or a-β2GPI IgG and/or IgM.

ANA screening and ENA screening were not included (screening assays); a-nucleosome IgG is shown in Panel A only.

Abbreviations: a-, anti-; aPL, antiphospholipid antibodies; ANA, antinuclear antibody; ANCA, anti-neutrophil cytoplasmic antibody; CI, confidence interval; CL, cardiolipin; β2GPI, β2-glycoprotein I; dsDNA, double-stranded DNA; ENA, extractable nuclear antigen; MPO, myeloperoxidase; Nuc, nucleosome; PR3, proteinase 3; PT, prothrombin; Scl-70, topoisomerase I; Sm, Smith; U1-RNP, U1-ribonucleoprotein.
