# Supplementary material for: Isotype-dependent shifts of natural autoantibody profiles in systemic lupus erythematosus-associated serological groups
Source: Front Immunol. 2026 Oct 30;17:1936421. doi: 10.3389/fimmu.2026.1936421 (PMC13759731; doi:10.3389/fimmu.2026.1936421)
Supplement: Supplementary file 3 [file Table3.docx]

**Supplementary Table S3.** Sex distribution of the cohort and of pAAb-positive patients, with crude and age-adjusted odds ratios for positivity in women versus men, and sex and age of a-dsDNA IgG-positive patients by the number of additional autoantibodies.

**(A) Sex distribution by marker.** Data underlying Supplementary Figures S2A and S2B. Patients tested within the corresponding diagnostic panel; OR = odds of positivity in women vs men.

| **Marker** | **Patients tested (n)** | **Women among tested (%)** | **Positive patients (n)** | **Women among positives, n (%)** | **Women among positives, 95% CI** | **Positivity in women (%)** | **Positivity in men (%)** | **OR women vs men (95% CI)** | **Age-adjusted OR, Mantel–Haenszel (95% CI)** | **p (age-adjusted)** |
| --- | --- | --- | --- | --- | --- | --- | --- | --- | --- | --- |
| ANA screen | 41,764 | 69.8 | 7,245 | 5702 (78.7%) | 77.7–79.6 | 19.56 | 12.24 | 1.74 (1.64–1.85) | 1.75 (1.64–1.86) | <0.001 |
| a-dsDNA IgG | 27,118 | 71.9 | 2,447 | 1978 (80.8%) | 79.2–82.4 | 10.15 | 6.15 | 1.72 (1.55–1.91) | 1.72 (1.55–1.91) | <0.001 |
| a-Nuc IgG | 31,590 | 71.8 | 3,672 | 3013 (82.1%) | 80.8–83.3 | 13.29 | 7.39 | 1.92 (1.76–2.10) | 1.92 (1.76–2.10) | <0.001 |
| a-CL/β2GPI IgG | 30,561 | 72.9 | 859 | 619 (72.1%) | 68.9–75.0 | 2.78 | 2.90 | 0.96 (0.82–1.11) | 0.97 (0.83–1.13) | 0.701 |
| a-CL/β2GPI IgM | 30,561 | 72.9 | 1,413 | 1072 (75.9%) | 73.5–78.1 | 4.81 | 4.12 | 1.17 (1.04–1.33) | 1.20 (1.06–1.36) | 0.004 |
| a-PT IgG | 22,727 | 75.0 | 630 | 492 (78.1%) | 74.7–81.3 | 2.89 | 2.43 | 1.19 (0.99–1.44) | 1.21 (1.00–1.47) | 0.056 |
| a-PT IgM | 22,727 | 75.0 | 367 | 290 (79.0%) | 74.5–83.1 | 1.70 | 1.36 | 1.26 (0.98–1.62) | 1.27 (0.99–1.64) | 0.071 |
| a-SSA/Ro IgG | 22,076 | 74.0 | 1,288 | 1126 (87.4%) | 85.5–89.2 | 6.90 | 2.82 | 2.55 (2.16–3.02) | 2.55 (2.16–3.01) | <0.001 |
| a-SSB/La IgG | 22,076 | 74.0 | 495 | 442 (89.3%) | 86.2–91.9 | 2.71 | 0.92 | 2.99 (2.24–3.98) | 2.97 (2.23–3.96) | <0.001 |
| a-Sm IgG | 22,076 | 74.0 | 265 | 215 (81.1%) | 75.9–85.7 | 1.32 | 0.87 | 1.52 (1.12–2.07) | 1.53 (1.12–2.09) | 0.008 |
| a-U1-RNP IgG | 22,076 | 74.0 | 303 | 256 (84.5%) | 79.9–88.4 | 1.57 | 0.82 | 1.93 (1.41–2.64) | 1.93 (1.41–2.63) | <0.001 |
| a-Scl-70 IgG | 22,076 | 74.0 | 142 | 118 (83.1%) | 75.9–88.9 | 0.72 | 0.42 | 1.74 (1.12–2.70) | 1.74 (1.12–2.70) | 0.017 |
| a-Jo-1 IgG | 22,076 | 74.0 | 310 | 230 (74.2%) | 68.9–79.0 | 1.41 | 1.39 | 1.01 (0.78–1.31) | 1.03 (0.79–1.33) | 0.897 |
| a-PR3 IgG (c-ANCA) | 32,299 | 66.6 | 380 | 213 (56.1%) | 50.9–61.1 | 0.99 | 1.55 | 0.64 (0.52–0.78) | 0.64 (0.52–0.78) | <0.001 |
| a-MPO IgG (p-ANCA) | 32,299 | 66.6 | 322 | 253 (78.6%) | 73.7–82.9 | 1.18 | 0.64 | 1.85 (1.41–2.41) | 1.81 (1.39–2.37) | <0.001 |

**(B) a-dsDNA IgG-positive patients by the number of additional autoantibodies.** Data underlying Supplementary Figure S2C; first co-positive sample of each patient.

| **Group (a-dsDNA IgG-positive patients)** | **Patients (n)** | **Women, n (%)** | **Age, median (IQR)** |
| --- | --- | --- | --- |
| a-dsDNA IgG only | 1,704 | 1338 (78.5%) | 57 (44–69) |
| Double positive (+1 antibody) | 519 | 441 (85.0%) | 57 (47–69) |
| Triple or higher (+≥2 antibodies) | 224 | 199 (88.8%) | 53 (44–67) |
| All a-dsDNA IgG-positive | 2,447 | 1978 (80.8%) | 56 (44–69) |

**Notes**

Cohort: 73,747 de-duplicated patients aged 18–80 years (2014–2024); women 48,429 (65.67%); median age 53 years (IQR 39–67).

Denominators and positivity definitions as in Figure 1A (patients tested within the corresponding diagnostic panel; first chronological qualifying result per patient).

Sex and age taken from the patient's first record; sex was consistent across all records of every patient.

OR = odds of positivity in women vs men among tested patients. Age adjustment: Mantel–Haenszel OR stratified by age group (18–39, 40–59, 60–80 years); p from the Cochran–Mantel–Haenszel test.

a-dsDNA groups: sex distribution differed between groups (χ² p < 0.001); age did not differ significantly (Kruskal–Wallis p = 0.079).

Abbreviations: OR, odds ratio; IQR, interquartile range; a-, anti-; aPL, antiphospholipid antibodies; ANA, antinuclear antibody; ANCA, anti-neutrophil cytoplasmic antibody; CI, confidence interval; CL, cardiolipin; β2GPI, β2-glycoprotein I; dsDNA, double-stranded DNA; ENA, extractable nuclear antigen; MPO, myeloperoxidase; Nuc, nucleosome; PR3, proteinase 3; PT, prothrombin; Scl-70, topoisomerase I; Sm, Smith; U1-RNP, U1-ribonucleoprotein.
