# Supplementary material for: Isotype-dependent shifts of natural autoantibody profiles in systemic lupus erythematosus-associated serological groups
Source: Front Immunol. 2026 Oct 30;17:1936421. doi: 10.3389/fimmu.2026.1936421 (PMC13759731; doi:10.3389/fimmu.2026.1936421)
Supplement: Supplementary file 4 [file Table4.docx]

**Supplementary Table S4.** Age and sex of the nAAb sub-study groups, and sensitivity analyses (women only, one sample per patient, and age- and sex-adjusted rank-based analysis of covariance) of all comparisons with seronegative controls. Additional parts: adjustment for the year of measurement, valid and missing sample numbers per nAAb analyte, post-hoc sensitivity power analysis, and other pAAb specificities in each group.

**(A) Demographics.** Sex and age per group; each pAAb-positive group vs seronegative controls (NC) (Fisher’s exact test for sex; Mann–Whitney U test for age).

| **Group** | **Samples (n)** | **Women, n (%)** | **Age, median (IQR)** | **Age range** | **p sex vs NC (Fisher)** | **p age vs NC (Mann–Whitney)** |
| --- | --- | --- | --- | --- | --- | --- |
| Seronegative controls (NC) | 39 | 39 (100.0%) | 45 (36–47) | 20–77 | — | — |
| a-SSA/Ro-dominant | 15 | 13 (86.7%) | 57 (45–64) | 32–74 | 0.073 | 0.006 |
| a-CL/β2GPI-dominant | 16 | 12 (75.0%) | 52 (43–60) | 36–81 | 0.005 | 0.003 |
| a-dsDNA/a-nucleosome-dominant | 14 | 14 (100.0%) | 51 (40–66) | 25–78 | 1.000 | 0.063 |
| a-SSA/Ro + a-dsDNA/a-nucleosome IgG | 14 | 13 (92.9%) | 45 (34–58) | 27–72 | 0.264 | 0.864 |
| a-CL/β2GPI + a-dsDNA/a-nucleosome IgG | 12 | 9 (75.0%) | 46 (34–53) | 21–63 | 0.011 | 0.609 |
| All six groups (overall test) |  | χ² p = 0.014 | Kruskal–Wallis p = 0.021 |  |  |  |

**(B) nAAb levels vs age and sex.** Spearman correlation of each nAAb with age, and women vs men (Mann–Whitney U test). All controls were women, so sex could be tested only across all samples, where it is confounded with group. Medians in AU/mL.

| **nAAb** | **Samples** | **n** | **Spearman ρ with age** | **p (age)** | **Men (n)** | **Median, women** | **Median, men** | **p (sex, Mann–Whitney)** |
| --- | --- | --- | --- | --- | --- | --- | --- | --- |
| a-CS IgG | Seronegative controls | 39 | 0.07 | 0.673 | 0 | 7.41 | — | — |
| a-CS IgG | All 110 samples | 108 | 0.08 | 0.440 | 9 | 8.73 | 19.00 | 0.188 |
| a-CS IgM | Seronegative controls | 39 | 0.04 | 0.805 | 0 | 20.85 | — | — |
| a-CS IgM | All 110 samples | 104 | −0.24 | 0.014 | 8 | 9.03 | 4.50 | 0.120 |
| a-HSP60 IgG | Seronegative controls | 36 | 0.14 | 0.407 | 0 | 6.70 | — | — |
| a-HSP60 IgG | All 110 samples | 104 | 0.13 | 0.180 | 9 | 8.70 | 14.69 | 0.090 |
| a-HSP60 IgM | Seronegative controls | 35 | −0.05 | 0.791 | 0 | 10.16 | — | — |
| a-HSP60 IgM | All 110 samples | 101 | 0.06 | 0.584 | 8 | 6.86 | 6.82 | 0.758 |
| a-HSP70 IgG | Seronegative controls | 38 | 0.06 | 0.733 | 0 | 6.54 | — | — |
| a-HSP70 IgG | All 110 samples | 107 | 0.09 | 0.373 | 9 | 7.48 | 11.56 | 0.149 |
| a-HSP70 IgM | Seronegative controls | 39 | −0.34 | 0.033 | 0 | 12.43 | — | — |
| a-HSP70 IgM | All 110 samples | 104 | −0.21 | 0.033 | 7 | 8.54 | 5.92 | 0.089 |

**(C) Sensitivity analyses.** Each pAAb-positive group vs NC for the three analyses of Figures 4–6. Arrow = direction relative to NC (↑ higher, ↓ lower); value = Holm-adjusted p. Original, women only and one sample per patient: Kruskal–Wallis test with Dunn’s post hoc test and Holm correction over all six pairwise comparisons of the figure; one sample per patient: patients contributing several samples within a figure were represented by their earliest sample only. Adjusted for age and sex: linear model on rank-transformed nAAb values with group, age and sex as covariates (rank-based ANCOVA); Holm correction over the three comparisons vs NC.

| **Figure** | **nAAb** | **Group vs NC** | **Original (all samples)** | **Women only** | **One sample per patient** | **Adjusted for age and sex (rank ANCOVA)** |
| --- | --- | --- | --- | --- | --- | --- |
| Figure 4 | a-CS IgG | a-SSA/Ro-dominant | ↑ 0.676 | ↑ 0.956 | ↑ 0.676 | ↑ 0.650 |
| Figure 4 | a-CS IgG | a-CL/β2GPI-dominant | ↑ 0.247 | ↑ 0.065 | ↑ 0.247 | ↑ 0.107 |
| Figure 4 | a-CS IgG | a-dsDNA/a-nucleosome-dominant | ↑ 0.004 | ↑ 0.002 | ↑ 0.004 | ↑ 0.004 |
| Figure 4 | a-CS IgM | a-SSA/Ro-dominant | ↓ <0.001 | ↓ <0.001 | ↓ <0.001 | ↓ <0.001 |
| Figure 4 | a-CS IgM | a-CL/β2GPI-dominant | ↓ <0.001 | ↓ <0.001 | ↓ <0.001 | ↓ <0.001 |
| Figure 4 | a-CS IgM | a-dsDNA/a-nucleosome-dominant | ↓ <0.001 | ↓ <0.001 | ↓ <0.001 | ↓ <0.001 |
| Figure 4 | a-HSP60 IgG | a-SSA/Ro-dominant | ↑ 0.277 | ↑ 0.412 | ↑ 0.277 | ↑ 0.241 |
| Figure 4 | a-HSP60 IgG | a-CL/β2GPI-dominant | ↑ 0.301 | ↑ 0.311 | ↑ 0.301 | ↑ 0.241 |
| Figure 4 | a-HSP60 IgG | a-dsDNA/a-nucleosome-dominant | ↑ <0.001 | ↑ <0.001 | ↑ <0.001 | ↑ <0.001 |
| Figure 4 | a-HSP60 IgM | a-SSA/Ro-dominant | ↓ 0.027 | ↓ 0.023 | ↓ 0.027 | ↓ 0.007 |
| Figure 4 | a-HSP60 IgM | a-CL/β2GPI-dominant | ↓ 0.651 | ↓ 0.843 | ↓ 0.651 | ↓ 0.322 |
| Figure 4 | a-HSP60 IgM | a-dsDNA/a-nucleosome-dominant | ↓ 0.038 | ↓ 0.033 | ↓ 0.038 | ↓ 0.008 |
| Figure 4 | a-HSP70 IgG | a-SSA/Ro-dominant | ↑ 0.741 | ↓ 0.837 | ↑ 0.741 | ↑ 0.701 |
| Figure 4 | a-HSP70 IgG | a-CL/β2GPI-dominant | ↑ 0.152 | ↑ 0.195 | ↑ 0.152 | ↑ 0.132 |
| Figure 4 | a-HSP70 IgG | a-dsDNA/a-nucleosome-dominant | ↑ <0.001 | ↑ <0.001 | ↑ <0.001 | ↑ <0.001 |
| Figure 4 | a-HSP70 IgM | a-SSA/Ro-dominant | ↓ 0.090 | ↓ 0.089 | ↓ 0.090 | ↓ 0.058 |
| Figure 4 | a-HSP70 IgM | a-CL/β2GPI-dominant | ↓ 0.090 | ↓ 0.345 | ↓ 0.090 | ↓ 0.107 |
| Figure 4 | a-HSP70 IgM | a-dsDNA/a-nucleosome-dominant | ↓ 0.024 | ↓ 0.021 | ↓ 0.024 | ↓ 0.015 |
| Figure 5 | a-CS IgG | a-SSA/Ro-dominant | ↑ 1.000 | ↑ 1.000 | ↑ 1.000 | ↓ 0.980 |
| Figure 5 | a-CS IgG | a-dsDNA/a-nucleosome-dominant | ↑ 0.002 | ↑ 0.002 | ↑ 0.002 | ↑ 0.002 |
| Figure 5 | a-CS IgG | a-SSA/Ro + a-dsDNA/a-nucleosome IgG | ↑ 0.012 | ↑ 0.031 | ↑ 0.003 | ↑ 0.005 |
| Figure 5 | a-CS IgM | a-SSA/Ro-dominant | ↓ <0.001 | ↓ <0.001 | ↓ <0.001 | ↓ <0.001 |
| Figure 5 | a-CS IgM | a-dsDNA/a-nucleosome-dominant | ↓ <0.001 | ↓ <0.001 | ↓ <0.001 | ↓ <0.001 |
| Figure 5 | a-CS IgM | a-SSA/Ro + a-dsDNA/a-nucleosome IgG | ↓ <0.001 | ↓ <0.001 | ↓ <0.001 | ↓ <0.001 |
| Figure 5 | a-HSP60 IgG | a-SSA/Ro-dominant | ↑ 0.383 | ↑ 0.550 | ↑ 0.409 | ↑ 0.400 |
| Figure 5 | a-HSP60 IgG | a-dsDNA/a-nucleosome-dominant | ↑ 0.001 | ↑ <0.001 | ↑ 0.001 | ↑ 0.001 |
| Figure 5 | a-HSP60 IgG | a-SSA/Ro + a-dsDNA/a-nucleosome IgG | ↑ 0.223 | ↑ 0.336 | ↑ 0.264 | ↑ 0.162 |
| Figure 5 | a-HSP60 IgM | a-SSA/Ro-dominant | ↓ 0.022 | ↓ 0.021 | ↓ 0.021 | ↓ 0.002 |
| Figure 5 | a-HSP60 IgM | a-dsDNA/a-nucleosome-dominant | ↓ 0.042 | ↓ 0.038 | ↓ 0.041 | ↓ 0.003 |
| Figure 5 | a-HSP60 IgM | a-SSA/Ro + a-dsDNA/a-nucleosome IgG | ↓ <0.001 | ↓ <0.001 | ↓ <0.001 | ↓ <0.001 |
| Figure 5 | a-HSP70 IgG | a-SSA/Ro-dominant | ↑ 0.984 | ↓ 1.000 | ↑ 1.000 | ↓ 0.919 |
| Figure 5 | a-HSP70 IgG | a-dsDNA/a-nucleosome-dominant | ↑ <0.001 | ↑ <0.001 | ↑ <0.001 | ↑ <0.001 |
| Figure 5 | a-HSP70 IgG | a-SSA/Ro + a-dsDNA/a-nucleosome IgG | ↑ 0.878 | ↑ 1.000 | ↑ 1.000 | ↑ 0.919 |
| Figure 5 | a-HSP70 IgM | a-SSA/Ro-dominant | ↓ 0.069 | ↓ 0.081 | ↓ 0.068 | ↓ 0.050 |
| Figure 5 | a-HSP70 IgM | a-dsDNA/a-nucleosome-dominant | ↓ 0.015 | ↓ 0.015 | ↓ 0.017 | ↓ 0.014 |
| Figure 5 | a-HSP70 IgM | a-SSA/Ro + a-dsDNA/a-nucleosome IgG | ↓ 0.069 | ↓ 0.086 | ↓ 0.066 | ↓ 0.050 |
| Figure 6 | a-CS IgG | a-CL/β2GPI-dominant | ↑ 0.248 | ↑ 0.098 | ↑ 0.232 | ↑ 0.090 |
| Figure 6 | a-CS IgG | a-dsDNA/a-nucleosome-dominant | ↑ 0.009 | ↑ 0.006 | ↑ 0.008 | ↑ 0.003 |
| Figure 6 | a-CS IgG | a-CL/β2GPI + a-dsDNA/a-nucleosome IgG | ↑ <0.001 | ↑ 0.009 | ↑ <0.001 | ↑ <0.001 |
| Figure 6 | a-CS IgM | a-CL/β2GPI-dominant | ↓ <0.001 | ↓ <0.001 | ↓ <0.001 | ↓ <0.001 |
| Figure 6 | a-CS IgM | a-dsDNA/a-nucleosome-dominant | ↓ <0.001 | ↓ <0.001 | ↓ <0.001 | ↓ <0.001 |
| Figure 6 | a-CS IgM | a-CL/β2GPI + a-dsDNA/a-nucleosome IgG | ↓ <0.001 | ↓ <0.001 | ↓ <0.001 | ↓ <0.001 |
| Figure 6 | a-HSP60 IgG | a-CL/β2GPI-dominant | ↑ 0.408 | ↑ 0.330 | ↑ 0.370 | ↑ 0.241 |
| Figure 6 | a-HSP60 IgG | a-dsDNA/a-nucleosome-dominant | ↑ <0.001 | ↑ <0.001 | ↑ <0.001 | ↑ <0.001 |
| Figure 6 | a-HSP60 IgG | a-CL/β2GPI + a-dsDNA/a-nucleosome IgG | ↑ 0.014 | ↑ 0.330 | ↑ 0.009 | ↑ 0.007 |
| Figure 6 | a-HSP60 IgM | a-CL/β2GPI-dominant | ↓ 1.000 | ↓ 1.000 | ↓ 1.000 | ↓ 0.262 |
| Figure 6 | a-HSP60 IgM | a-dsDNA/a-nucleosome-dominant | ↓ 0.039 | ↓ 0.033 | ↓ 0.045 | ↓ 0.011 |
| Figure 6 | a-HSP60 IgM | a-CL/β2GPI + a-dsDNA/a-nucleosome IgG | ↓ 0.398 | ↓ 0.456 | ↓ 0.381 | ↓ 0.206 |
| Figure 6 | a-HSP70 IgG | a-CL/β2GPI-dominant | ↑ 0.108 | ↑ 0.156 | ↑ 0.102 | ↑ 0.023 |
| Figure 6 | a-HSP70 IgG | a-dsDNA/a-nucleosome-dominant | ↑ <0.001 | ↑ <0.001 | ↑ <0.001 | ↑ <0.001 |
| Figure 6 | a-HSP70 IgG | a-CL/β2GPI + a-dsDNA/a-nucleosome IgG | ↑ 0.010 | ↑ 0.031 | ↑ 0.028 | ↑ 0.001 |
| Figure 6 | a-HSP70 IgM | a-CL/β2GPI-dominant | ↓ 0.089 | ↓ 0.393 | ↓ 0.084 | ↓ 0.065 |
| Figure 6 | a-HSP70 IgM | a-dsDNA/a-nucleosome-dominant | ↓ 0.028 | ↓ 0.030 | ↓ 0.029 | ↓ 0.021 |
| Figure 6 | a-HSP70 IgM | a-CL/β2GPI + a-dsDNA/a-nucleosome IgG | ↓ 0.028 | ↓ 0.061 | ↓ 0.056 | ↓ 0.021 |

**(D) Adjustment for the year of measurement.** As nAAb measurements were performed in different years (2014–2023), the year of measurement was added as a covariate to the rank-based linear model (group + year; group + year + age + sex); Holm correction over the comparisons vs NC. Arrow and value as in part (C); p (year) = p of the year covariate.

| **Figure** | **nAAb** | **Group vs NC** | **Original (Dunn–Holm)** | **Adjusted for year of measurement** | **Adjusted for year, age and sex** | **p (year) in year-only model** | **p (year) in full model** |
| --- | --- | --- | --- | --- | --- | --- | --- |
| Figure 4 | a-CS IgG | a-SSA/Ro-dominant | ↑ 0.676 | ↑ 0.636 | ↑ 0.619 | 0.764 | 0.640 |
| Figure 4 | a-CS IgG | a-CL/β2GPI-dominant | ↑ 0.247 | ↑ 0.140 | ↑ 0.157 | 0.764 | 0.640 |
| Figure 4 | a-CS IgG | a-dsDNA/a-nucleosome-dominant | ↑ 0.004 | ↑ 0.001 | ↑ 0.004 | 0.764 | 0.640 |
| Figure 4 | a-CS IgM | a-SSA/Ro-dominant | ↓ <0.001 | ↓ <0.001 | ↓ <0.001 | 0.874 | 0.720 |
| Figure 4 | a-CS IgM | a-CL/β2GPI-dominant | ↓ <0.001 | ↓ <0.001 | ↓ <0.001 | 0.874 | 0.720 |
| Figure 4 | a-CS IgM | a-dsDNA/a-nucleosome-dominant | ↓ <0.001 | ↓ <0.001 | ↓ <0.001 | 0.874 | 0.720 |
| Figure 4 | a-HSP60 IgG | a-SSA/Ro-dominant | ↑ 0.277 | ↑ 0.141 | ↑ 0.264 | 0.609 | 0.648 |
| Figure 4 | a-HSP60 IgG | a-CL/β2GPI-dominant | ↑ 0.301 | ↑ 0.141 | ↑ 0.264 | 0.609 | 0.648 |
| Figure 4 | a-HSP60 IgG | a-dsDNA/a-nucleosome-dominant | ↑ <0.001 | ↑ <0.001 | ↑ <0.001 | 0.609 | 0.648 |
| Figure 4 | a-HSP60 IgM | a-SSA/Ro-dominant | ↓ 0.027 | ↓ 0.020 | ↓ 0.013 | 0.201 | 0.175 |
| Figure 4 | a-HSP60 IgM | a-CL/β2GPI-dominant | ↓ 0.651 | ↓ 0.166 | ↓ 0.190 | 0.201 | 0.175 |
| Figure 4 | a-HSP60 IgM | a-dsDNA/a-nucleosome-dominant | ↓ 0.038 | ↓ 0.020 | ↓ 0.013 | 0.201 | 0.175 |
| Figure 4 | a-HSP70 IgG | a-SSA/Ro-dominant | ↑ 0.741 | ↑ 0.694 | ↑ 0.692 | 0.751 | 0.841 |
| Figure 4 | a-HSP70 IgG | a-CL/β2GPI-dominant | ↑ 0.152 | ↑ 0.076 | ↑ 0.167 | 0.751 | 0.841 |
| Figure 4 | a-HSP70 IgG | a-dsDNA/a-nucleosome-dominant | ↑ <0.001 | ↑ <0.001 | ↑ <0.001 | 0.751 | 0.841 |
| Figure 4 | a-HSP70 IgM | a-SSA/Ro-dominant | ↓ 0.090 | ↓ 0.031 | ↓ 0.068 | 0.834 | 0.821 |
| Figure 4 | a-HSP70 IgM | a-CL/β2GPI-dominant | ↓ 0.090 | ↓ 0.031 | ↓ 0.108 | 0.834 | 0.821 |
| Figure 4 | a-HSP70 IgM | a-dsDNA/a-nucleosome-dominant | ↓ 0.024 | ↓ 0.013 | ↓ 0.021 | 0.834 | 0.821 |
| Figure 5 | a-CS IgG | a-SSA/Ro-dominant | ↑ 1.000 | ↑ 0.532 | ↑ 0.988 | 0.673 | 0.674 |
| Figure 5 | a-CS IgG | a-dsDNA/a-nucleosome-dominant | ↑ 0.002 | ↑ <0.001 | ↑ 0.002 | 0.673 | 0.674 |
| Figure 5 | a-CS IgG | a-SSA/Ro + a-dsDNA/a-nucleosome IgG | ↑ 0.012 | ↑ 0.003 | ↑ 0.006 | 0.673 | 0.674 |
| Figure 5 | a-CS IgM | a-SSA/Ro-dominant | ↓ <0.001 | ↓ <0.001 | ↓ <0.001 | 0.623 | 0.662 |
| Figure 5 | a-CS IgM | a-dsDNA/a-nucleosome-dominant | ↓ <0.001 | ↓ <0.001 | ↓ <0.001 | 0.623 | 0.662 |
| Figure 5 | a-CS IgM | a-SSA/Ro + a-dsDNA/a-nucleosome IgG | ↓ <0.001 | ↓ <0.001 | ↓ <0.001 | 0.623 | 0.662 |
| Figure 5 | a-HSP60 IgG | a-SSA/Ro-dominant | ↑ 0.383 | ↑ 0.099 | ↑ 0.398 | 0.811 | 0.876 |
| Figure 5 | a-HSP60 IgG | a-dsDNA/a-nucleosome-dominant | ↑ 0.001 | ↑ <0.001 | ↑ 0.002 | 0.811 | 0.876 |
| Figure 5 | a-HSP60 IgG | a-SSA/Ro + a-dsDNA/a-nucleosome IgG | ↑ 0.223 | ↑ 0.074 | ↑ 0.189 | 0.811 | 0.876 |
| Figure 5 | a-HSP60 IgM | a-SSA/Ro-dominant | ↓ 0.022 | ↓ 0.005 | ↓ 0.004 | 0.366 | 0.363 |
| Figure 5 | a-HSP60 IgM | a-dsDNA/a-nucleosome-dominant | ↓ 0.042 | ↓ 0.006 | ↓ 0.005 | 0.366 | 0.363 |
| Figure 5 | a-HSP60 IgM | a-SSA/Ro + a-dsDNA/a-nucleosome IgG | ↓ <0.001 | ↓ <0.001 | ↓ <0.001 | 0.366 | 0.363 |
| Figure 5 | a-HSP70 IgG | a-SSA/Ro-dominant | ↑ 0.984 | ↑ 0.754 | ↓ 0.781 | 0.445 | 0.503 |
| Figure 5 | a-HSP70 IgG | a-dsDNA/a-nucleosome-dominant | ↑ <0.001 | ↑ <0.001 | ↑ <0.001 | 0.445 | 0.503 |
| Figure 5 | a-HSP70 IgG | a-SSA/Ro + a-dsDNA/a-nucleosome IgG | ↑ 0.878 | ↑ 0.357 | ↑ 0.715 | 0.445 | 0.503 |
| Figure 5 | a-HSP70 IgM | a-SSA/Ro-dominant | ↓ 0.069 | ↓ 0.031 | ↓ 0.091 | 0.607 | 0.731 |
| Figure 5 | a-HSP70 IgM | a-dsDNA/a-nucleosome-dominant | ↓ 0.015 | ↓ 0.009 | ↓ 0.021 | 0.607 | 0.731 |
| Figure 5 | a-HSP70 IgM | a-SSA/Ro + a-dsDNA/a-nucleosome IgG | ↓ 0.069 | ↓ 0.031 | ↓ 0.091 | 0.607 | 0.731 |
| Figure 6 | a-CS IgG | a-CL/β2GPI-dominant | ↑ 0.248 | ↑ 0.040 | ↑ 0.072 | 0.509 | 0.524 |
| Figure 6 | a-CS IgG | a-dsDNA/a-nucleosome-dominant | ↑ 0.009 | ↑ 0.002 | ↑ 0.005 | 0.509 | 0.524 |
| Figure 6 | a-CS IgG | a-CL/β2GPI + a-dsDNA/a-nucleosome IgG | ↑ <0.001 | ↑ <0.001 | ↑ <0.001 | 0.509 | 0.524 |
| Figure 6 | a-CS IgM | a-CL/β2GPI-dominant | ↓ <0.001 | ↓ <0.001 | ↓ <0.001 | 0.956 | 0.901 |
| Figure 6 | a-CS IgM | a-dsDNA/a-nucleosome-dominant | ↓ <0.001 | ↓ <0.001 | ↓ <0.001 | 0.956 | 0.901 |
| Figure 6 | a-CS IgM | a-CL/β2GPI + a-dsDNA/a-nucleosome IgG | ↓ <0.001 | ↓ <0.001 | ↓ <0.001 | 0.956 | 0.901 |
| Figure 6 | a-HSP60 IgG | a-CL/β2GPI-dominant | ↑ 0.408 | ↑ 0.045 | ↑ 0.132 | 0.186 | 0.229 |
| Figure 6 | a-HSP60 IgG | a-dsDNA/a-nucleosome-dominant | ↑ <0.001 | ↑ <0.001 | ↑ <0.001 | 0.186 | 0.229 |
| Figure 6 | a-HSP60 IgG | a-CL/β2GPI + a-dsDNA/a-nucleosome IgG | ↑ 0.014 | ↑ 0.001 | ↑ 0.004 | 0.186 | 0.229 |
| Figure 6 | a-HSP60 IgM | a-CL/β2GPI-dominant | ↓ 1.000 | ↓ 0.165 | ↓ 0.132 | 0.151 | 0.117 |
| Figure 6 | a-HSP60 IgM | a-dsDNA/a-nucleosome-dominant | ↓ 0.039 | ↓ 0.033 | ↓ 0.017 | 0.151 | 0.117 |
| Figure 6 | a-HSP60 IgM | a-CL/β2GPI + a-dsDNA/a-nucleosome IgG | ↓ 0.398 | ↓ 0.099 | ↓ 0.132 | 0.151 | 0.117 |
| Figure 6 | a-HSP70 IgG | a-CL/β2GPI-dominant | ↑ 0.108 | ↑ 0.009 | ↑ 0.018 | 0.454 | 0.473 |
| Figure 6 | a-HSP70 IgG | a-dsDNA/a-nucleosome-dominant | ↑ <0.001 | ↑ <0.001 | ↑ <0.001 | 0.454 | 0.473 |
| Figure 6 | a-HSP70 IgG | a-CL/β2GPI + a-dsDNA/a-nucleosome IgG | ↑ 0.010 | ↑ <0.001 | ↑ 0.001 | 0.454 | 0.473 |
| Figure 6 | a-HSP70 IgM | a-CL/β2GPI-dominant | ↓ 0.089 | ↓ 0.027 | ↓ 0.119 | 0.754 | 0.614 |
| Figure 6 | a-HSP70 IgM | a-dsDNA/a-nucleosome-dominant | ↓ 0.028 | ↓ 0.011 | ↓ 0.019 | 0.754 | 0.614 |
| Figure 6 | a-HSP70 IgM | a-CL/β2GPI + a-dsDNA/a-nucleosome IgG | ↓ 0.028 | ↓ 0.011 | ↓ 0.027 | 0.754 | 0.614 |

**(E) Valid and missing sample numbers.** Valid and missing sample numbers for each nAAb analyte in the final sub-study groups (110 samples), shown as valid n (missing n). Individual measurements could not be completed in a limited number of samples because of technical measurement issues or depletion of the residual serum; missing values were not imputed, and each analyte was analysed with its available valid observations.

| **nAAb / isotype** | **Seronegative controls (NC)** | **a-SSA/Ro-dominant** | **a-CL/β2GPI-dominant** | **a-dsDNA/a-nucleosome-dominant** | **a-SSA/Ro + a-dsDNA/a-nucleosome IgG** | **a-CL/β2GPI + a-dsDNA/a-nucleosome IgG** | **All samples** |
| --- | --- | --- | --- | --- | --- | --- | --- |
| a-CS IgG | 39 (0) | 15 (0) | 14 (2) | 14 (0) | 14 (0) | 12 (0) | 108 (2) |
| a-CS IgM | 39 (0) | 12 (3) | 14 (2) | 13 (1) | 14 (0) | 12 (0) | 104 (6) |
| a-HSP60 IgG | 36 (3) | 13 (2) | 15 (1) | 14 (0) | 14 (0) | 12 (0) | 104 (6) |
| a-HSP60 IgM | 35 (4) | 12 (3) | 15 (1) | 13 (1) | 14 (0) | 12 (0) | 101 (9) |
| a-HSP70 IgG | 38 (1) | 14 (1) | 15 (1) | 14 (0) | 14 (0) | 12 (0) | 107 (3) |
| a-HSP70 IgM | 39 (0) | 12 (3) | 14 (2) | 13 (1) | 14 (0) | 12 (0) | 104 (6) |
| Group size (samples) | 39 | 15 | 16 | 14 | 14 | 12 | 110 |

**(F1) Post-hoc sensitivity power analysis: smallest detectable effect.** Smallest effect detectable with 80% power (normal approximation for the Mann–Whitney/Dunn comparison; nominal group sizes). P(X > Y) = probability that a value of one group exceeds a value of the other (0.5 = no difference).

| **Type** | **Comparison (group sizes)** | **Two-sided α** | **Detectable P(X > Y), 80% power** | **Equivalent Cohen d** |
| --- | --- | --- | --- | --- |
| vs NC | NC (n = 39) vs a-SSA/Ro-dominant (n = 15) | 0.05 | 0.75 | 0.93 |
| vs NC | NC (n = 39) vs a-SSA/Ro-dominant (n = 15) | 0.0083 (0.05/6, strictest Holm step) | 0.81 | 1.22 |
| vs NC | NC (n = 39) vs a-CL/β2GPI-dominant (n = 16) | 0.05 | 0.74 | 0.91 |
| vs NC | NC (n = 39) vs a-CL/β2GPI-dominant (n = 16) | 0.0083 (0.05/6, strictest Holm step) | 0.80 | 1.18 |
| vs NC | NC (n = 39) vs a-dsDNA/a-nucleosome-dominant (n = 14) | 0.05 | 0.75 | 0.96 |
| vs NC | NC (n = 39) vs a-dsDNA/a-nucleosome-dominant (n = 14) | 0.0083 (0.05/6, strictest Holm step) | 0.81 | 1.26 |
| vs NC | NC (n = 39) vs a-SSA/Ro + a-dsDNA/a-nucleosome IgG (n = 14) | 0.05 | 0.75 | 0.96 |
| vs NC | NC (n = 39) vs a-SSA/Ro + a-dsDNA/a-nucleosome IgG (n = 14) | 0.0083 (0.05/6, strictest Holm step) | 0.81 | 1.26 |
| vs NC | NC (n = 39) vs a-CL/β2GPI + a-dsDNA/a-nucleosome IgG (n = 12) | 0.05 | 0.77 | 1.03 |
| vs NC | NC (n = 39) vs a-CL/β2GPI + a-dsDNA/a-nucleosome IgG (n = 12) | 0.0083 (0.05/6, strictest Holm step) | 0.83 | 1.36 |
| between pAAb-positive groups | a-SSA/Ro-dominant (n = 15) vs a-dsDNA/a-nucleosome-dominant (n = 14) | 0.05 | 0.80 | 1.19 |
| between pAAb-positive groups | a-SSA/Ro-dominant (n = 15) vs a-dsDNA/a-nucleosome-dominant (n = 14) | 0.0083 (0.05/6, strictest Holm step) | 0.87 | 1.62 |
| between pAAb-positive groups | a-dsDNA/a-nucleosome-dominant (n = 14) vs a-SSA/Ro + a-dsDNA/a-nucleosome IgG (n = 14) | 0.05 | 0.81 | 1.22 |
| between pAAb-positive groups | a-dsDNA/a-nucleosome-dominant (n = 14) vs a-SSA/Ro + a-dsDNA/a-nucleosome IgG (n = 14) | 0.0083 (0.05/6, strictest Holm step) | 0.88 | 1.66 |
| between pAAb-positive groups | a-dsDNA/a-nucleosome-dominant (n = 14) vs a-CL/β2GPI + a-dsDNA/a-nucleosome IgG (n = 12) | 0.05 | 0.82 | 1.28 |
| between pAAb-positive groups | a-dsDNA/a-nucleosome-dominant (n = 14) vs a-CL/β2GPI + a-dsDNA/a-nucleosome IgG (n = 12) | 0.0083 (0.05/6, strictest Holm step) | 0.90 | 1.77 |
| between pAAb-positive groups | a-CL/β2GPI-dominant (n = 16) vs a-CL/β2GPI + a-dsDNA/a-nucleosome IgG (n = 12) | 0.05 | 0.81 | 1.24 |
| between pAAb-positive groups | a-CL/β2GPI-dominant (n = 16) vs a-CL/β2GPI + a-dsDNA/a-nucleosome IgG (n = 12) | 0.0083 (0.05/6, strictest Holm step) | 0.88 | 1.69 |

**(F2) Observed effect sizes vs controls.** Observed effect sizes vs controls: P(group > NC) (values < 0.5 indicate lower levels in the pAAb-positive group; e.g. 0.07 means that a control exceeds a patient in 93% of pairs).

| **Group vs NC** | **a-CS IgG** | **a-CS IgM** | **a-HSP60 IgG** | **a-HSP60 IgM** | **a-HSP70 IgG** | **a-HSP70 IgM** |
| --- | --- | --- | --- | --- | --- | --- |
| a-SSA/Ro-dominant | 0.54 | 0.05 | 0.62 | 0.24 | 0.50 | 0.28 |
| a-CL/β2GPI-dominant | 0.64 | 0.09 | 0.64 | 0.42 | 0.70 | 0.30 |
| a-dsDNA/a-nucleosome-dominant | 0.84 | 0.07 | 0.89 | 0.23 | 0.88 | 0.22 |
| a-SSA/Ro + a-dsDNA/a-nucleosome IgG | 0.77 | 0.10 | 0.65 | 0.13 | 0.60 | 0.27 |
| a-CL/β2GPI + a-dsDNA/a-nucleosome IgG | 0.84 | 0.10 | 0.76 | 0.34 | 0.80 | 0.25 |

**(G) Other pAAb specificities in the final nAAb groups.** These specificities were not exclusion criteria. The six ENA antigens (a-SSA/Ro, a-SSB/La, a-Sm, a-U1-RNP, a-Scl-70, a-Jo-1) are measured together in one run; the panel, like ANCA and a-PT, was requested only in some patients outside the a-SSA/Ro groups. Values are positive/tested patients, in the nAAb sample itself and in any sample of the patient (2014–2024); last row: number of patients.

| **Specificity** | **Evaluated in** | **NC (n = 39)** | **a-SSA/Ro-dominant (n = 15)** | **a-CL/β2GPI-dominant (n = 16)** | **a-dsDNA/a-Nuc-dominant (n = 14)** | **a-SSA/Ro + a-dsDNA/a-Nuc (n = 14)** | **a-CL/β2GPI + a-dsDNA/a-Nuc (n = 12)** |
| --- | --- | --- | --- | --- | --- | --- | --- |
| a-SSB/La | nAAb sample | 0/0 | 6/15 | 0/1 | 0/2 | 6/11 | 0/3 |
|  | any sample 2014–2024 | 0/2 | 8/15 | 0/2 | 0/5 | 11/14 | 0/5 |
| a-Sm | nAAb sample | 0/0 | 2/15 | 0/1 | 0/2 | 1/11 | 1/3 |
|  | any sample 2014–2024 | 0/2 | 2/15 | 0/2 | 2/5 | 3/14 | 1/5 |
| a-U1-RNP | nAAb sample | 0/0 | 0/15 | 0/1 | 1/2 | 1/11 | 1/3 |
|  | any sample 2014–2024 | 0/2 | 0/15 | 0/2 | 2/5 | 2/14 | 1/5 |
| a-Scl-70 | nAAb sample | 0/0 | 0/15 | 0/1 | 0/2 | 0/11 | 0/3 |
|  | any sample 2014–2024 | 0/2 | 0/15 | 0/2 | 0/5 | 0/14 | 0/5 |
| a-Jo-1 | nAAb sample | 0/0 | 2/15 | 0/1 | 0/2 | 0/11 | 1/3 |
|  | any sample 2014–2024 | 0/2 | 2/15 | 0/2 | 1/5 | 0/14 | 1/5 |
| ANCA (a-MPO/a-PR3) | nAAb sample | 0/0 | 1/2 | 0/2 | 0/0 | 0/0 | 1/3 |
|  | any sample 2014–2024 | 0/1 | 1/2 | 1/2 | 0/3 | 1/2 | 1/5 |
| a-PT IgG/IgM | nAAb sample | 0/0 | 0/0 | 1/2 | 2/3 | 1/1 | 2/2 |
|  | any sample 2014–2024 | 0/1 | 0/0 | 2/3 | 3/6 | 3/3 | 2/2 |
| a-Sm and/or a-U1-RNP | positive on ≥ 2 dates | 0 | 0 | 0 | 2 | 2 | 1 |

**Notes**

Data: 110 serum samples of the six sub-study groups after patient-level review of the complete 2014–2024 diagnostic history (re-grouping by recurrent a-dsDNA, a-SSA/Ro and a-CL/β2GPI positivity; exclusion of 5 samples without documented a-dsDNA/a-nucleosome positivity [4 never a-dsDNA/a-nucleosome positive, 1 a-nucleosome positive only once, not in the nAAb sample], of 1 control with recurrent pAAb positivity and of 4 triple-positive patients; one sample per patient per group).

Sensitivity analyses (part C): across 54 comparisons, significance (p ≤ 0.05) was unchanged in 96% (women only), 98% (one sample per patient) and 94% (age/sex-adjusted) of cases; the direction of every difference significant in both analyses was preserved.

Adjustment for the year of measurement (part D): no significant difference of the main analysis was lost; the year of measurement was not associated with nAAb levels in any model (all p > 0.11); significance agreed with the age/sex-adjusted model in 96% of comparisons.

Abbreviations: a-, anti-; CL, cardiolipin; β2GPI, β2-glycoprotein I; CS, citrate synthase; dsDNA, double-stranded DNA; HSP, heat shock protein; IQR, interquartile range; NC, seronegative controls; nAAb, natural autoantibody; pAAb, pathological autoantibody; ANCOVA, analysis of covariance; PT, prothrombin.
